# A Complementarity‐Based Approach to *De Novo* Binder Design

**DOI:** 10.1002/advs.202502015

**Published:** 2025-07-21

**Authors:** Kateryna Maksymenko, Valeriia Hatskovska, Murray Coles, Narges Aghaallaei, Natalia Pashkovskaia, Natalia Borbarán‐Bravo, Matteo Pilz, Philip Bucher, Mareike Volz, Joana Pereira, Marcus D. Hartmann, Ghazaleh Tabatabai, Judith Feucht, Stefan Liebau, Patrick Müller, Andrei N. Lupas, Julia Skokowa, Mohammad ElGamacy

**Affiliations:** ^1^ Department of Protein Evolution Max Planck Institute for Biology 72076 Tübingen Germany; ^2^ Friedrich Miescher Laboratory of the Max Planck Society 72076 Tübingen Germany; ^3^ Division of Translational Oncology University Hospital Tübingen 72076 Tübingen Germany; ^4^ Ludwig Boltzmann Institute for Hematology and Oncology Medical University of Vienna Vienna 1090 Austria; ^5^ Institute of Neuroanatomy and Developmental Biology Eberhard Karls University Tübingen 72074 Tübingen Germany; ^6^ Applied Bioinformatics Department of Computer Science Eberhard Karls University Tübingen 72076 Tübingen Germany; ^7^ Institute for Bioinformatics and Medical Informatics Eberhard Karls University Tübingen 72076 Tübingen Germany; ^8^ Cluster of Excellence iFIT (EXC2180) “Image‐guided and Functionally Instructed Tumor Therapies” Eberhard Karls University Tübingen 72076 Tübingen Germany; ^9^ Department of Pediatric Hematology and Oncology Eberhard Karls University Tübingen University Children's Hospital 72076 Tübingen Germany; ^10^ VIB Center for AI and Computational Biology (VIB.AI), VIB Leuven 3000 Belgium; ^11^ Interfaculty Institute of Biochemistry Eberhard Karls University Tübingen 72076 Tübingen Germany; ^12^ Department of Neurology and Neuro‐Oncology, University Hospital Tübingen, Hertie Institute for Clinical Brain Research Eberhard Karls University Tübingen 72076 Tübingen Germany; ^13^ Department of Biology University of Konstanz 78457 Konstanz Germany; ^14^ Department of Cellular and Molecular Medicine KU Leuven Leuven 3000 Belgium

**Keywords:** complementarity evaluation, *de novo* binder design, IL‐7R binders, protein‐protein docking, VEGF inhibitors

## Abstract

*De novo* design of binders capable of targeting arbitrarily selected epitopes remains a substantial challenge. Here, a generalizable computational strategy is presented to design site‐specific protein binders, obviating steps of extensive empirical optimization or in vitro screening. The dock‐and‐design pipeline retrieves complementary scaffolds from a protein structure database to a given query epitope, where the scaffold is mutated to carve a binding site *de novo*. The docking step utilizes a novel fingerprint that greatly simplifies and accelerates the surface complementarity evaluation. As proof‐of‐concept, we designed protein binders to target three distinct epitopes on two different oncogenic targets; vascular endothelial growth factor (VEGF) and interleukin‐7 receptor‐α (IL‐7Rα). Experimental characterization of only 24 candidates identified nanomolar binders against each of the target epitopes, where the binders belonged to five different folds. Several designs were active in vitro. Moreover, anti‐VEGF designs showed tumor‐inhibiting activity in vivo, highlighting their therapeutic potential.

## Graphical abstract

A novel method for surface complementarity detection (HECTOR) enables highly efficient docking and design of protein interfaces. Applied to therapeutically relevant targets, this method yields *de novo* binders with potent antagonistic activity. As a first‐principles approach, HECTOR offers a training‐free solution to the binder design problem and is broadly applicable across diverse chemotypes and structures.

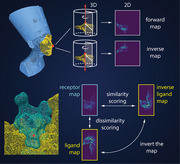

## 1 Introduction

Protein‐protein interactions play a central role in all biological processes; thus, the ability to design and manipulate such interactions is of immense value for basic and applied research. Particularly, the design of on‐demand binders against a predefined target epitope greatly broadens the range of accessible therapeutic applications.^[^ ^1^ ^]^ Such epitope targeting, in addition to the control over the binder's shape and biophysical properties, can be achieved through computational protein design.^[^ ^2^ ^]^ Previous studies have demonstrated successful protein binder design based on information derived from natural complex structures, where key interaction residues were incorporated into new, geometrically‐accommodating scaffolds.^[^ ^3^, ^4^, ^5^, ^6^ ^]^ Alternatively, *de novo* backbones have been built around natural binding motifs.^[^ ^7^, ^8^ ^]^ Although these template‐based approaches are rapidly advancing, they cover only a narrow range of targetable surfaces and are not applicable for novel interface design. Meanwhile, template‐free binder design – i.e., without using structural information from known binders – remains an outstanding challenge.^[^ ^9^ ^]^ Previous attempts to tackle this challenge have adopted different computational approaches. The earliest approach involved the docking of isolated residues against a target epitope, followed by mounting them onto a compatible backbone. As tested against diverse targets, this approach required the screening of thousands of candidates in order to identify a few nanomolar binders.^[^ ^10^, ^11^ ^]^ Another approach aimed to combine surface fingerprinting with deep neural networks to infer geometric and physicochemical complementarity, which has also resulted in a low success rate (<1%).^[^ ^12^ ^]^ Most recently, the sequential deployment of restrained diffusion and message‐passing neural networks was reported to generate binder backbone structures and decode fitting sequences, respectively. While this approach has demonstrably improved success rates, the involved neural networks, however, remain confined to the structural and binding patterns learned from available structure databases.^[^ ^13^ ^]^ In contrast, in this study, we aimed to develop a training‐free solution to the binder design problem.

**Figure 1:**
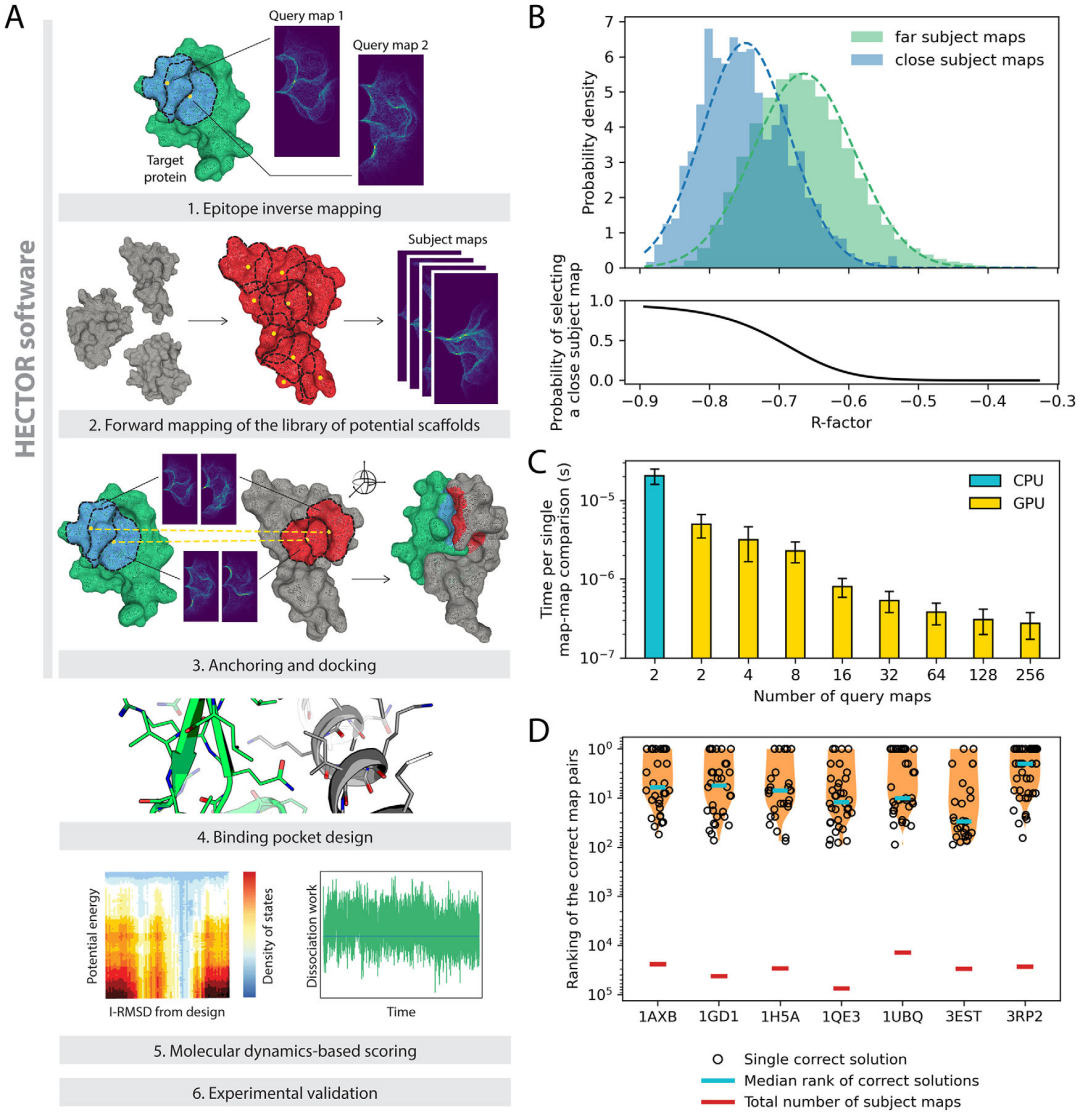
Strategy to design epitope‐directed binders using the HECTOR software. A) The design workflow primarily relies on simplifying the complexity of the docking process and decoupling it from the design stage. The docking step particularly, is based on a novel surface mesh fingerprinting protocol that analytically defines a complementary surface fingerprint and trivially matches it to a query surface. This is followed by sequence design and a two‐step molecular dynamics‐based testing of binding interface stability, utilizing serial tempering simulations (left pane) and steered MD (right pane). B) Histograms show distributions of R‐factor for close and far subject maps, where close maps are located within 1 Å and far maps are located more than 1 Å from their corresponding query maps selected at the interface of split proteins. As the R‐factor decreases, the probability of identifying a close subject map increases. C) The GPU implementation of R‐factor calculation greatly accelerates map‐to‐map comparisons, compared to CPU. As the number of query maps scales up, the evaluation time decreases even further. Evaluations were performed for 9 randomly selected proteins, where *n* maps from a query protein were compared versus all maps from a subject protein. Error bars represent the standard deviations across 9 analyzed proteins. D) The HECTOR algorithm allows to identify the correct pair of subject maps corresponding to the pair of query maps at the protein‐protein interfaces with high shape complementarity. The violin plot shows the distribution of the correct maps' rankings within the top 100 HECTOR hits.

An ideal binder must possess maximum binding affinity and specificity while being stable in the presence and absence of its target. This is a formidable challenge since improving one of these properties often comes at the cost of the other two.^[^ ^14^ ^]^ Moreover, binder design requires the simultaneous optimization of shape complementarity, polar and hydrophobic interactions at the interface, and the overall solvation energy of the bound and free forms. In this work, we explore a tiered approach that first prioritizes the maximization of shape complementarity, then follows to optimize and refine the other designable parameters. The first step of identifying complementary binding scaffolds is achieved through high‐throughput docking of a database of protein structures against the target epitope. To this end, we devised an ultra‐fast algorithm (Highly Efficient Complementarity Testing by Obverse Residuals, HECTOR), which enables the evaluation of surface complementarity through a vectorized, lower dimensional surface representation. The second step involves the sequence design and in silico‐filtering of the binding candidates (**Figure** **1**A).

To test the utility of our pipeline, we sought to design binders against therapeutically important targets. Specifically, we designed binders quenching the receptor‐binding site of vascular endothelial growth factor (VEGF) and two different epitopes on the ectodomains of the interleukin‐7 receptor‐α (IL‐7Rα). By testing a small number of designs, we could identify several nanomolar binders against all of the target epitopes, with a high success rate. Further characterization of the VEGF blockers showed their significant anti‐VEGF activity in vitro and in vivo. These results highlight the generalizability of our approach to design *de novo* binders from first principles, without reliance on training data or knowledge‐based scoring.

## 2 Results

### 2.1 Strategy for De Novo Design of Epitope‐targeted Binders

The first step of our pipeline aims at identifying binder scaffolds with highly complementary surface patches to the query epitope. Docking a query epitope against a large database of template structures is, however, a computationally demanding task, particularly given the rotational and translational degrees of freedom associated with the docking problem. In order to achieve ultra‐fast steric docking evaluation for two (query and subject) surface patches, we derived a surface fingerprint (i.e. map). The HECTOR fingerprint combines the two advantageous properties of *invertibility* and *compression*. Through the first property, a fingerprint describing a query surface patch can be inverted via a single transformation to describe the ideally complementary surface patch (Figure S1A, Supporting Information). Consequently, maximizing the similarity of the *inverted* query fingerprint to a subject fingerprint becomes a simple and straightforward optimization that effectively maximizes the complementarity between the underlying query and subject surfaces (Figure S1B, Supporting Information). The second property relates to the compression of a 3‐D surface patch into a 2‐D matrix using a cylindrical basis projection (Experimental Section, Figure S2, Supporting Information). While this compression is lossy, it is essential for achieving the rotational invariance of a fingerprint.

In this layout, a protein structure database is used as a source of scaffolds. Specifically, we used a snapshot of high‐resolution (better than 2.0 Å) X‐ray structures from PDB.^[^ ^15^ ^]^ The dot‐surfaces of all‐atom representations of these structures are derived (adding the coordinates of missing atoms) and stored. Overlapping surface patches are then extracted from these dot‐surfaces, which are then forward‐mapped to yield a database of fingerprints. While this large‐scale mapping tessellation and fingerprinting can be computationally expensive, it is performed only once. A query epitope (one or more) undergoes the same procedure, however, the *z*‐coordinate is obversely scaled (i.e. *k*  =   − 1) to obtain the inverse fingerprint (Experimental section). The complementarity between the query and subject surface patches is evaluated as an *R*‐factor that quantifies the dissimilarity between the two corresponding matrices (inverse query and forward subject fingerprints). Therefore, lower *R*‐factor values indicate higher complementarity (Figure 1B; Figure S1B, Supporting Information). Particularly, identifying clusters of query and subject surface patches that are spatially proximal can be used to identify putative binding sites. The *R*‐factor calculation could be efficiently implemented in a vectorized form on graphics processing units (GPUs), where a single evaluation fell within sub‐microsecond timeframe (Figure 1C).

Upon identifying scaffolds that harbor highly complementary surface patches, these patches would provide anchoring points for docked pose generation. In turn, generated complex models would serve as input for one‐sided interface design, where the scaffold residues at the binding interface are designed to optimize the interactions with the query epitope. To more rigorously rank the designed complexes, molecular dynamics (MD) simulations are used to predict the configurational stability and binding energy of the designed complexes.

### 2.2 Computational Design of the VEGF Binders

**Figure 2:**
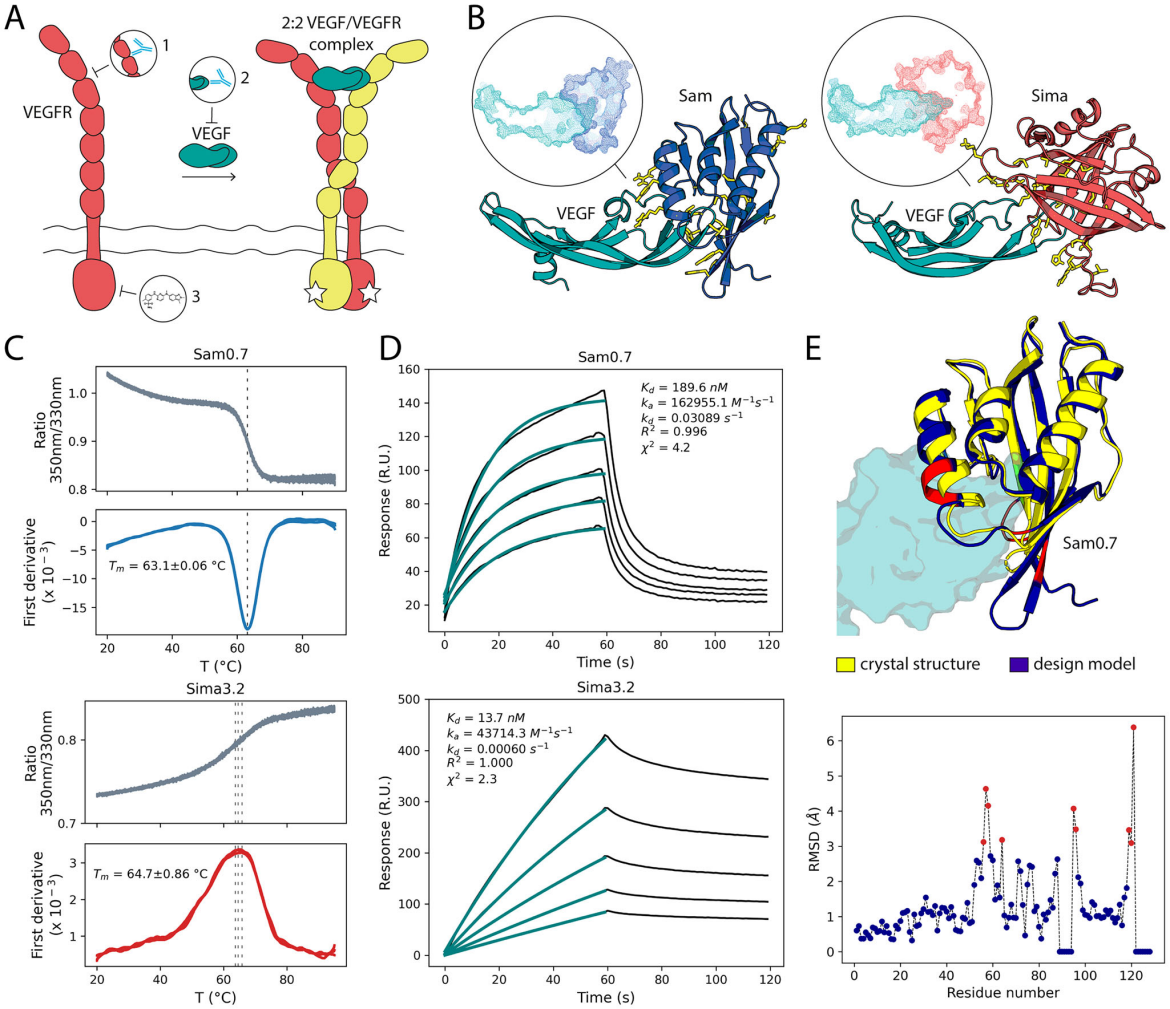
Design and biophysical characterization of the VEGF binders. A) VEGFR subunit is composed of 7 extracellular domains, a transmembrane segment, and an intracellular kinase domain. VEGFR is activated when dimeric VEGF binds, and in turn, dimerizes two receptor subunits. This dimeric receptor configuration triggers the intracellular domain kinase activity. VEGFR signaling can be inhibited via: 1) protein‐based quenching of the ligand binding site on the receptor surface, 2) protein‐based sequestration of VEGF itself at its receptor binding site, or 3) inhibition of the kinase activity through the use of small molecules. B) Anti‐VEGF binders were designed based on two different scaffolds, Sam (blue) and Sima (coral), that showed a high shape complementarity to the receptor‐binding site of VEGF (teal). Amino acids mutated during binder pocket design are colored in yellow. C) Thermal unfolding curves show melting temperatures (*T_m_*) of both Sam0.7 and Sima3.2 designs to be higher than 60 °C. D) The designed proteins bind VEGF with nanomolar affinity as measured by SPR. E) The crystal structure of Sam0.7 (yellow) matches the computational design model (blue) with atomic‐level accuracy. The VEGF epitope, in its modeled orientation relative to Sam0.7, is shown as a teal surface. The scatterplot displays RMSD between coordinates of the C_α_ atoms in the Sam0.7 design model and corresponding C_α_ atoms in the crystal structure. Residues in gaps are assigned an RMSD value of 0. Regions in the design model with RMSD values higher than 3 Å are highlighted in red.

Using the described strategy, we sought to design proteins able to prevent activation of the VEGF receptor (VEGFR) by quenching the receptor‐binding site of VEGF (**Figure** **2**A). VEGF forms a 2:2 complex with the ectodomain of VEGFR, which triggers its activation.^[^ ^16^ ^]^ Current anti‐VEGF therapies include both small molecules and protein‐based inhibitors. Small molecules act by inhibiting the VEGFR intracellular kinase domain; however, they are less specific and more toxic than the available protein‐based therapies.^[^ ^17^ ^]^ On the other hand, protein‐based inhibitors, which block the VEGF:VEGFR interaction, are large, complex molecules that are post‐translationally modified.^[^ ^18^, ^19^ ^]^ Therefore, we aimed to design VEGF inhibitors based on simple, single‐domain scaffolds.

To identify scaffolds with complementary shapes, we applied the HECTOR software to two arbitrarily selected surface patches at the receptor‐binding site of VEGF, which had been structurally characterized previously.^[^ ^20^, ^21^ ^]^ These surface patches were inverse‐mapped and docked against a library of fingerprints pre‐computed from a high‐resolution subset of PDB crystal structures.^[^ ^15^ ^]^ We retrieved the top six HECTOR hits (PDB: 1C7N, 1EYN, 1F73, 1KCD, 1OH0, 1PM1) and locally docked them against VEGF using PatchDock.^[^ ^22^ ^]^ We then performed interface design by designing positions on the binder structure chosen automatically based on their proximity from the target surface (C_α_ distance ≤ 8 Å). Sequence and conformers sampling was done using the RosettaScripts framework.^[^ ^23^ ^]^ After the design step, designs with the interface ddG higher than ‐30 REU or with the RosettaHoles score lower than 0.65 were discarded. Remaining designs were based on two templates: a bacterial ketosteroid isomerase (PDB: 1OH0) and a nitrophorin, hemoprotein of blood‐feeding insects (PDB: 1PM1). Both templates show a strong shape complementarity signal to the VEGF β‐hairpin loop at their respective catalytic pockets. Both also possess much simpler architecture than published anti‐VEGF antibodies and have molecular weights of 15 and 20 kDa, respectively. We named the designs utilizing the first scaffold Sam and those using the second one Sima (Figure 2B). The interface design was followed by two MD scoring steps, i.e. temperature‐accelerated MD and potential of mean force calculations from non‐equilibrium steered MD. We selected eight of the best‐scoring Sam designs (Sam0.1 – Sam0.8) for experimental validation. The final sequences (Figure S3, Supporting Information) have 19 to 24 mutations compared to the starting template. For Sima proteins, we chose four candidates for testing where the native disulfide bonds of the scaffold were retained (Sima1.1 – Sima4.1) and four candidates where the disulfide bonds were eliminated (Sima1.2 – Sima4.2). The final Sima sequences (Figure S3, Supporting Information) contain 25 – 28 mutations compared to the scaffold.

### 2.3 Biophysical Characterization of the Designed VEGF Inhibitors

We expressed the chosen designs in *E. coli* and double‐purified them, mostly with good yields (Table S1, Supporting Information). Based on expression yield and preliminarily estimated binding to VEGF (Figures S5 and S6, Supporting Information), we chose the best‐performing design from each group for full characterization: Sam0.7 and Sima3.2. Analytical size‐exclusion chromatography revealed that Sam0.7 is monomeric, while Sima3.2 exists in monomeric, dimeric, and tetrameric forms. Only the dimeric fractions of Sima3.2 were used for further experiments (Figure S7, Supporting Information). We evaluated the thermostability and aggregation propensity of the proteins adopting nano differential scanning fluorimetry and back reflection measurements. Sam0.7 and Sima3.2 have similar melting temperatures, 63 °C and 65 °C, respectively (Figure 2C). However, light scattering thermograms revealed that Sima3.2 has a higher propensity for aggregation with an onset temperature of ≈40 °C, compared to 60 °C for Sam0.7 (Figure S8, Supporting Information). To characterize the kinetics and affinity of interactions between the designed proteins and VEGF, we performed surface plasmon resonance (SPR)‐based binding assays. Analysis of the kinetics across the injection dilution series, assuming 1:1 binding, resulted in dissociation constants (*K_d_*) of 190 and 14 nM for Sam0.7 and Sima3.2, respectively. The higher affinity of Sima3.2 could be ascribed to its slower dissociation rate, compared to Sam0.7 (Figure 2D; Figure S9, Supporting Information).

### 2.4 Structural Characterization of the VEGF Binders

To evaluate the accuracy of the computational design, we sought to determine the experimental structures of the designed binders. We obtained crystal structures for the unbound Sam0.2 and Sam0.7 proteins. Alignment of the experimental structures to the corresponding design models showed that there is an atomic‐level agreement with overall backbone Cα root mean square deviation (RMSD) of 1.8 Å for Sam0.2 and 1.7 Å for Sam0.7 (Figure 2E; Figure S10 and Table S2, Supporting Information). Further analysis of the per‐residue deviations revealed that regions with the highest mismatch (Cα RMSD per residue > 3 Å) correspond to the outer rim of the Sam binding site (e.g., R56–M58, A64, F95, A96, D119–V121 for Sam0.7, Figure 2E). We ascribe this to the open and closed conformations of Sam in its bound and unbound states, respectively. While the designed coordinates of Sam were derived from its complex with VEGF (MD‐relaxed structure, Experimental section), the crystal structures represent isolated Sam monomers.

To further test whether Sam0.7 binds at the intended VEGF epitope, we performed SPR‐based competition assay, using bevacizumab, a highly potent and structurally validated anti‐VEGF antibody.^[^ ^24^, ^25^ ^]^ Similar to bevacizumab, Sam0.7 is designed to bind a VEGF epitope that partially overlaps with the VEGFR binding site (Figure S11A, Supporting Information). As expected, the results showed that Sam0.7 competed with bevacizumab for VEGF binding (Figure S11B, Supporting Information). Additionally, we introduced three mutations (P85R, H86G, G88R) to VEGF at its predicted interface with Sam0.7. SPR measurements confirmed that these mutations reduced binding to Sam0.7 (Figure S11C,D, Supporting Information).

### 2.5 In Vitro and in Vivo Evaluation of Anti‐VEGF Activity

**Figure 3:**
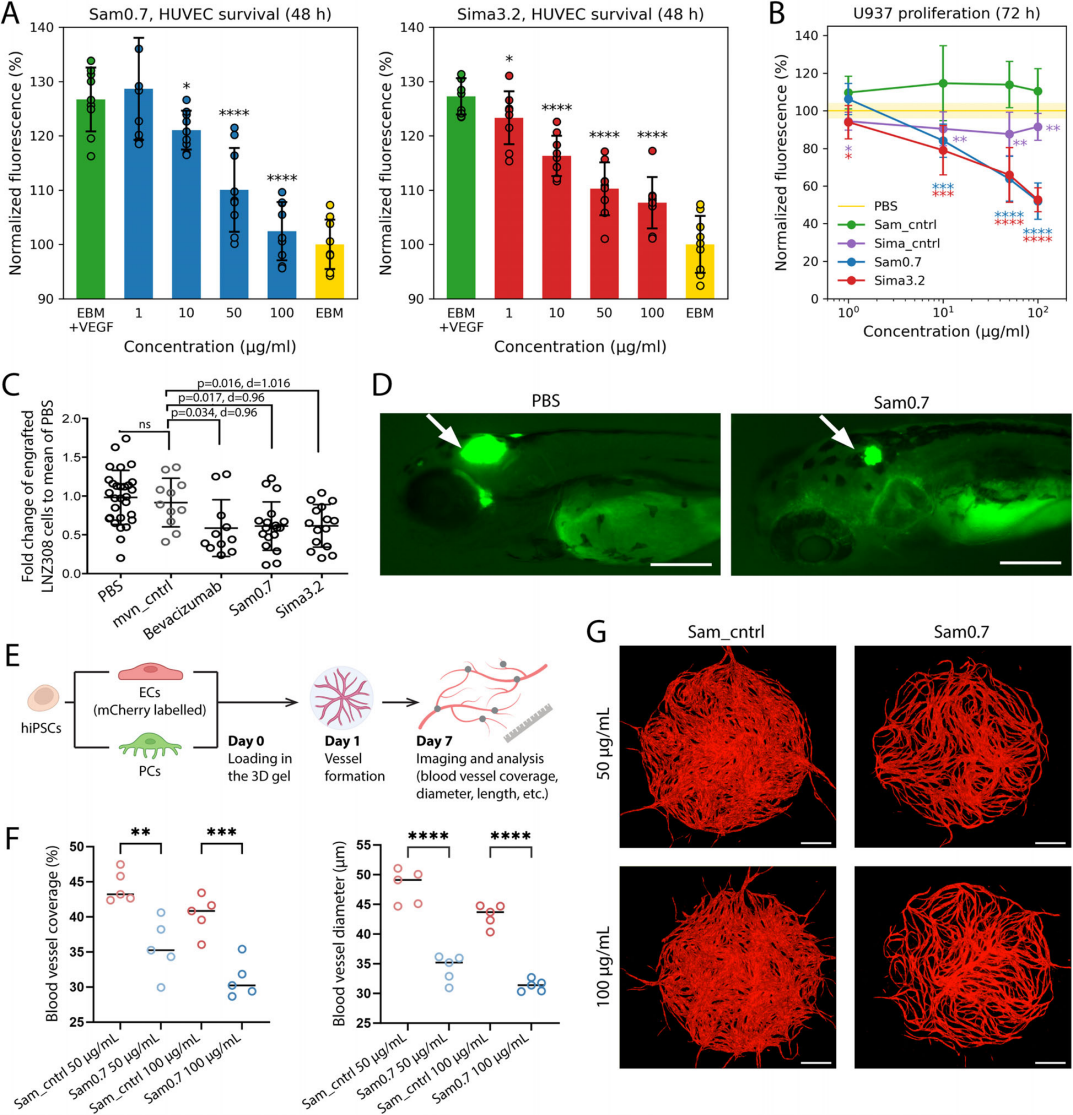
The designs exhibit anti‐VEGF activity in vitro and in vivo. A) VEGF‐dependent survival of HUVEC primary cells was significantly reduced in a dose‐dependent manner by the designed binders. Yellow bars indicate the survival of the cells in an endothelial cell growth basal medium without VEGF, whereas green bars correspond to the survival of the cells in a basal medium with 30 nM of VEGF. Blue and red bars show results on cell survival in a basal medium with 30 nm of VEGF and increasing concentrations of Sam0.7 and Sima3.2, respectively. B) Treatment of U937 acute myeloid leukemia cell line with Sam0.7 and Sima3.2 proteins at low micromolar concentrations inhibited the cell growth. In contrast, the unmutated scaffold Sima_cntrl showed much weaker inhibitory activity, while Sam_cntrl did not inhibit proliferation at all. Error bars represent the standard deviations across nine replicates from three experiments. Statistical significance was calculated using Fisher's one‐sided t‐test (^*^ *p* ≤ 0.05, ^**^ *p* ≤ 0.01, ^***^ *p* ≤ 0.001, ^****^ *p* ≤ 0.0001 vs. the PBS group). C) Quantification of the engrafted LNZ308‐GFP glioma cells in zebrafish embryos that were injected with PBS, inactive protein (mvn_cntrl) as a negative control, bevacizumab as a positive control, Sam 0.7, or Sima 3.2. Each dot indicates one embryo. *p*‐value was calculated by the Mann‐Whitney two‐tailed test. d – Cohen's d value. D) Representative zebrafish xenografts treated with PBS and Sam0.7. Arrowheads indicate transplanted LNZ308‐GFP cells in the brain. The scale bar is 200 µm. E) Schematic representation of in vitro microvasculature formation and analysis: iPSC‐derived endothelial cells (ECs) and pericytes (PCs) were co‐cultured in the fibrin gel with Sam0.7 or Sam_cntrl as negative control. The formed microvasculature was imaged at day 7, and images were analyzed to calculate microvasculature parameters. F) Quantitative analysis of microvasculature formation. Plots show the percentage of the area covered with blood vessels, and the blood vessel diameter. Statistical significance was calculated using the one‐way ANOVA test (^**^ *p* ≤ 0.01, ^***^ *p* ≤ 0.001, ^****^ *p* ≤ 0.0001 treated Sam0.7 vs. Sam_cntrl group). G) Representative images showing in vitro microvasculature formation in the presence of Sam0.7 or Sam_cntrl at two working concentrations (50 and 100 µg mL^−1^). The scale bar is 500 µm.

VEGF secreted by cancer cells provides mitogenic and survival stimuli for endothelial cells, leading to the formation of new blood vessels, and in turn, tumor expansion.^[^ ^26^ ^]^ In order to assess the inhibitory capability of the designed proteins, we tested their anti‐angiogenic effect on VEGF‐responsive primary human endothelial cells, HUVEC. Indeed, the addition of either Sam0.7 or Sima3.2 into a culture medium in the low micromolar range significantly decreased VEGF‐induced cell survival (**Figure** **3**A).

In addition to the regulation of angiogenesis, autocrine and paracrine VEGF/VEGFR signaling in tumor cells contributes to cancer cell proliferation, survival, induction of the epithelial‐mesenchymal transition, and metastasis.^[^ ^27^, ^28^ ^]^ For example, autocrine expression of VEGF has been reported to play a role in hematopoietic malignancies by stimulating the growth and migration of leukemic cells.^[^ ^29^ ^]^ We, therefore, decided to evaluate the efficacy of our designs on the proliferation of the acute myeloid leukemia cell line, U937, that is sensitive to VEGF. Treatment of U937 cells with increasing concentrations of the designed binders reduced cell proliferation down to 50% (Figure 3B). To test whether the detected anti‐VEGF activity of our designs is mediated by residues introduced during the interface design step, we performed U937 proliferation assay for the initial design templates of Sam and Sima (Sam_cntrl and Sima_cntrl, respectively). Sima_cntrl showed much weaker inhibitory activity compared to Sima3.2, while Sam_cntrl did not show any inhibitory effect (Figure 3B). Additionally, we evaluated the effect of the designed binders on the human embryonic kidney cell line, HEK293T, which does not express endogenous VEGFR. We observe a mild reduction in cell growth only after treatment of HEK293T cells with the highest concentration of Sam0.7 (100 µg mL^−1^), demonstrating that our designs specifically target VEGF‐dependent cells (Figure S12, Supporting Information).

After confirming the inhibitory effect of Sam0.7 on VEGF‐dependent cell types, we proceeded to evaluate its anti‐angiogenic activity, using an in vitro microvascular formation model. In this experiment, a self‐assembled 3D microvasculature was created by co‐culturing human induced pluripotent stem cell (hiPSC)‐derived endothelial cells and pericytes on a 3D fibrin gel. The microvasculature was treated with either Sam0.7 or Sam_cntrl (Figure 3E). Treatment with Sam0.7 at both tested concentrations (50 and 100 µg mL^−1^) significantly reduced the angiogenesis in vitro in a dose‐dependent manner (Figure 3F,G). Specifically, the microvasculature treated with Sam0.7 exhibited lower area coverage and thinner blood vessels compared to the microvasculature treated with Sam_cntrl. Other microvascular parameters, such as connectivity, total vessel length, amount of branches, and others remained unchanged (Figure S13, Supporting Information). Moreover, Sam0.7 showed a stronger effect on the microvasculature formation than 100 µg mL^−1^ bevacizumab (Figure S14, Supporting Information).

To further evaluate the pharmacological potential of the designs, we studied their effects on the VEGF‐dependent solid tumor and blood cancer cells in vivo. Injection of 3.5 mg mL^−1^ (4 nL) of Sam0.7 into the brain of zebrafish embryos at 33 h post fertilization (hpf) led to a decrease in the number of transplanted LNZ308 glioma cells after 2 days of treatment at a comparable level to injection of 25 mg mL^−1^ of bevacizumab (Figure 3C,D). A similar effect was observed when the design was tested on a leukemic cell line. Injection of Sam0.7 into the bloodstream of zebrafish embryos, where U937 leukemia cells were engrafted, led to a reduction in their number (Figure S15, Supporting Information). Treatment of zebrafish embryos with 2.4 mg mL^−1^ (4 nL) of Sima3.2 over 2 days had a significant inhibiting effect on LNZ308 cells when it was injected directly into the brain (Figure 3C). However, the injection of Sima3.2 into the bloodstream of embryos did not reduce U937 cells and showed a high lethality rate as opposed to PBS control and Sam0.7 injections (Figure S15, Supporting Information). This we attribute to the higher aggregation propensity of Sima3.2 in solution compared to Sam0.7.

### 2.6 Streamlining the HECTOR Docking Software

With experience gained from the design of VEGF binders, we sought to further improve the accuracy of complementary template searching and fully automate the template docking process. First, we aimed to optimize HECTOR mapping parameters by exploring how molecular surface inflation, and mapping frequency, density, size, and resolution influence the docking enrichment factors over a dataset of protein interfaces. We derived these interfaces from a set of seven split proteins (Figure S16 and Table S3, Supporting Information), where their well‐packed cores provide surfaces with strong steric complementarity. This is in contrast to natural protein: protein complexes, which often feature interface irregularities (Figure S17, Supporting Information). The docking involved a two‐vs‐all search using 45 unique query patch pairs as independent docking queries per split protein. Here, the search consisted of five steps: 1) identifying *N* subject maps with the lowest R‐factor relative to the first query map; 2) identifying *N* subject maps with the lowest R‐factor relative to the second query map; 3) filtering the identified subject map pairs by distance constraints; 4) calculating the RMSD between query and subject pairs as aligned by the two patch centers and their corresponding surface normals; and 5) ranking the selected subject pairs using a combined score of average R‐factor and RMSD. To analyze the search results, we evaluated how many true positive subject pairs were found within the top 100 hits and how they ranked. We achieved the best performance using maps with a radius of 10 Å, a height of 20 Å, and a resolution of 0.2 Å. We observed that the raised map resolution, combined with the highest mapping density and a mapping frequency of 1:5, eliminates the need for map interpolation that was previously performed. To account for the uncertainty of the surface coordinates, we also applied molecular surface inflation of 0.5 Å before map generation to improve the docking accuracy. Additionally, we introduced sigmoid radial fading of the maps with respect to the patch center, which reduced the weight of the contribution of the patch edges. Lastly, we revised the R‐factor formula to maximize the normalized overlap between query and subject maps (Experimental section). In more than 70% of cases, the combination of the described parameters led to identifying the correct subject pair within the top 100 retrieved pairs, out of tens of thousands of total subject maps. Frequently, these correct solutions were ranked within the top 10 (Figure 1D; Figure S17, Supporting Information).

To further enhance the docking specificity, we applied a four‐step filtering of subject patch pairs. The first filter is an average R‐factor cutoff that is ideally set to ≤ ‐0.8 (Figure 1B). The second is a tolerance to the deviation of the distance between the centers of the query patches and the corresponding subject patches. The third is an RMSD cutoff of the alignment of four‐point clique representing the two patch centers and their corresponding surface normals, of the query and subject pairs. While this RMSD score reports on both patch‐pair spacing and relative patch‐pair orientation, the relative weight of the orientation can be rescaled either manually, or normalized by the patch‐pair spacing. The same RMSD calculation is used to align the subject and query structures, whereby the fourth filtering step favors inter‐chain proximity and disfavors steric clashes as computed by tensorized kernels of the Damietta software.^[^ ^30^ ^]^

Finally, we sought to test if further compression of our fingerprint could be achieved, in order to speed up complementarity evaluation and reduce the required memory and I/O loads. Using a convolutional autoencoder we could compress our maps into a 128‐element vector, which still captures complementarity information. This can be implemented for a pre‐filtering of putatively complementary maps before full R‐factor calculation (Figure S18, Supporting Information).

### 2.7 Computational Design of the IL‐7Rα Binders

**Figure 4:**
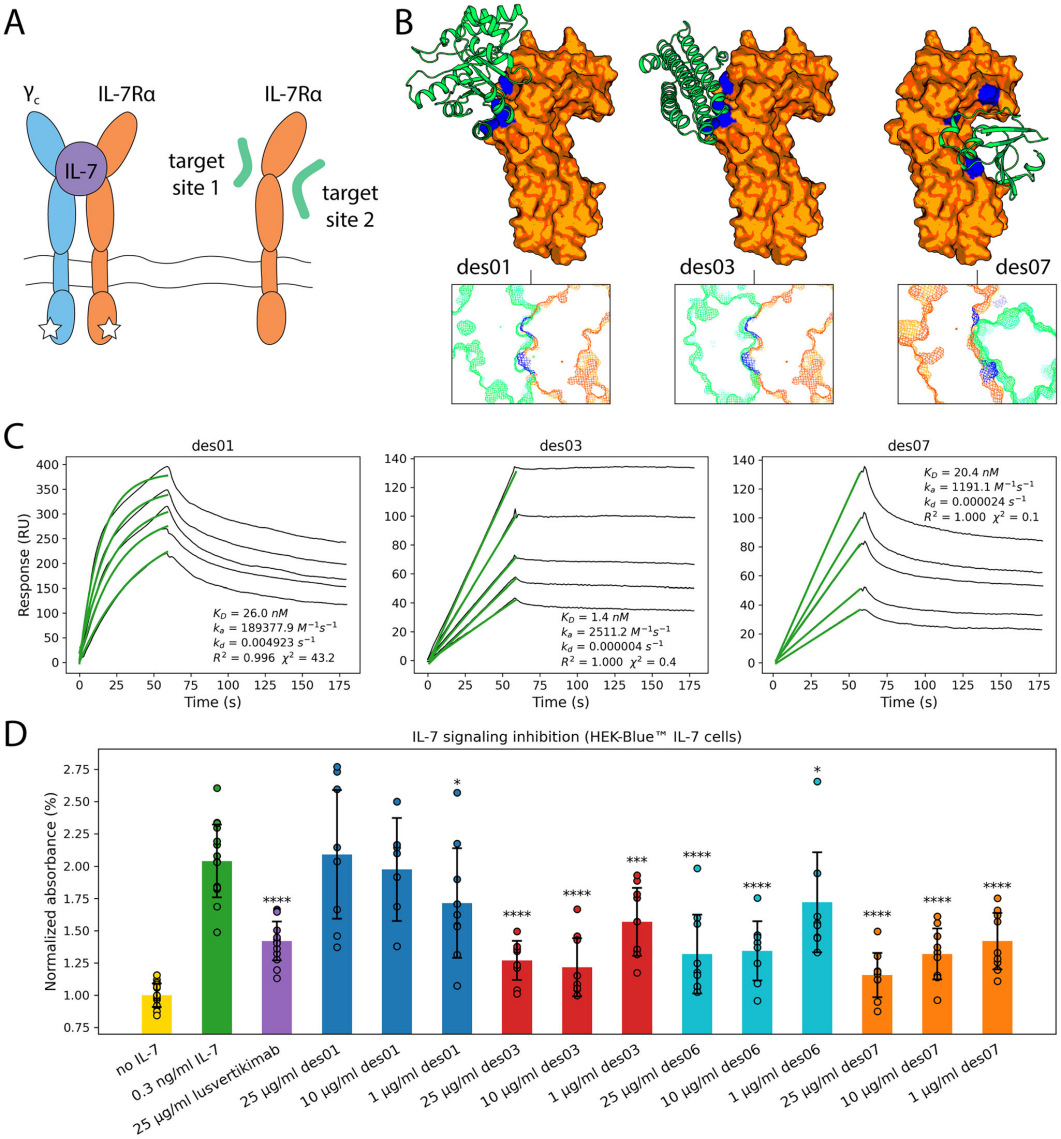
Design and experimental validation of the IL‐7Rα binders. A) IL‐7Rα binds IL‐7, leading to the recruitment of the γ_c_ receptor. The interaction between these three components is crucial for signaling cascades involved in the development and homeostasis of T and B cells. The designed binders are intended to target two distinct sites on the surface of IL‐7Rα. B) IL‐7Rα binders were designed using three different scaffolds that showed high shape complementarity to either target site 1 or site 2. The designs are shown in green, IL‐7Rα in orange, and the query patches in blue. C) SPR sensograms show the designed proteins to bind IL‐7Rα with nanomolar affinities. D) des03, des06, and des07 inhibited IL‐7 signaling in HEK‐Blue^TM^ IL‐7 reporter cells, similarly to lusvertikimab, an anti‐IL‐7Rα antibody. Statistical significance was calculated using Fisher's one‐sided *t*‐test (^*^ *p* ≤ 0.05, ^**^ *p* ≤ 0.01, ^***^ *p* ≤ 0.001, ^****^ *p* ≤ 0.0001 vs. the group treated with IL‐7 only).

To test our updated computational pipeline, we designed *de novo* binders against IL‐7Rα. Interleukin‐7 (IL‐7), IL‐7Rα, and γ_c_ receptors form a ternary complex essential for signaling pathways involved in normal lymphoid development and leukemogenesis.^[^ ^31^ ^]^ Therapeutics targeting the IL‐7/IL‐7R axis may, therefore, benefit patients with acute lymphoblastic leukemia or autoimmune diseases.^[^ ^32^ ^]^ We aimed to target two distinct IL‐7Rα epitopes, that we refer to as site 1 and site 2 (**Figure** **4**A). Site 1 overlaps with the native binding site of IL‐7, whereas site 2 has no known binders. Developing proteins targeting these two sites can provide two alternative ways of engaging the receptor; in competition with or orthogonal to native ligand binding. To find design scaffolds, we defined three distinct query patches at each of the target sites (Figure 4B). The patches were selected to ensure at least a 10 Å distance between their centers, which we found to improve the specificity of the search. Additionally, we avoided the glycosylation sites within the selected query patch areas. Complementarity searches were performed on all unique pairwise combinations of three patches. We selected five top‐ranking HECTOR hits for each target site (Experimental section) and designed the binding interface using the Damietta engine.^[^ ^30^, ^33^ ^]^ After the first design round, we performed temperature‐accelerated MD scoring of interface stability, and discarded scaffolds with unstable interfaces. Two scaffolds for site 1 (PDB: 5NLC, 6B8F) and one scaffold for site 2 (PDB: 6YUD) remained at this stage. These final scaffolds were a TIM barrel, a four‐helix bundle, and a protein with the Rossmann fold, respectively. Notably, the retrieved complementary surface of the 6YUD scaffold coincided with its native dimerization interface. Additionally, the 6B8F scaffold was reported to assemble into a quasi‐spherical 24‐mer, which we hypothesized might beneficially contribute to binder avidity (Figure S19, Supporting Information). The top‐ranked designs underwent a second round of design with Damietta and final MD scoring. 8 designs were prioritized for experimental characterization: des01, des02 based on the 5NLC template; des03‐des06 based on the 6B8F template; and des07, des08 based on the 6YUD template (Figure 4B; Figure S20, Supporting Information). A detailed design filtering scheme is shown in Figure S21 (Supporting Information).

### 2.8 Experimental Characterization of the Designed IL‐7Rα Binders

We expressed 8 anti‐IL‐7Rα designs, along with their initial templates (5NLC, 6B8F, 6YUD), and double‐purified them (Figure S22 and Table S4, Supporting Information). We excluded des02 from subsequent testing, as it precipitated at concentrations above 0.2 mg mL^−1^. 6 out of 7 remaining designs (all except des05) showed binding to IL‐7Rα in a single‐injection SPR experiment. Therefore, we proceeded with further characterization of these 6 designs. We used monomeric fractions for des01, and des08; dimeric fractions for des07; and 24‐mers for des03, des04, and des06 (Figure S22, Supporting Information). While des01 exhibited a melting temperature of 51 °C, all other tested proteins showed melting temperatures above 100 °C, as assessed by nano‐scale differential scanning fluorimetry (Figure S23, Supporting Information). To evaluate affinity of the designs to their target, we performed SPR‐based binding assays. Analysis of the titration series revealed that all tested designs bind IL‐7Rα with nanomolar affinity (Figure 4C; Figures S25 and S26, Supporting Information). Interestingly, while the unmutated 6B8F scaffold does not show any interaction with IL‐7Rα, the 5NLC and 6YUD scaffolds bind IL‐7Rα with *K_d_* of 1.3 µm and 723 nm, respectively. However, those interactions have much faster dissociation rates compared to the designs (Figure S24, Supporting Information).

For functional validation of the designs, we performed an in vitro assay using commercially obtained HEK‐Blue IL‐7 reporter cells engineered to detect the activation of JAK/STAT pathway in response to IL‐7. As expected, treatment with des03 and des06, which target site 1, reduced IL‐7 signaling in a dose‐dependent manner, similarly to the anti‐IL‐7Rα antibody lusvertikimab. Surprisingly, des07, which targets site 2, also exhibited an inhibitory effect on IL‐7‐mediated receptor activation (Figure 4D). These three designs exhibited activity at the lowest tested concentration of 1 µg/mL, which corresponds to 49 nm for des03 and des06, and 88 nm for des07.

## 3 Discussion

Protein design has demonstrated tremendous potential in creating new molecules for therapeutic applications, however, sculpting a binding pocket *de novo* remains a major scientific and technological challenge.^[^ ^34^, ^35^ ^]^ This is due to the fact that a protein‐protein binding event occurs under the influence of several interaction forces and major dynamic rearrangements of both the solute and solvent molecules.^[^ ^36^, ^37^ ^]^ The accuracy of estimating energy change associated with these different factors is not uniform using existing methods. Therefore, we resort to a tiered approach whereby simpler binding factors are optimized first. Shape complementarity between the interacting molecular surfaces is one of the major drivers of binding,^[^ ^38^ ^]^ and can be geometrically quantified with high accuracy.^[^ ^39^, ^40^, ^41^, ^42^ ^]^ The relative importance of shape complementarity is particularly magnified in cases of maximum solvent‐exclusion and geometric asymmetry at the binding interface, which can enhance binding affinity and specificity, respectively.^[^ ^43^ ^]^ Here, we explored a complementarity‐first approach, which simplifies the shape‐docking problem through fast and efficient comparisons of surface fingerprints. In contrast to the previously described surface‐centric design approach,^[^ ^12^ ^]^ which identifies structural motifs with both geometric and chemical complementarity to the target site and preserves the sequence of the identified seed, the HECTOR search focuses exclusively on the steric compatibility of potential binding scaffolds. The most compatible scaffolds selected from a structural database then undergo one‐sided interface design, followed by querying the full dynamics of the designed complex for more rigorous ranking. This tiered sampling of geometries, sequences, and dynamics enables a more comprehensive yet computationally tractable exploration of the solution space.

The docking algorithm introduced in this work can identify design templates with locally‐optimal surface topographies from among a large diversity of folds. This overcomes the conventional reliance of design on a small set of binder scaffolds (e.g., immunoglobulins,^[^ ^44^ ^]^ DARPins,^[^ ^45^ ^]^ anticalins,^[^ ^46^ ^]^ affibodies^[^ ^47^ ^]^). Although in this study we used a subset of the X‐ray structures from the PDB, the computational performance of the ultra‐fast docking algorithm allows the screening of much bigger databases. Particularly, databases of structural models predicted by AlphaFold2,^[^ ^48^ ^]^ which are orders of magnitude larger than the PDB, can provide a vast source of potential scaffolds for our design approach.^[^ ^49^, ^50^ ^]^ The scaffold selection stage derisks the development efforts by selecting small, topologically simple, and PTM‐free scaffolds to facilitate production, processing, and quality control.^[1,^ ^51^ ^]^ Our design scheme can be further leveraged to create semi‐humanized binders by restricting the search to structural fragments belonging to the high‐abundance human proteome and carving the novel binding site through minimal mutagenesis. This can substantially mitigate the immunogenic risk associated with therapeutic proteins development, which would be particularly amplified when relying on non‐human scaffolds.

To demonstrate the utility of our computational approach we set out to design binders targeting the receptor‐binding epitope of a growth factor (VEGF). We tested 16 VEGF‐binding designs based on two different template folds, which had low molecular weight, and were mostly well‐expressed, soluble, and stable. Affinity measurements showed a few designs to bind VEGF with nanomolar dissociation constants. Notably, the two templates deployed here mount highly concave epitopes on mostly β‐sheet structures. This has shown particularly advantageous for quenching the convex VEGF epitope, which is likely inaccessible by helical‐scaffolds, hitherto the most‐used scaffolds for *de novo* binders.^[^ ^52^ ^]^ Selected VEGF binders could inhibit the VEGF‐dependent proliferation of leukemia cells, as well as the survival of primary human endothelial cells. The anti‐VEGF activity of one design (Sam0.7) was more potent than an approved anti‐VEGF antibody (bevacizumab) in vitro in a dense 3D microvasculature model, where treatment with Sam0.7 resulted in the dose‐dependent reduction in the area covered by blood vessels and thinning of the capillaries. Finally, we also found these VEGF binders to be effective in reducing the size of tumors in glioma zebrafish xenografts, highlighting their strong therapeutic potential (Figure 3).

As another proof‐of‐concept we used HECTOR pipeline to demonstrate its capacity to target previously untargeted epitopes. Specifically, we targeted two different epitopes on the surface of a cytokine receptor (IL‐7Rα), one overlapping with the ligand binding site, and the other distal from it. For both sites we could successfully identify nanomolar binders upon testing only 8 designed sequences. These designs belonged to three distinct folds, where only one of them was helical. Nonetheless, 6 out of the 8 designs were expressed and stable, and bound IL‐7Rα at sub‐micromolar to sub‐nanomolar affinities. Moreover, several of the designs inhibited IL‐7 signalling in a cell‐based assay at nanomolar levels (Figure 4). These results warrant the further characterization and development of these proteins as anti‐leukemic agents.

The described workflow combines ultra‐fast surface fingerprints for docking, tensorized energy calculations for interface design,^[^ ^30^, ^33^ ^]^ and accelerated molecular dynamics for in silico validation, which marks several advantages over other approaches. Previously described binder design workflows directly or indirectly encode statistical relationships of protein structures and interfaces through deep learning, PDB‐derived rotamers, or knowledge‐based scoring. Conversely, our workflow represents a training‐free approach where surface mapping, sequence design, and MD simulations all rely only on force field parameters, effectively decompressing the latter to derive information on a novel binding interface. Thus, the approach is generalizable for handling any ligand or amino acid chemotype, even when training data are insufficient. Furthermore, the HECTOR fingerprint itself can be adapted to ambivalently evaluate local similarity or local complementarity across large structure databases to unravel networks of putative or designable interactions. This also offers a powerful local, surface‐based alignment method, which is unachievable through existing alignment methods^[^ ^53^, ^54^, ^55^ ^]^ and can be used for function inpainting or regrafting.^[^ ^6^, ^56^, ^57^ ^]^

## 4 Experimental Section

### Selection of Design Scaffolds Using HECTOR Approach

The surface mapping began by constructing a triangulated surface of the entire protein structure using EDTSurf program with a probe radius of 1.4 Å.^[^ ^58^ ^]^ The center of each triangular face was then computed to transition from triangulated mesh to a dot‐surface representation *S_c_* in Cartesian coordinates, yielding an average dot density of 20 dots per Å^2^. The associated vector field describing the outward‐pointing surface normal vector *S_n_* at each surface dot was derived. The position vector and surface normal at each dot would define the local origin and *z*‐axis at each local surface patch, respectively. The protein's dot‐surface was split into a number of overlapping surface patches. Each surface patch $F_{i}=\{s_{c}|s_{c}\;\varepsilon \;S_{c},\frac{∥\left(s_{c}-{s_{c}}_{i}\right)\times {s_{n}}_{i}∥}{∥s_{ni}∥}<R_{max},|\left(s_{c}-{s_{c}}_{i}\right)\cdot {s_{n}}_{i}\left|<\frac{H_{max}}{2}\right\}$ was defined as a set of Cartesian coordinates (*x*, *y*, *z*) of surface dots **s**_*c*_ that lie within a maximum radial distance *R_max_* from the surface normal **s**_*n* *i*_ of the *i* ^th^ dot **s**_*c* *i*_ (i.e. the center of the patch), and within half the maximum axial distance *H_max_* along the surface normal vector $s_{n_{i}}$. These individual surface patches contain their frame of reference where **s**_*c* *i*_ was the origin and **s**_*n* *i*_ was the local *z*‐axis to be projected into a cylindrical coordinate system to yield a forward or inverse 3D map *G_i_* according to Equation 1:

(1) $$T_{p}:f\left(x,y,z\right)\to g\left(r,\theta ,l\right)≔\;\left[\begin{array}{c} \sqrt{x^{2}+y^{2}}\\ 1/\tan\left(\frac{y}{x}\right)\\ k\cdot z \end{array}\right]$$

A projected *G_i_* can be mapped in either a forward (*G*_+*i*_) or an inverse (*G*_−*i*_) direction, depending on the value of $k=\{\begin{array}{c} +1\;forward\\ -1\;inverse \end{array}\;$ to describe a forward‐ or inverse‐mapped surface patch (Figure S1, Supporting Information). At this stage, projected *G*_±*i*_ patches are translation‐invariant, and rotation‐invariant along two principal planes; i.e. the 3D projections are only sensitive to alignments along the θ dimension. Therefore, the indefinite integral across the θ dimension (Equation 2): i) reduces the dimensionality of the 3D projections into compressed 2D density projections, albeit at the loss of some information; ii) renders the 2D maps rotationally‐invariant; and iii) produces maps with constant dimensions.

(2) $$T_{r}:g_{\pm }\left(r,\theta ,l\right)\to h_{\pm }\left(r,l\right)≔\int g_{\pm }\left(r,\theta ,l\right)d\theta$$

Throughout this work, several mapping parameters (Figure S2, Supporting Information) and the complementarity matching algorithm were optimized based on benchmark performance (Table S5, Supporting Information) and memory constraints. The original version of HECTOR was referred to as HECTOR v0.1, and the updated version as HECTOR v0.2.

*HECTOR v0.1*. Surface patches were mapped at a frequency of 1:40 (i.e. mapping the patch around every 40th dot), resulting in ≈0.5 maps per Å^2^. Each map was generated at a density of 1:8 (i.e. taking into account every 8th dot and skipping the other seven). Generated 2D maps covered a radial span of [0, 12] Å from the surface normal axis of the patch, and the axial span of [− 6, 6] Å. The maps were composed of of 30 × 30 bins, where the resolution was 0.4 Å. A bilinear interpolation was applied to the generated maps *h*_±_ (Equation 3) to blur slight deviations in surface‐dot densities, and allow for a degree of conformational insensitivity:

(3) $$T_{r}:h_{\pm }\left(r,l\right)\to j_{\pm }\left(r,l\right)≔\;\sum_{i=0}^{1}\sum_{j=0}^{1}a_{ij}x^{i}y^{j}$$

This yielded the final form of the fingerprint *j*_±_(*r*,*l*) that was directly used for complementarity matching by calculating an R‐factor (Equation 4) that represents the normalized differences between two matrices *F* and *I* ^[^ ^59^ ^]^ describing the forward‐mapped subject patch and the inverse‐mapped query patch, respectively:

(4) $$R=\sum \left|\frac{F}{\sum F}-\frac{I}{\sum I}\right|\;$$

*HECTOR v0.2*. Further upgrades of HECTOR were introduced to improve its specificity and sensitivity, where the upgraded version was used for the presented benchmarks as well as for the design of IL‐7Rα binders. Specifically, the resolution of maps was increased to 0.2 Å, with Cartesian mapping of a radial span of [0, 10] Å and an axial span of [− 10, 10] Å, resulting in larger maps with dimensions of 50 × 100 bins. The mapping frequency was also increased to 1:5 (i.e. mapping the patch around every 5th dot), without any dot skipping during map generation (i.e. maximum mapping density). On average, this mapping frequency resulted in 4 maps per Å^2^. All maps underwent sigmoidal radial fading with a fade radius of 40 bins and a slope of 0.3 to reduce the contribution of edge dots. Given the increased mapping density, the maps were sufficiently smooth and the bilinear interpolation step was eliminated. Additionally, protein dot surfaces were inflated by 0.5 Å before mapping to account for uncertainty in the molecular surface. The R‐factor formula was also improved (Equation 5) to better emphasize the overlap between forward subject map *(F)* and inverse query map *(I)*:

(5) $$R=\frac{\sum \left(\frac{2\times F\times I+C}{F^{2}+I^{2}+C}\right)}{N}$$

where *N* was the number of elements in matrices *F* and *I*; *C* = 0.0001 was a constant introduced for numerical stability.

### HECTOR Benchmarking

HECTOR benchmarking (Figure 1B,D; Figure S17, Supporting Information) was performed on a set of 7 split proteins. One fragment was used as a query protein, while the second was used as a subject protein (Table S3, Supporting Information). For each protein, 10 query maps were selected at the fragments interface (Figure S16, Supporting Information). Pairwise combinations of these 10 query maps (45 pairs) were used as input. A pair of query maps was compared against all subject maps as follows. First, all subject maps were ranked by R‐factor to the first query map and the top 5% of subject maps with the lowest R‐factors were selected. Next, the same procedure was performed for the second query map. Subsequently, subject pairs were formed from two groups of selected subject maps. These subject pairs were filtered based on their inter‐patch distance *d*, which had to be within a range of ± 0.01 Å from the distance between two query patches. Selected pairs of subject patches were aligned to the query pair using the Kabsch algorithm,^[^ ^60^ ^]^ where the points for alignment were the centers of the two query/subject patches and the endpoints of their normals. Finally, a pair score was calculated for each subject pair as (3 × *R_avrg_* + *RMSD*), where *R_avrg_* was the average R‐factor for two query maps, and RMSD was the root mean squared deviation between two paired sets of points. Subject pairs were ranked by the pair score. To analyze the results, we evaluated how many true positive subject pairs were found within the top 100 hits and how well they were ranked. A subject pair was counted as a true positive if the coordinates of both subject maps were within 3 Å of the coordinates of the corresponding query maps. The enrichment factor was calculated as:

(6) $$\begin{array}{cc} & \frac{N\;correct\;subject\;pairs\;identified\;in\;top\;100}{100}/\\ & \;\frac{N\;possible\;solutions}{Total\;N\;of\;subject\;maps\;pairs\;with\;inter-patch\;distance\;d} \end{array}$$

Final HECTOR parameters were additionally tested on a set of natural protein‐protein complexes (PDB ID: 1BVN, 1CGI, 1I2M, 1JTG, 1KXP, 1UDI, 1YVB) selected from^[^ ^61^ ^]^ based on their “gap distance” scores (Figure S17, Supporting Information).

Performance benchmarks report the wall‐clock time of computing the R‐factor for two (or more, as indicated) query maps against all maps from nine randomly selected proteins, with an average of 33982 maps per protein, each map of dimensions of 50 × 100 (with FP32 precision). The CPU performance was measured on an Intel Core i9‐14900K, while the GPU performance was measured on an Nvidia GeForce RTX 4090 using the CUDA backend of ArrayFire.^[^ ^62^ ^]^

### Computational Design of the VEGF Binders

Two proximal VEGF surface patches (*q_1_* and *q_2_* separated by distance *d_q_*) around residues I46 and H86 (PDB: 4KZN) at the receptor‐binding site were used as query in a two‐vs‐all search, which was bounded by a distance restraint with a tolerance of ±3 Å. The tiered search algorithm followed three consecutive steps: 1) the first query patch was compared against all subject patches (one‐vs‐all); 2) lowest 50 R‐factor subject patches matching q_1_ were searched within a distance band *d_q_* ± tolerance for secondary hits matching *q_2_*; 3) the lowest R‐factor query: subject pair was identified. Retrieved top six HECTOR hits featuring the identified subject patches (PDB: 1C7N, 1EYN, 1F73, 1KCD, 1OH0, 1PM1) were docked against VEGF based on their surface complementarity using PatchDock.^[^ ^22^ ^]^ For that, the residues corresponding to patches with low R‐factor in both the hit and VEGF were set as binding residues and used as constraints for rigid body docking using default parameters, as set by the buildParamsToOutfile.pl PatchDock utility (Supporting Information). For each job, the top few poses were selected and further refined by using short, low‐temperature molecular dynamics simulations. These simulations were run with a generalized Born implicit solvent, where the temperature was set to 250 K using a Langevin thermostat through the NAMD2 software.^[^ ^63^ ^]^

For the one‐sided interface design stage, a design protocol was implemented in RosettaScripts^[^ ^23^ ^]^ that deploys multiple design movers interlaced with backbone movers. The movers were bundled within two main iterative generic Monte Carlo sampling stages, which were implemented with interface ΔΔG and packstat^[^ ^64^ ^]^ filters for scoring. This design protocol was run for 100 instances per input decoy, and the entire procedure was performed in 4–6 successive design rounds (Supporting Information). Designs with the interface ddG higher than ‐30 REU or with the RosettaHoles score lower than 0.65 were discarded. The top few thousand decoys were clustered to unique sequences and were further filtered using two‐stage molecular dynamics ranking. The first stage filtered the designs using serial tempering simulations in implicit solvent between cool and hot basins of 250 and 370 K for 6 and 8 ps, respectively. This was conducted for a total of 60 cycles separated by 100 steps of conjugate gradient minimization per simulation (which was roughly equivalent to 1.2 ns of sampling time), for 3 replicas per decoy (Supporting Information). The conformational stability across the cycles and replicas was quantified for each decoy as a function of the RMSD from the design using a selection of interface residues.^[^ ^65^ ^]^ This reduced the decoys to a few hundred candidates that were further filtered by a second stage of steered molecular dynamics in an explicit solvent under constant pressure and temperature. In the latter stage, the binder's backbone atoms were fixed using harmonic restraints along the z‐dimension, and the binding target was pulled along the z‐dimension at a fixed velocity of 2 Å/ns using a force constant of 50 kcal/mol∙Å, for the simulation duration of 7.5 ns (Supporting Information). The estimated free energy of unbinding (*W*) was evaluated as $W_{t_{o}\;tot_{e}}=\int_{t_{o}}^{t_{e}}v\left(t\right)F\left(t\right)dt\;$where *F*(*t*) and *v*(*t*) are the pulling force and velocity vectors over the simulation time span (i.e. $t_{0}\to t_{e}$), respectively.^[^ ^66^, ^67^ ^]^ Example trajectories for both simulation types, along with their corresponding analysis scripts, are available in the Zenodo repository (https://doi.org/10.5281/zenodo.14028991).

Figure S4 (Supporting Information) illustrates the designed binding interface of Sam0.7, selected as the best‐performing design based on experimental validation.

### Bacterial Protein Expression and Purification of VEGF Binders

Synthetic genes encoding the human VEGF165 (NP_001165097, Gly34‐Asp135) and the designs were cloned into the pET28a(+) expression vector between the *NdeI* and *XhoI* cloning sites in‐frame with a thrombin cleavage site and an N‐terminal poly‐histidine purification tag (General Biosystems, Inc.; Synbio Technologies, Inc.). Plasmids were transformed into chemically competent *E. coli* BL21(DE3) using the heat shock method. Transformed cells were grown in LB medium supplemented with 40 µg mL^−1^ kanamycin at 37 °C. At OD600 of 0.6–1.0, cells were induced with 1mM IPTG and incubated overnight at 25 °C for protein expression. For purification of all Sam designs and Sima3.2, cells were harvested by centrifugation at 5000 g at 4 °C for 20 min and lysed in 25 mL of lysis buffer (1m guanidinium chloride, 100 mm NaCl, 50mm Tris‐HCl pH 8.0) supplemented with a tablet of the cOmplete, EDTA‐free Protease Inhibitor Cocktail (Roche, 5056489001) and 3 mg of lyophilized DNase I (PanReac AppliChem, A3778) using a Branson Sonifier S‐250 (Fisher Scientific). The lysate was cleared by centrifugation at 28000 g for 50 min, and the supernatant was passed through a 0.45 µm filter (Millipore, SLHV033RS). The sample was applied to a 5 mL HisTrap HP column (Cytiva, GE17‐5248‐01). The running buffer was 150 mm NaCl, 30 mm Tris‐HCl pH 8.0. After sequential washing the column with 30 mL of the running buffer supplemented with 0 or 50 mm imidazole, fractions were collected by linear gradient elution using 150 mm NaCl, 30 mm Tris‐HCl pH 8.0, 500 mm imidazole buffer. In case of other Sima designs and human VEGF, proteins were extracted from the insoluble fraction of the cell pellet by stirring in a phosphate‐buffered saline (PBS) with 5 m guanidinium chloride, and 25 mm DTT for 2 h at room temperature. The mixture was gradually diluted 5 times with PBS and cleared by centrifugation at 28000 g for 50 min. The proteins were purified from the filtered supernatants by immobilized metal‐affinity chromatography (IMAC) as described above. The only difference was that the composition of the running buffer was 150 mm NaCl, 30 mm Tris‐HCl pH 8.0, 1 m urea, 1.25 mm reduced glutathione, 0.25 mm oxidized glutathione. The eluted fractions containing the protein of interest were pooled, concentrated using 10 kDa MWCO centrifugal filters (Millipore, UFC901024), and further purified on a Superdex Increase 75 10/300 gel filtration column (Cytiva, 29148721) using PBS. Gel filtration fractions containing pure protein in the desired oligomeric state (monomeric fraction of Sam0.7, dimeric fraction of Sima3.2) were pooled, concentrated, and stored at −20 °C for subsequent analyses. Both IMAC and gel filtration steps were performed on an Äkta Pure chromatography system (Cytiva).

### Thermostability Analysis of VEGF Binders

Nanoscale differential scanning fluorimetry (nanoDSF) using Prometheus NT.48 (Nanotemper) was applied to evaluate the thermostability of the designs. Capillaries (Nanotemper, PR‐C002) were filled with 1 mg mL^−1^ protein samples in three replicates. A melting scan was performed across the temperature range from 20 °C to 90 °C with a temperature ramp of 1 °C min^−1^. In addition to measuring the intrinsic fluorescence intensity ratio (350/330 nm), light intensity loss due to scattering (back reflection) was measured to detect protein aggregation.

### Isothermal Titration Calorimetry (ITC)

To determine whether Sam designs bind to VEGF, ITC experiments were performed using MicroCal PEAQ‐ITC (Malvern Panalytical). All samples were prepared in PBS. The cell was loaded with VEGF solution at concentrations from 80 to 500 µm for different measurements. The syringe was loaded with ≈400 µm of Sam designs (375 µm of Sam0.2, 416 µm of Sam0.7). All measurements were carried out with the following parameters: 18 injections of 2 µL with 150 s between injections, reference power 41.9 µW, stirring speed 750 rpm, temperature 25 °C. Experimental data were fitted using MicroCal PEAQ‐ITC Analysis Software. *K_d_* values were corrected taking into account N‐value that describes active fraction of protein in the cell and in the syringe.

### Microscale thermophoresis (MST)

To test ability of Sima designs to bind VEGF, MST measurements were performed on Monolith NT.115 (Nanotemper). Sima1.1, Sima3.1, and Sima3.2 were labeled with fluorescent dye using Protein Labeling Kit RED‐NHS 2nd Generation (Nanotemper, MO‐L011). For each experiment 16 samples were prepared with constant concentration of labeled Sima protein (100 nm of Sima1.1, 40 nm of Sima3.1, and 80 nm of Sima3.2) and decreasing concentrations of VEGF (2‐fold dilutions from 12.5 µm to 0.4 nm for Sima1.1 measurements, from 64 µm to 2 nm for Sima3.1 measurements, and from 2 µm to 61 pm for Sima3.2 measurements). All dilutions were made in PBS supplemented with 0.1% PluronicF‐127 (Nanotemper). Measurements were done in Monolith NT.115 Premium capillaries (Nanotemper, MO‐K025) at 25 °C. Dissociation constants (*K_d_*) were derived by fitting normalized fluorescence values (cold region: −2–−1 s, hot region: 4–6 s) against ligand concentrations.

### SPR Binding Assay for VEGF Binders

Multi‐cycle kinetics experiments were performed on a Biacore X100 system (GE Healthcare Life Sciences). Recombinant Human VEGF165 (rhVEGF) (R&D Systems, 293‐VE‐010/CF) was diluted to 50 µg mL^−1^ in 10 mm acetate buffer pH 5.0 and immobilized on the surface of a CM5 sensor chip (Cytiva, 29149604) using standard amine coupling chemistry. The designs were diluted in the running buffer (PBS with 0.05% v/v Tween‐20). Analyses were conducted at 25 °C at a flow rate of 10 µL min^−1^. Five increasing concentrations of the sample solution (111, 134, 161, 193, 231 nm for Sam0.7; 44, 66, 100, 148, 222 nm for Sima3.2) were injected over the functionalized sensor chip surface for 60 s, followed by a 60 s dissociation with running buffer. At the end of each run, the sensor surface was regenerated with a 30 s injection of 50 mm NaOH at a flow rate of 10 µL min^−1^. The reference responses and zero‐concentration sensograms were subtracted from each dataset (double‐referencing). Association rate (*k_a_*), dissociation rate (*k_d_*), and equilibrium dissociation (*K_d_*) constants were obtained using the linearization method described in.^[^ ^6^ ^]^

For competition assay, Sam0.7 was diluted to 100 µg mL^−1^ in 10 mm acetate buffer pH 5.0 and immobilized on the surface of a CM5 sensor chip (Cytiva, 29149604) using standard amine coupling chemistry. 500 nm VEGF (BioLegend, 583706), 100 nm bevacizumab (MedChemExpress, HY‐P9906), or a mixture of 500 nm VEGF and 100 nm bevazicumab preincubated for 30 min at room temperature were injected over the functionalized sensor chip surface for 100 s, followed by a 100 s dissociation with running buffer (PBS with 0.05% v/v Tween‐20). Runs were conducted at 25 °C at a flow rate of 30 µL min^−1^. At the end of each run, the sensor surface was regenerated with a 30 s injection of 10 mM glycine pH 2.5 at a flow rate of 10 µL min^−1^.

### X‐ray Structure Determination of Sam Designs

Crystallization screens were set up with a Mosquito robot (TTP Labtech) in 96‐well plates at 21 °C, using 75 µL of reservoir solution and sitting drops containing 400 nL of reservoir and 400 nL of protein solution of either Sam0.2 or Sam0.7 at a concentration of 8 mg mL^−1^. Within 7–14 days, crystals of Sam0.2 and Sam0.7 grew in a condition containing 20% (w/v) PEG 3350 and 0.2 m KSCN. Crystals were cryoprotected through the addition of 15% PEG 400, flash cooled and stored in liquid nitrogen until data collection. Diffraction data were collected at 100K on an EIGER2 16M detector at beamline X10SA at the Swiss Light Source (SLS). Data were processed and scaled using XDS^[^ ^68^ ^]^ and the structures solved using molecular replacement with MOLREP^[^ ^69^ ^]^ and the computational models of Sam0.2 and Sam0.7 as search models. Structures were completed by cyclic refinement with REFMAC5^[^ ^70^ ^]^ and modeling using Coot.^[^ ^71^ ^]^ Data collection and refinement statistics are summarized together with PDB accession codes in Table S3 (Supporting Information).

### HUVEC Survival Assay

HUVECs (Sigma, 200–05N) were cultured in Endothelial Cell Growth Medium (EGM) (Cell Applications, Inc., 211–500) and used at passages 3 to 6. 300 µL of cell suspension were seeded in a 48‐well plate (Nunc, 150687) at a density of 10^5^ cells mL^−1^. When the culture reached ≈80% confluency, cells were washed once with DPBS, and medium was changed to Endothelial Cell Basal Medium (EBM) (Cell Applications, Inc., 210–500) or EBM supplemented with 30 ng mL^−1^ rhVEGF, or with 30 ng mL^−1^ rhVEGF and different concentrations of VEGF inhibitors varying from 1 µg/mL to 100 µg/mL. Before the experiment, inhibitors were preincubated with rhVEGF for 30 min at room temperature. After incubation for 48 h at 37 °C, 5% CO_2_, 60 µL of CellTiter‐Blue Reagent (Promega, G8080) were added to the wells, and the plate was incubated for an additional 1 h under the same conditions. Cell survival was monitored by measuring fluorescence (560/590 nm) using a Synergy HTX Microplate Reader (BioTek). The results were normalized to the average of fluorescence values from the wells with EBM only. The experiment was repeated three times independently, with three technical replicates for each trial.

### U937/HEK293T Cell Proliferation Endpoint Analysis

U937 cells (DSMZ, ACC 5) were cultured in RPMI 1640 medium (Gibco, 22400071) supplemented with 10% FBS (Gibco, 10082147). HEK293T cells (DSMZ, ACC 635) were cultured in DMEM medium (Gibco, 41966029) supplemented with 10% FBS (Gibco, 10082147). Cells were pelleted by centrifugation at 300 g for 5 min, washed once with DPBS (Gibco, 14190144), and once with non‐supplemented medium. After the last washing step, cells were resuspended in RPMI 1640 (for U937) or DMEM (for HEK293T) medium supplemented with 1% FBS. 100 µL of U937 cell suspension were seeded in a 96‐well plate (Sarstedt, 83.3925.500) at a density of 2 × 10^5^ cells mL^−1^. For HEK293T, 100 µL of cell suspension were seeded in a 96‐well plate (Corning, 3596) at a density of 5 × 10^4^ cells mL^−1^. Different concentrations of Sam0.7 or Sima3.2 varying from 1 to 100 µg mL^−1^ were added to the wells in triplicates. DPBS was added to the wells serving as an untreated control. After incubation for 72 h at 37 °C, 5% CO_2_, 20 µL of CellTiter‐Blue Reagent (Promega, G8080) were added to the wells, and the plate was incubated for an additional 1 h under the same conditions to allow cells to convert resazurin to resorufin. Cell viability was monitored by measuring fluorescence (560/590 nm) using a Synergy HTX Microplate Reader (BioTek). The average of fluorescence values of the culture medium background were subtracted from all fluorescence values of the experimental wells. The data were presented as a percentage of untreated control fluorescence values. U937 experiment was repeated three times independently, with three technical replicates for each trial.

### In Vitro 3D Microvasculature Analysis

A human episomal iPSC line from a healthy female donor, E1 (ThermoFisher Scientific, A18945) was cultured on Geltrex‐coated (Gibco, A1413302) plates in Essential 8 Medium (Gibco, A1517001). Endothelial cells (ECs) and pericytes (PCs) were differentiated following established protocols.^[^ ^72^, ^73^ ^]^ ECs were labeled with the pJG‐IRBP‐mCherry viral vector to enable live cell imaging. Differentiated ECs were cultured on 0.2% gelatin‐coated plates (Sigma–Aldrich, G2500) in Endothelial Cell Growth Medium (PromoCell, C‐22020) supplemented with 30 ng mL^−1^ vascular endothelial growth factor A (PeproTech, 100–20) and 20 ng mL^−1^ fibroblast growth factor 2 (PeproTech, 100–18B); PCs were cultured in Essential 6 Medium (Gibco, A1516501) supplemented with 10% FBS. Confluent ECs and PCs were detached with Accumax (Sigma‐Aldrich, A7098) pelleted by centrifugation at 300 g for 3 min and resuspended in a fibrin gel composed of 5 mg mL^−1^ fibrinogen (Sigma‐Aldrich, F6755) and 4 U mL^−1^ thrombin (Sigma–Aldrich, T9549), with a final cell concentration 3 × 10^6^ cell mL^−1^ of each cell type. 3D fibrin drops were formed using 5 µL of cell suspension in 96‐well plates (Falcon, 351172) and cultured in Endothelial Cell Growth Medium supplemented with 20 ng mL^−1^ fibroblast growth factor 2. Two concentrations of Sam0.7 (50 and 100 µg mL^−1^) were added to the wells, with 5 repeats for each condition; Sam_ctrl protein was added to the wells as control. The microvasculature was grown for 7 days and then imaged with an Axio Observer Z1/7 (Zeiss) microscope. Fiji/ImageJ software was utilized to analyze the microvasculature parameters. The statistical analysis was done using GraphPad Prism 10 software. All procedures were in accordance with the Helsinki Convention and approved by the Ethical Committee of the Eberhard Karls University Tübingen (no. 396/2021BO2).

### Xenotransplantation of U937‐GFP or LNZ308‐GFP Cells in Zebrafish Embryos

Zebrafish lines were maintained according to standard protocols and handled in accordance with European Union animal protection directive 2010/63/EU and approved by the local government (Tierschutzgesetz §11, Abs. 1, Nr. 1, husbandry permit 35/9185.46/Uni TÜ). The role of the designed inhibitors in cell survival and proliferation was evaluated by xenotransplantation of U937‐GFP and LNZ308‐GFP cell lines in zebrafish embryos at 1.5 dpf. U937‐GFP cells were generated by lentiviral transduction with a lentiviral construct expressing GFP, pRRL.PPT.SF.i2GFPpre, kindly provided by A. Schambach, Hannover Medical School. LNZ308‐GFP cells were generated by lentiviral transduction with a third generation GFP‐expressing lentivirus based on the lentiviral vector pLJM1‐EGFP (Plasmid #19319, Addgene). 1 nL of U937‐GFP cell suspension was injected at a density of 2 × 10^5^ cells µL^−1^ (≈200 cells) into the perivitelline space, whereas LNZ308‐GFP glioma cells (1 nL, ≈200 cells) were orthotopically implanted into the brain of anesthetized embryos at 29 hpf. The embryos were incubated at 35 °C after transplantation. After 4–5 h alive embryos with good engraftment were injected with the same volume (4 nL) of either control (PBS or 3.0 mg mL^−1^ of inactive protein mvn_cntrl^[^ ^6^ ^]^ as a negative control, or 25 mg mL^−1^ of Bevacizumab as a positive control) or inhibitors (3.5 mg mL^−1^ of Sam0.7, and 2.4 mg mL^−1^ of Sima3.2). Injections were done either in the brain or bloodstream of zebrafish embryos transplanted with LNZ308‐GFP cells or U937‐GFP cells, respectively. The embryos were positioned and orientated laterally within cavities formed in 1% agarose on a 96‐well plate for imaging. To quantify the level of transplanted human cells, zebrafish embryos were imaged by a Nikon fluorescent stereomicroscope (SMZ18). All images taken under the same condition were analyzed with Imaris software. The fluorescently labeled cells were quantified by surface measurement option with background subtraction. These values were calculated in each embryo of each group and plotted using Grafpad. The statistical analysis was performed using Prism 7 software.

### Computational Design of the IL‐7Rα Binders

Binders were designed against two distinct IL‐7Rα epitopes (site 1 and site 2). At each site, three query surface patches corresponding to specified residues (PDB: 3DI3) were selected: L80, K138, Y192 for site 1; V28, L68, P202 for site 2. HECTOR search was performed in a two‐vs‐all fashion, where all unique pairwise combinations of three query patches were compared against all subject patches (i.e. maps). First, the top 5% of subject maps with the lowest R‐factor relative to the first query were selected. Second, the top 5% of subject maps with the lowest R‐factor relative to the second query were selected. Next, inter‐patch distances for subject maps from these two selected groups were calculated. Only pairs with an inter‐patch distance within a tolerance of ±0.01 Å from the distance between the two query patches were retained. After this, the average R‐factor for the two query maps was calculated for each subject pair, and only pairs with an average R‐factor lower than −0.82 were retained. Selected subject pairs were aligned to the query pairs using the Kabsch algorithm,^[^ ^60^ ^]^ where the points for alignment were the centers of the two query/subject patches and the endpoints of their normals. Subject pairs with RMSD less than 0.5 Å were retained. Additionally, after alignment, scaffolds with fewer than 25 interfacial residues or strong steric clashes were discarded. The steric overlaps were calculated using the Damietta's voxelized projections of solvation kernels, where docked poses with overlaps of more than 125000 voxels were filtered out.^[^ ^30^ ^]^ In total, after all filtering steps, 39 hits remained for site1 and 19 hits for site 2. Filtered scaffolds were sorted by RMSD, and the top 5 scaffolds were selected. These were 5NLC, 6B8F, 4F2N, 5ZGT, 7O69 for site 1, and 6YUD, 5CIV, 4D7P, 7P76, 4K4B for site 2. Proteins that had not been previously expressed in *E. coli* were excluded from the shortlist. Scaffold selection was followed by interface design using the Damietta design software.^[^ ^30^, ^33^ ^]^ After the first design round, all generated mutants were filtered using temperature‐accelerated molecular dynamics routines as described for anti‐VEGF designs, and the most stable 5 models belonging to 5NLC and 6B8F scaffolds for site 1, and to 6YUD scaffold for site 2 were selected. These designs were MD‐minimized before a second round of Damietta design. Damietta spec files are shown in Supporting Information. Finally, 8 candidates were selected for experimental characterization based on MD rescoring: 6 designs for site 1 (des01, des02 based on 5NLC scaffold; des03‐des06 based on 6B8F scaffold) and 2 designs for site 2 (des07, des08 based on 6YUD scaffold). Figure S4 (Supporting Information) illustrates the designed binding interface of des03, selected as the best‐performing design based on experimental validation.

The above steps have varying computational loads, where the most expensive step is mapping the surfaces of the entire database of structures. While this step consumes 15 ms per surface patch, it takes on average 7 min per protein domain on CPU, which is performed once for a given database. Conversely, HECTOR complementarity matching consumes only 20 µs per pairwise evaluation on CPU. For interface design, Damietta simulations were run to a limit of 5000 CPU‐hours per scaffold. The accelerated MD simulations, which are used to filter several hundred candidates, typically consume several minutes per replica per candidate on a GPU‐accelerated setup.

### Bacterial Protein Expression and Purification of IL‐7Rα Binders

Synthetic genes encoding the unmodified templates and designs were cloned into the pET28a(+) expression vector between the *NdeI* and *XhoI* cloning sites, in‐frame with a thrombin cleavage site and an N‐terminal poly‐histidine purification tag (GenScript BioTech, Netherlands B.V.). Plasmids were transformed into chemically competent *E. coli* BL21(DE3) cells using the heat shock method. Transformed cells were grown in LB medium supplemented with 40 µg mL^−1^ kanamycin at 37 °C. At OD600 of 0.7–1.0, cells were induced with 1 mm IPTG and incubated overnight at 20 °C for protein expression. Cells were harvested by centrifugation at 6000 g at 8 °C for 10 min and lysed in 30 mL of lysis buffer (1.5 m urea, 500 mm NaCl, 20 mm Tris‐HCl pH 8.0) supplemented with a cOmplete, EDTA‐free Protease Inhibitor Cocktail tablet (Roche, 5056489001) and 3 mg of lyophilized DNase I (PanReac AppliChem, A3778), using a Branson Sonifier S‐250 (Fisher Scientific) or a French pressure cell press (SLM Instruments, KIN020). The lysates were cleared by centrifugation at 35000 g for 30 min. Proteins were purified from either the supernatants or the pellets. Supernatants were passed through a 0.45 µm filter (Millipore, SLHV033RS), while pellets were resuspended in solubilizing buffer #1 (8 m urea, 500 mm NaCl, 20 mm Tris‐HCl pH 8.0) or solubilizing buffer #2 (0.2% N‐Lauroylsarcosine (NLS), 1 mm PMSF, 40 mm Tris‐HCl pH 8.0). For purification of the 6YUD template and the corresponding designs (des07 and des08) and the 5NLC template, soluble fractions of lysates were applied to a 5 mL HisTrap HP column (Cytiva, GE17‐5248‐01) for Nickel IMAC. The running buffer was 500 mm NaCl, 25 mm imidazole, 20 mm Tris‐HCl pH 8.0. After sequentially washing the column with 15 column volumes (CV) of running buffer supplemented with 50 mm imidazole, fractions were collected by linear gradient elution using a buffer containing 500 mNaCl, 20 mm Tris‐HCl pH 8.0, and 500 mm imidazole. des01, des02, and des05 were extracted from the insoluble fraction of the cell pellet by stirring in solubilizing buffer #1 for 4 h at 8 °C. For purification of des03, des04, des06, and the 6B8F template, proteins were extracted from inclusion bodies by stirring in solubilizing buffer #2 overnight at room temperature. For designs extracted with urea, the solubilized mixture was diluted twofold with running buffer and cleared by centrifugation at 35000 g for 20 min. Proteins were then purified from the cleared supernatants by IMAC as described above. The running buffer composition was modified by adding urea: 500 mm NaCl, 20 mm Tris‐HCl pH 8.0, 1.5 m urea, 25 mm imidazole. For designs extracted using NLS, the only difference was in the running buffer composition, which contained 500 mm NaCl, 20 mm Tris‐HCl pH 8.0, 0.2% NLS, 25 mM imidazole. The eluted fractions containing the protein of interest were pooled, concentrated using 10 kDa MWCO centrifugal filters (Millipore, UFC901024), and further purified on a Superdex75 26/60 gel filtration column (Cytiva, 28‐9893‐34) for des01, des02, and des07; a Superdex75 16/60 gel filtration column (Cytiva, 28‐9893‐33) for des08 and 6YUD; a Superdex200 16/60 gel filtration column (Cytiva, 28‐9893‐35) for des05; a Superdex200 Increase 10/300 gel filtration column (Cytiva, 28990944) for des03, des04, des06, and 6B8F; and a Superdex75 Increase 10/300 gel filtration column (Cytiva, 29148721) for design 5NLC. The running buffer was 20 mm HEPES pH 7.4, 150 mm NaCl, 1.5 m urea for des01, des02, des07, des08, 5NLC, des07, des08, and 6YUD; or 10 mm HEPES pH 7.4, 150 mm NaCl, 0.5 mm EDTA, 0.05% Tween‐20 for des03, des04, des06, and 6B8F. Gel filtration fractions containing pure protein in the desired oligomeric state (monomeric for 5NLC, des01, and des02, des08; dimeric for 6YUD, and des07; 24‐mer for 6B8F, des03, des04, des05, and des06) were pooled, concentrated, snap‐frozen in liquid nitrogen, and stored at −20 °C for subsequent analyses. Both IMAC and gel filtration steps were performed on an Äkta Pure chromatography system (Cytiva).

### Thermostability Analysis of IL‐7Rα Binders

Nanoscale differential scanning fluorimetry (nanoDSF) using Prometheus NT.48 (Nanotemper) was applied to evaluate thermostability of the designs. Capillaries (Nanotemper, PR‐C002) were filled with 0.5 mg mL^−1^ protein samples in three replicates. Melting scan was performed across the temperature range from 20 °C to 110 °C with a temperature ramp of 1 °C min^−1^.

### SPR Binding Assay for IL‐7Rα Binder

Multi‐cycle kinetics experiments were performed on a Biacore X100 system (GE Healthcare Life Sciences). Biotinylated Recombinant Human IL‐7R alpha/CD127 Fc Chimera Avi‐tag (IL‐7Rα‐Fc‐Avi) (R&D Systems, AVI10317/CF) was diluted to 100 µg mL^−1^ in SPR running buffer HBS‐EP+ (10 mM HEPES pH 7.4, 150 mM NaCl, 3 mM EDTA, 0.05% Tween‐20) and immobilized on the surface of an SA sensor chip (Cytiva, BR100398) using biotin‐streptavidin coupling chemistry. The designs were diluted in the running buffer (HBS‐EP+). Analyses were conducted at 25 °C at a flow rate of 30 µL min^−1^. The increasing concentrations of sample solution (for IL‐7: 26, 40, 59, 89, 133 nm; for 5NLC: 195, 290, 440, 660, 990 nm; for 6YUD: 38, 75, 150, 300 nm; for 6B8F: 40, 59, 89, 133, 200, 300 nm; for des01: 123, 159, 207, 269, 350 nm; for des03: 159, 207, 269, 350, 455 nm; for des04: 269, 350, 455, 592, 769, 1000 nm; for des06: 123, 159, 207, 269, 350 nm; for des07: 269, 350, 592, 769, 1000 nm; for des08: 59, 132, 296, 444, 667 nm) were injected over the functionalized sensor chip surface for 60 s, followed by a 120 s dissociation with running buffer. At the end of each run, the sensor surface was regenerated with a 60 s injection of 10 mm glycine‐HCl pH 1.5 at a flow rate of 10 µL min^−1^, followed by a 30 s injection of 4 m MgCl₂ at a flow rate of 10 µL min^−1^. The reference responses and zero‐concentration sensograms were subtracted from each dataset (double‐referencing). Association rate (*k_a_*), dissociation rate (*k_d_*), and equilibrium dissociation (*K_d_*) constants were obtained using the linearization method described in.^[^ ^6^ ^]^

### IL‐7 Signaling Inhibition Assay

HEK‐Blue IL‐7 cells (Invivogen, hkb‐il7) were cultured in growth medium: DMEM, 4.5 g/L glucose, 2 mm L‐glutamine, 10% heat‐inactivated FBS, penicillin‐streptomycin (100 U mL^−1^–100 µg mL^−1^), 100 µg mL^−1^ normocin. One passage prior to the assay cells were cultured in growth medium without normocin (test medium). 90 µL of cell suspension in test medium were seeded in a 96‐well plate (Corning, 3596) at a density of 5.6 × 10^5^ cells mL^−1^. 10 µL of DPBS (negative control), lusvertikimab (positive control, final concentration 25 µg mL^−1^), des01, des03, des06, or des07 (final concentrations 1, 10, 25 µg mL^−1^) were added to the wells. After incubation for 1 h, human IL‐7 (R&D Systems, 207‐IL‐005/CF) was added to the wells at the concentration of 0.3 ng mL^−1^. After 8 h incubation at 37 °C, 5% CO_2_, 20 µL of cells supernatant were added to a new 96‐well plate and mixed with 180 µL of resuspended QUANTI‐Blue Solution (Invivogen, rep‐qbs). Plate was incubated 2 h at 37 °C. SEAP levels were determined by measuring absorbance at 630 nm using a Synergy HTX Microplate Reader (BioTek). The experiment was repeated three times independently, with three technical replicates for each trial. The data were presented as absorbance normalized to untreated control (without IL‐7).

## Conflict of Interest

The authors declare no conflict of interest.

## Author Contributions

Conceptualization was carried out by M.E., A.L., P.M., J.S., and J.P. Development of the HECTOR software and design of constructs were performed by M.E., K.M., and M.P. Biophysical characterization was performed by K.M., M.E., V.H., and M.C., while cell‐based experiments were conducted by K.M., G.T., N.B.B., P.B., and J.F. Crystallographic studies were carried out by K.M. and M.D.H. In vitro microvasculature experiments were performed by N.P., M.V., and S.L., and zebrafish experiments were conducted by N.A. Funding acquisition was the responsibility of A.L., P.M., J.S., and M.E. Resources were provided by A.L., P.M., and J.S. Supervision was undertaken by A.L., M.E., J.S., and P.M. The original draft of the manuscript was written by K.M., M.E., M.C., N.A., and N.P., with all authors contributing to the review and editing of the final manuscript.

## Supporting information



Supporting Information

## Data Availability

Coordinates and structure factors of Sam0.2 and Sam0.7 were deposited in the PDB under the accession codes 8BL5 and 8BL9, respectively. HECTOR software is publicly available at the CodeOcean repository (https://doi.org/10.24433/CO.9243108.v1).
