# Supplementary material for: A Complementarity‐Based Approach to De Novo Binder Design
Source: Adv Sci (Weinh). 2025 Jul 21;12(33):e02015. doi: 10.1002/advs.202502015 (PMC12412469; doi:10.1002/advs.202502015)
Supplement: Supplementary file 1 — Supporting Information [file ADVS-12-e02015-s001.pdf]

ADVANCED SCIENCE
Open Access

Supporting Information

for *Adv. Sci.*, DOI 10.1002/advs.202502015

A Complementarity-Based Approach to *De Novo* Binder Design

Kateryna Maksymenko, Valeriia Hatskovska, Murray Coles, Narges Aghaallaei, Natalia Pashkovskaia, Natalia Borbarán-Bravo, Matteo Pilz, Philip Bucher, Mareike Volz, Joana Pereira, Marcus D. Hartmann, Ghazaleh Tabatabai, Judith Feucht, Stefan Liebau, Patrick Müller, Andrei N. Lupas, Julia Skokowa and Mohammad ElGamacy**

Supporting Information

A Complementarity-Based Approach to *De Novo* Binder Design

Kateryna Maksymenko, Valeriia Hatskovska, Murray Coles, Narges Aghaallaei, Natalia Pashkovskaia, Natalia Borbarán-Bravo, Matteo Pilz, Philip Bucher, Mareike Volz, Joana Pereira, Marcus D. Hartmann, Ghazaleh Tabatabai, Judith Feucht, Stefan Liebau, Patrick Müller, Andrei N. Lupas, Julia Skokowa, Mohammad ElGamacy**

Figure S1

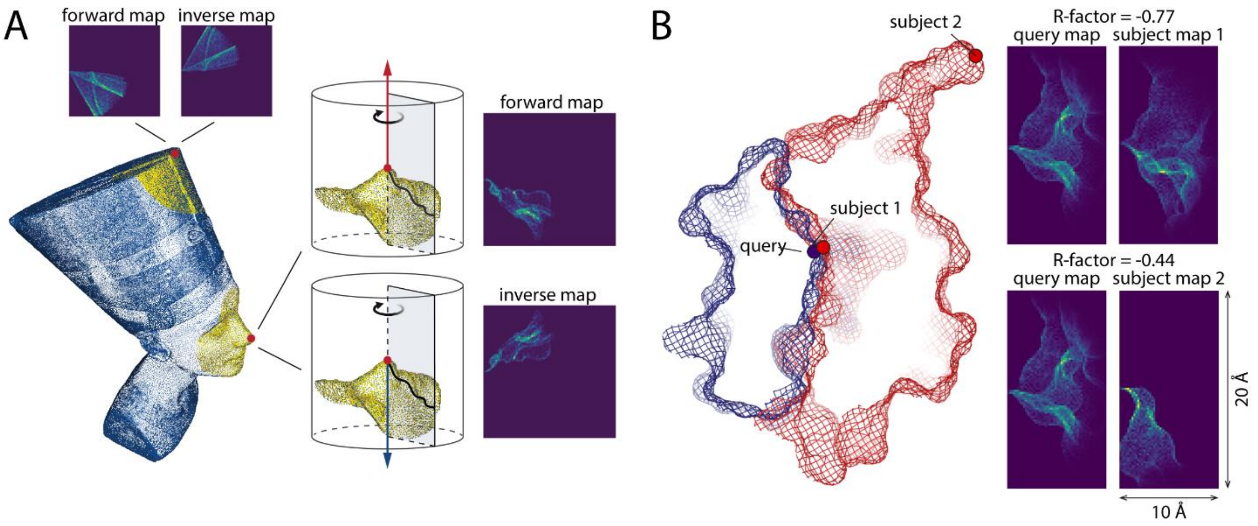

Figure S1. The HECTOR fingerprint captures local surface complementarity. (A) The HECTOR mapper takes a dot-surface of an object (e.g., molecular surface of a protein) as input. Surface patches are extracted around a selected dot (e.g., red dot at the center of the golden face and crown patches from Nefertiti Bust). A map is compiled by rotational accumulation of dot density on a matrix. Depending if the surface normal pointing outwards or inwards of an object, the map is defined as "forward" or "inverse". **(B)** An inverse query map and a forward subject map at complementary interfaces are highly similar and have low R-factor. Shown is an example of a tight interface between two fragments of split ubiquitin (PDB: 1UBQ). Maps were generated using HECTOR v0.2 (Materials and methods). The protein and surface maps are shown at their true relative scales to each other.

Figure S2

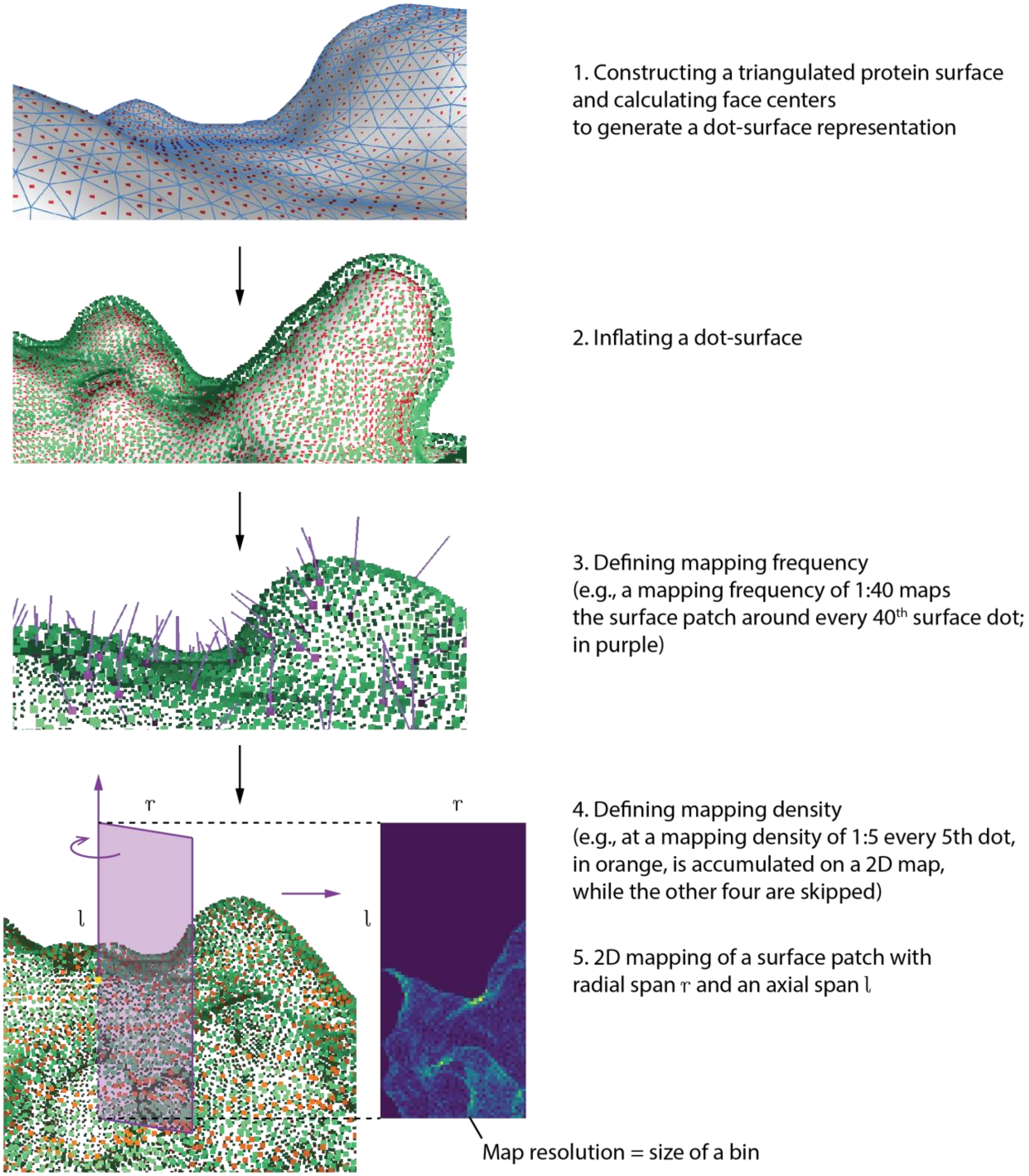

Figure S2. Schematic workflow of the HECTOR mapping approach.

Figure S3

```
1OH0       LPTAQEVQGLMARYIELVDVGDIEAIVQMYADDATVEDPFGQPPIHGREQIAAFYRQGLGGGKV
sam0.1     LPTAQEVQGLMARYIELMDVGDIEAIVQMYADDATVEAPFGAPPIHGRERIAYFYRRMLGGGIA
sam0.2     LPTAEEVQKLMARYIELMDVGDIEAIVQMYADDATVEAPFGAPPIHGRERIAYFYRRMLGEGIA
sam0.3     LPTAEEVQKLMARYIELMDKGDIEAIVQMYADDATVEAPFGAPPIHGRERIAYFYRRMLGEGIA
sam0.4     LPTAEEVQKLMARYIELLDVGDIEAIVQMYADDATVEAPFGSPPIHGRERIAYFYRRMLGEGIA
sam0.5     LPTAEEVQKLMARYIELMDVGDIEAIVQMYADDATVEAPFGAPPIHGRERIAYFYRRMLGGGIA
sam0.6     LPTAEEVQKLMARYIELMDVGDIEAIVQMYADDATVEAPFGAPPIHGRERIAYFYRRMLGGGIA
sam0.7     LPTAEEVQKLMARYIELMDVGDIEAIVQMYADDATVEAPFGAPPIHGRERIAYFYRRMLGGGIA
sam0.8     LPTAEEVQKLMARYIELMDVGDIEAIVQMYADDATVEAPFGAPPIHGRERIAYFYRRMLGGGIA
           ****:*** ********:* ***************** *** *******:** ***: ** * .

1OH0       RACLTGPVRASHNGCGAMPFRVEMVWNGQPCALDVIDVMRFDEHGRIQTMQAYWSEVNLSVREP
sam0.1     RATLTGPVRASHNGTGAMPFRVEFVFNGQPYAMDVRVEMRFDEHGRIQTMQAYWSWVNLSVREP
sam0.2     RATLTGPVRASHNGTGAMPFRVEYVFNGQPYAMDVRVEMRFDEHGRIQTMQAYWSEVNDSVREP
sam0.3     RATLTGPVRASHNGTGAMPFRVEFVFNGQPYAMDVRVEMRFDEHGRIQTMQAYWSWVNLSVREP
sam0.4     RATLTGPVRASHNGTGAMPFRVEYVLNGQPVAMDIRVEMRFDEHGRIQTMQAYWSWVNVSVREP
sam0.5     RATLTGPVRASHNGTGAMPFRVEYVFNGQPYAMDVRVEMRFDEHGRIQTMQAYWSWVNLSVREP
sam0.6     RATLTGPVRASHNGTGAMPFRVEFVFNGQPFAMDVRVEMRFDEHGRIQTMQAYWSWVNLSVREP
sam0.7     RATLTGPVRASHNGTGAMPFRVEYVFNGQPFAMDVRVEMRFDEHGRIQTMQAYWDWVNLSVREP
sam0.8     RATLTGPVRASHNGTGAMPFRVEYVFNGQPYAMDVRVEMRFDEHGRIQTMQAYWSWVNISVREP
           ** *********** ******** * **** *:*:   ****************. ** *****


1PM1       DCSTNISPKQGLDKAKYFSGKWYVTHFLDKDPQVTDQYCSSFTPRESDGTVKEALYHYNA
sima1.1    DCSTNISPKQGLDKAKYFSGKWYLTHVLIKDPVAVTQFCSSFTPRESDGTVKEAIYVYLA
sima1.2    --STNISPKQGLDKAKYFSGKWYLTHVLIKDPVAVTQFCSSFTPRESDGTVKEAIYVYLA
sima2.1    DCSTNISPKQGLDKAKYFSGKWYLTHVLIKDPVAVTQFCSSFTPRESDGTVKEAIYVYLA
sima2.2    --STNISPKQGLDKAKYFSGKWYLTHVLIKDPVAVTQFCSSFTPRESDGTVKEAIYVYLA
sima3.1    DCSTNISPKQGLDKAKYFSGKWYLTHVLIKDPKIVSQFCASFTPRESDGTVKIAVYLYLA
sima3.2    --STNISPKQGLDKAKYFSGKWYLTHVLIKDPKIVSQFCASFTPRESDGTVKIAVYLYLA
sima4.1    DCSTNISPKQGLDKAKYFSGKWYLTHVLIKDPVAVTQFCSSFTPRESDGTVKVAIYVYLA
sima4.2    --STNISPKQGLDKAKYFSGKWYLTHVLIKDPVAVTQFCSSFTPRESDGTVKVAIYVYLA
             *********************:**.* ***  . *:*:************ *:* * *

1PM1       NKKTSFYNIGEGKLESSGLQYTAKYKTVDKKKAVLKEADEKNSYTLTVLEADDSSALVHI
sima1.1    IKKTSEYAIGEGKLESSGLQYTATFKTVDKKKAVLKEWDERYSYTITVLEADDSSALTHV
sima1.2    IKKTSEYAIGEGKLESSGLQYTATFKTVDKKKAVLKEWDERYSYTITVLEADDSSALTHV
sima2.1    IKKTSEYAIGEGKLESSGLQYTATFKTVDKKKAVLKEWDERYSYTITVLEADDSSALTHV
sima2.2    IKKTSEYAIGEGKLESSGLQYTATFKTVDKKKAVLKEWDERYSYTITVLEADDSSALTHV
sima3.1    IKKTSDYAIGEGKLESSGLQYTATSKVVDKKKAVLKELDERHSYTVTVLEADDSSALTHI
sima3.2    IKKTSDYAIGEGKLESSGLQYTATSKVVDKKKAVLKELDERHSYTVTVLEADDSSALTHI
sima4.1    IKKTSEYAIGEGKLESSGLQYTATFKTVDKKKAVLKEFDERHSYTITVLEADDSSALVHV
sima4.2    IKKTSEYAIGEGKLESSGLQYTATFKTVDKKKAVLKEFDERHSYTITVLEADDSSALVHV
            **** * ***************. *.********** **: ***:***********.*:

1PM1       CVREGSKDLGDVYTVLTHQKDAEPSAKVKSAVTQAGLQLSQFVGTKDLGCQYDDQFTSL
sima1.1    CTREGSKDYGDYYHVLTHQKDAEPSAKVKSAVTQAGLQLSQFVGTKDLGCQYDDQFTSL
sima1.2    TTREGSKDYGDYYHVLTHQKDAEPSAKVKSAVTQAGLQLSQFVGTKDLGCQYDDQFTSL
sima2.1    CTREGSKDYGDYYMVLTHQKDAEPSAKVKSAVTQAGLQLSQFVGTKDLGCQYDDQFTSL
sima2.2    TTREGSKDYGDYYMVLTHQKDAEPSAKVKSAVTQAGLQLSQFVGTKDLGCQYDDQFTSL
sima3.1    CVREGSKDYGDYYLVLTHQKDAEPSAKVKSAVTQAGLQLSQFVGTKDLGCQYDDQFTSL
sima3.2    TVREGSKDYGDYYLVLTHQKDAEPSAKVKSAVTQAGLQLSQFVGTKDLGCQYDDQFTSL
sima4.1    CVREGSKDYGDYYNVLTHQKDAEPSAKVKSAVTQAGLQLSQFVGTKDLGCQYDDQFTSL
sima4.2    TVREGSKDYGDYYNVLTHQKDAEPSAKVKSAVTQAGLQLSQFVGTKDLGCQYDDQFTSL
            .****** ** * *********************************************
```

Figure S3. Amino acid sequences of the Sam/Sima templates and designed proteins. Residues predicted to interact with the target epitope are highlighted in red.

Figure S4

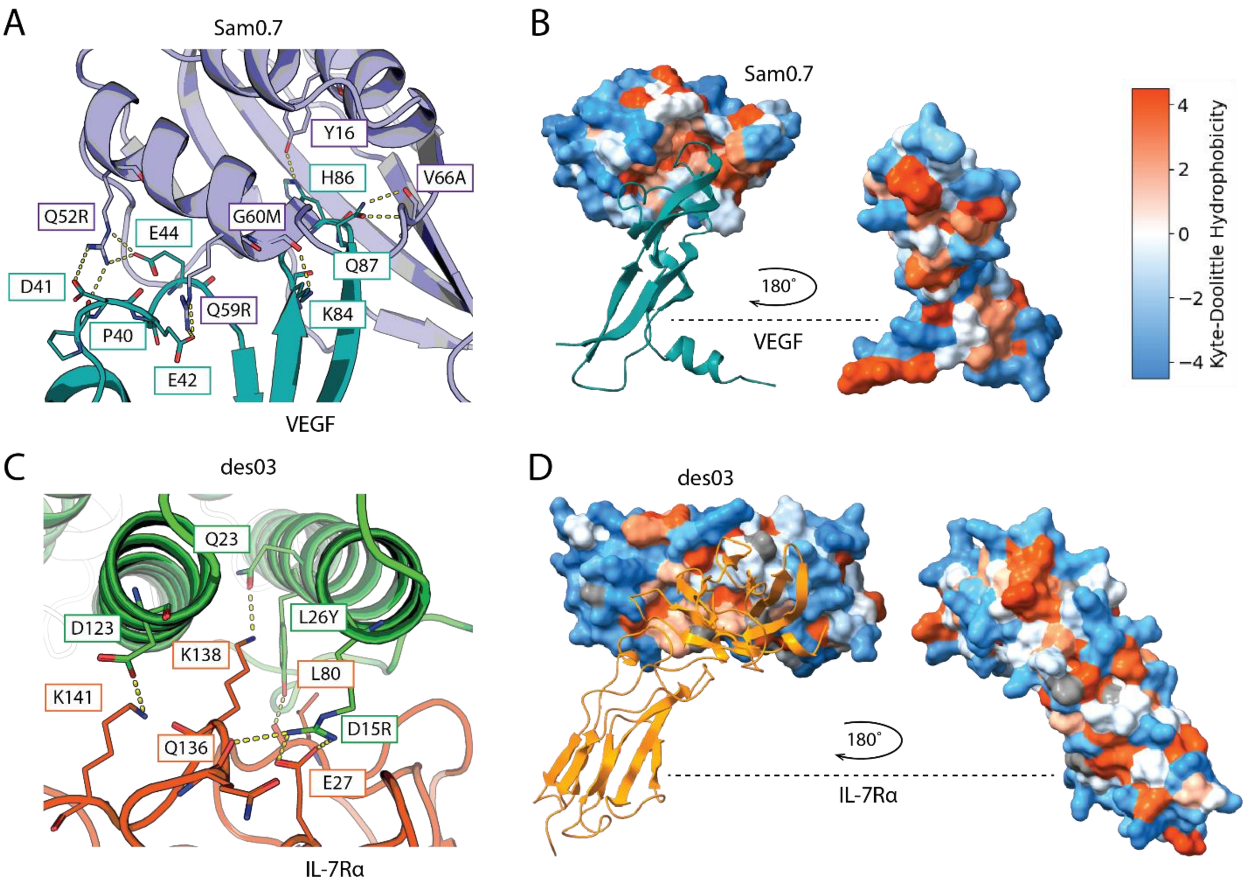

Supplementary figure 4. Hydrogen bonds and hydrophobic interactions at the designed interfaces of the anti-VEGF (Sam0.7) and IL-7Rα (des03) binders. (A, C) Both wild-type and designed residues are predicted to form hydrogen bonds (yellow dashed lines) with side-chains of the target. **(B, D)** Hydrophobicity profiles of Sam0.7 and des03 show the designed interfaces to have hydrophobic patches that are expected to interact with complementary hydrophobic regions (red) on the surfaces of their respective targets, VEGF and IL-7Rα.

Figure S5

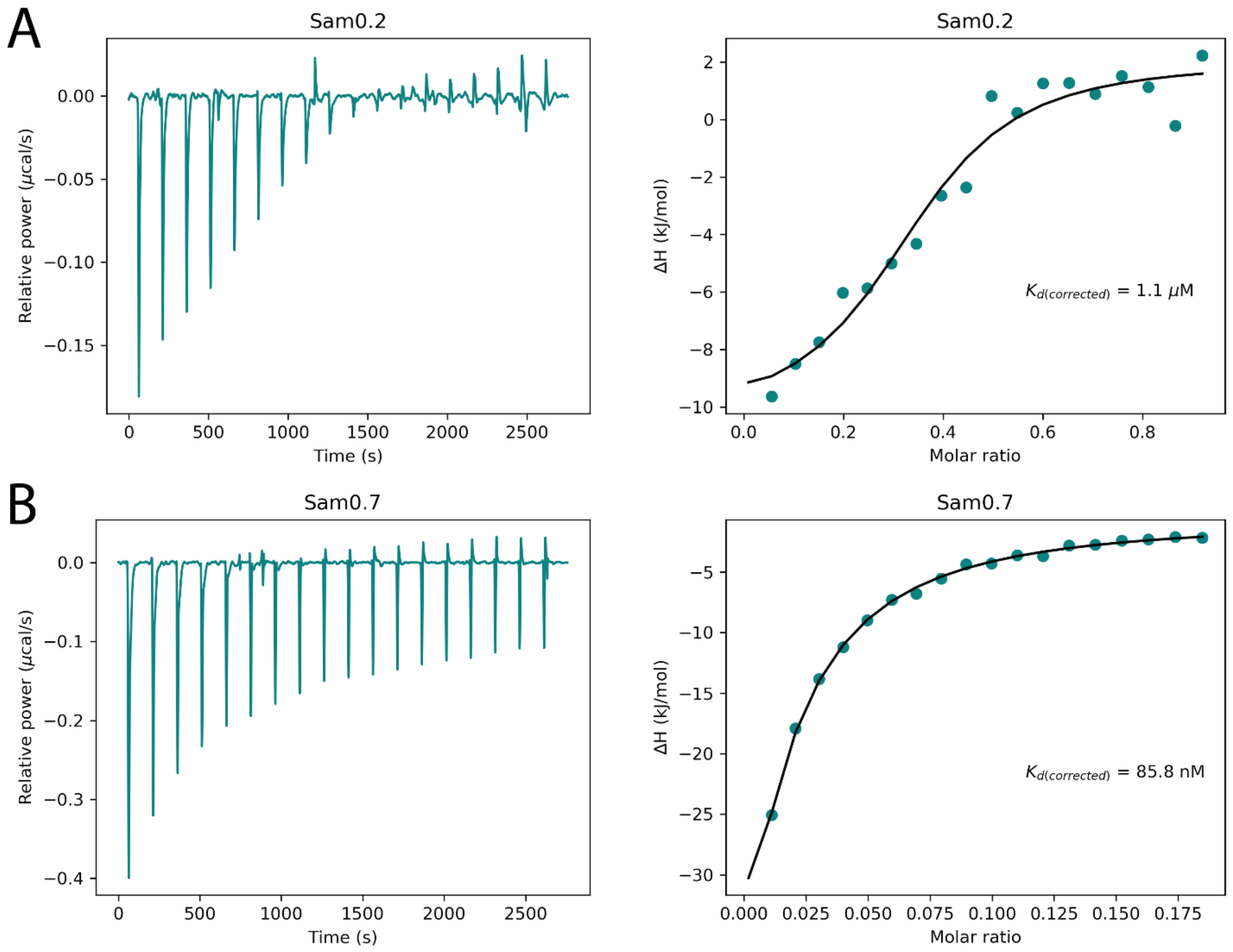

Supplementary figure 5. Calorigrams (left) and corresponding fitted curves (right) obtained from isothermal titration of VEGF with Sam0.2 (A) and Sam0.7 (B).

Figure S6

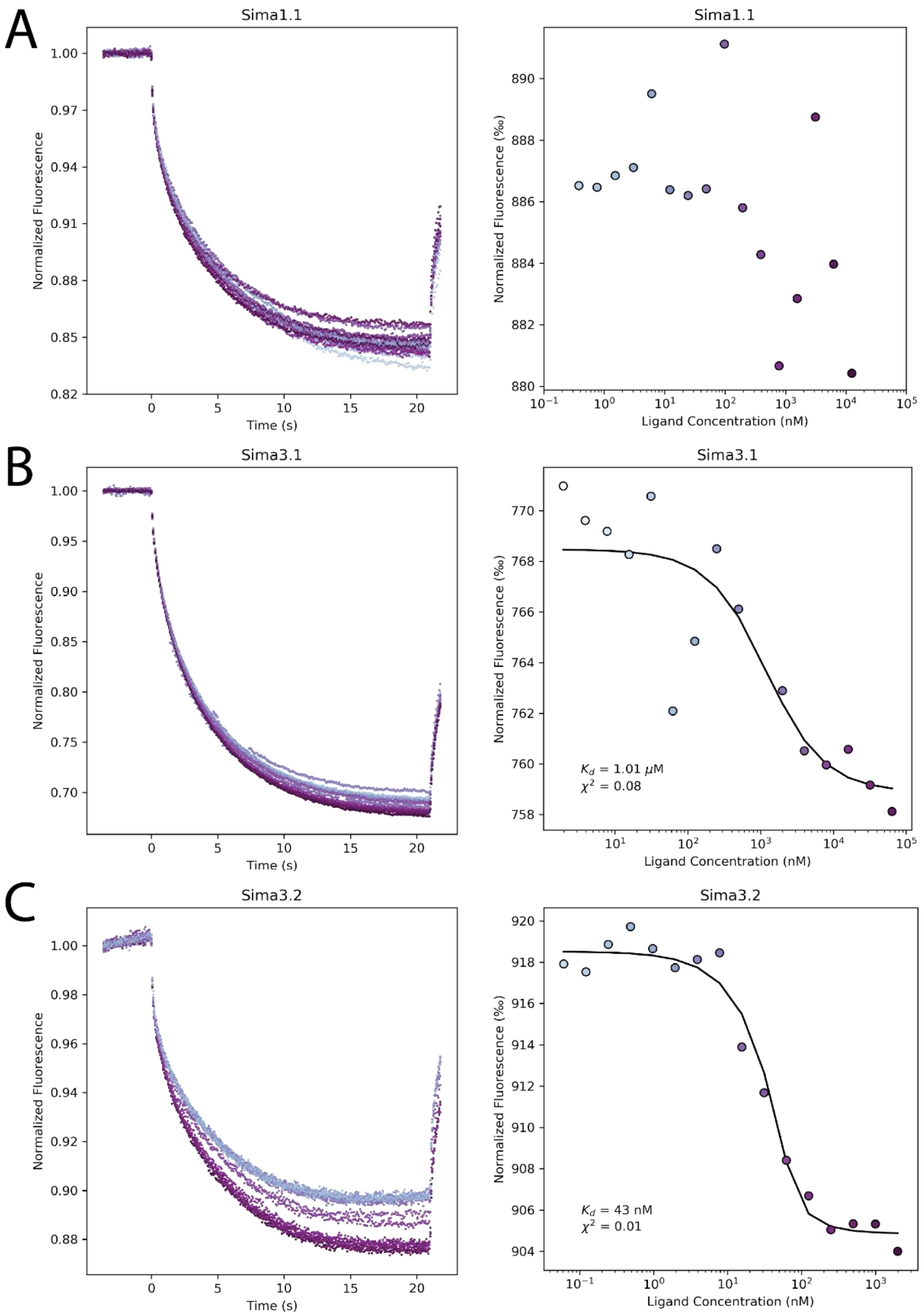

Supplementary figure 6. Microscale thermophoresis traces (left) and dose-response curves (right) for the binding interaction between VEGF and Sima designs. Results are shown for three Sima designs (Sima1.1, Sima3.1, Sima3.2) that had the best expression yield.

Figure S7

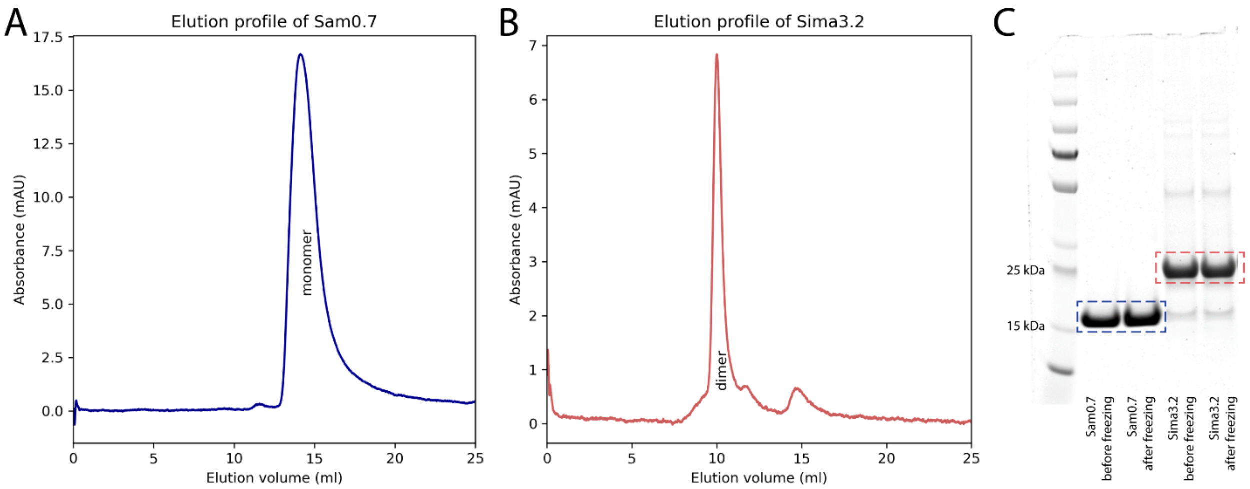

Supplementary figure 7. Purification of Sam0.7 and Sima3.2 designs. (A, B) Analytical size exclusion chromatogram shows that the final product of Sam0.7 elutes as a monomer, while the final product of Sima3.2 elutes as a dimer. **(C)** SDS-PAGE of Sam0.7 and Sima3.2 final products (before and after freezing) used for experimental evaluation.

Figure S8

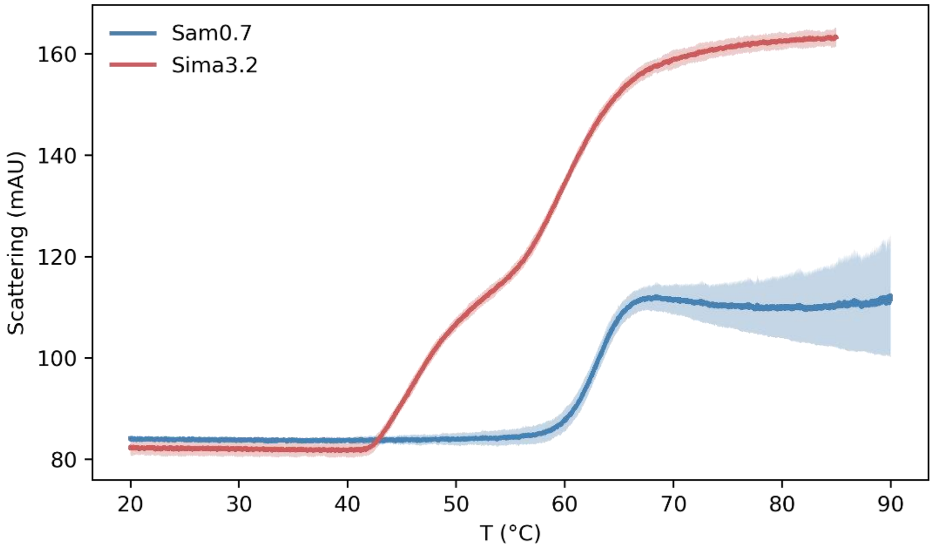

Supplementary figure 8. Light scattering thermograms of Sam0.7 and Sima3.2 designs. Thermograms indicate that Sima3.2 has higher propensity for aggregation with an onset temperature of around 40 °C, compared to an onset temperature of 60 °C for Sam0.7. Shades represent the standard deviation across three replicates.

Figure S9

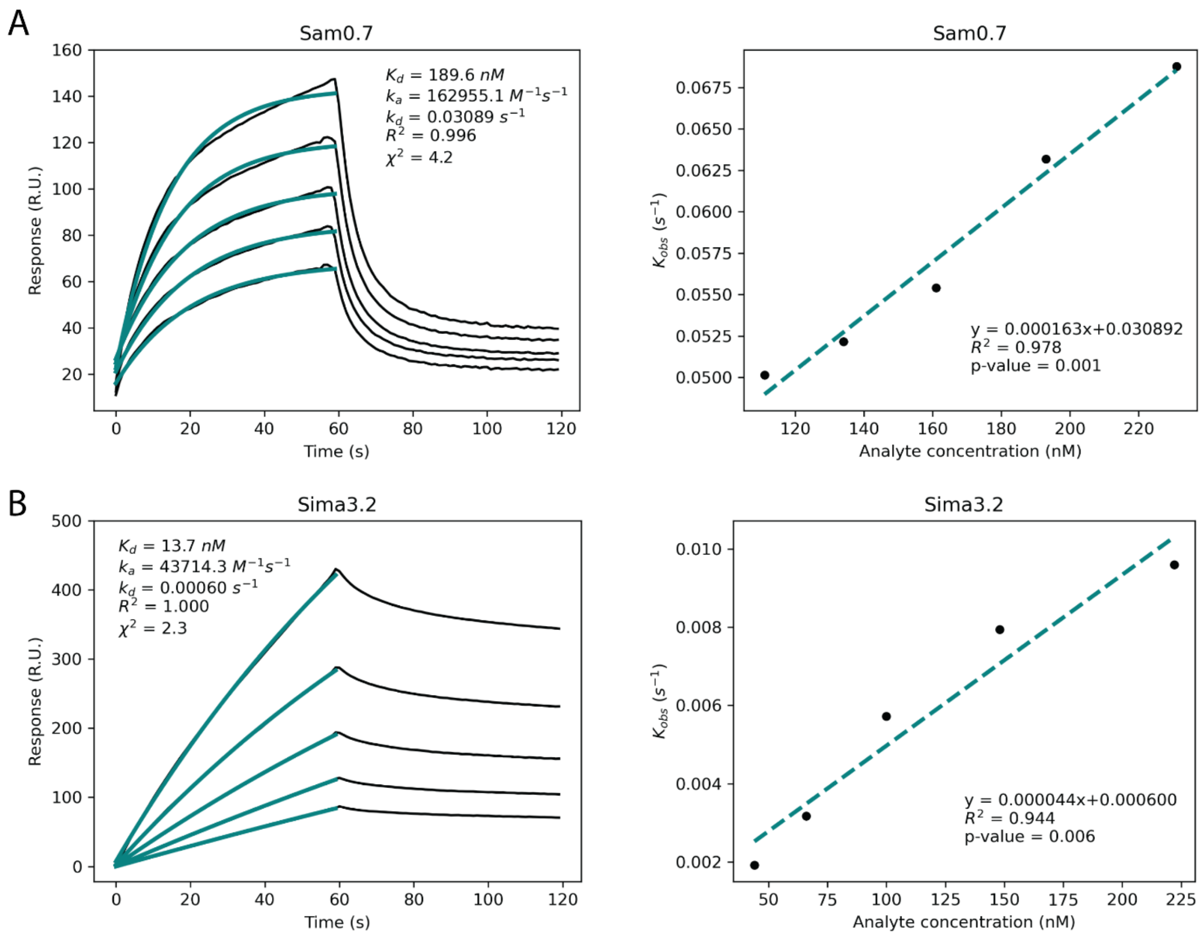

Supplementary figure 9. SPR sensorgrams of (A) Sam0.7 and (B) Sima3.2 and their VEGF binding kinetics fits. Sensograms (presented also in Fig. 2D) and association phase fits are shown against their respective k_{obs} fits.

Figure S10

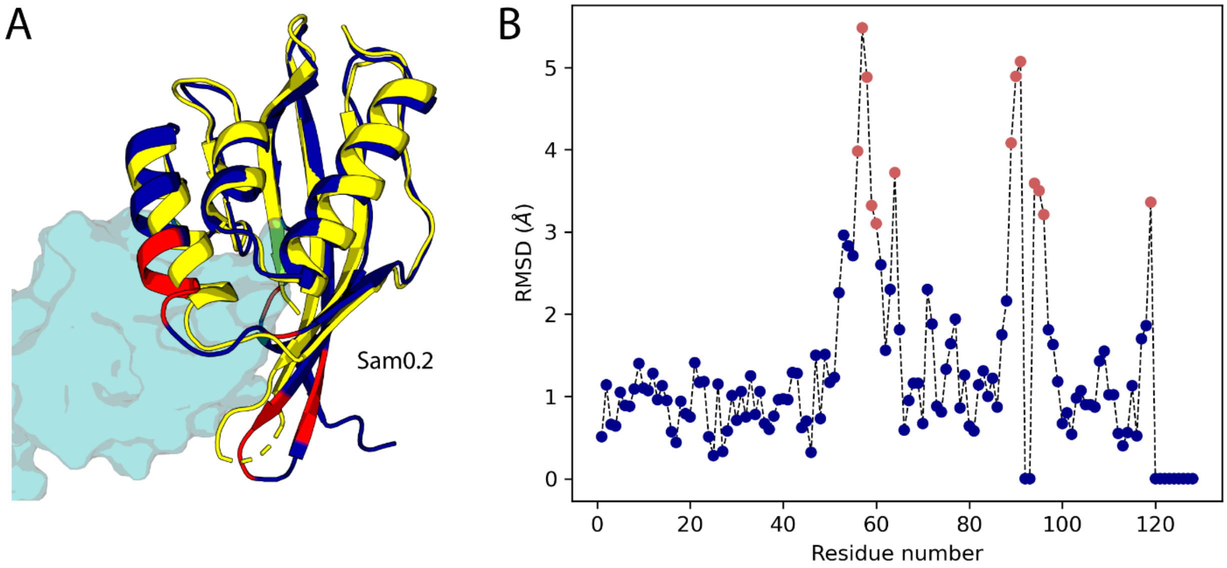

Supplementary figure 10. Crystal structure of Sam0.2 matches the computational design model with atomic-level accuracy. (A) Superimposition of the Sam0.2 design model (blue) and the experimentally determined crystal structure (yellow). The VEGF epitope, in its modeled orientation relative to Sam0.2, is shown as a teal surface. Regions in the design model with RMSD values higher than 3 Å are highlighted in red. **(B)** The scatterplot displays RMSD between coordinates of the C_α atoms in the Sam0.2 design model and corresponding C_α atoms in the crystal structure. Residues in gaps are assigned an RMSD value of 0.

Figure S11

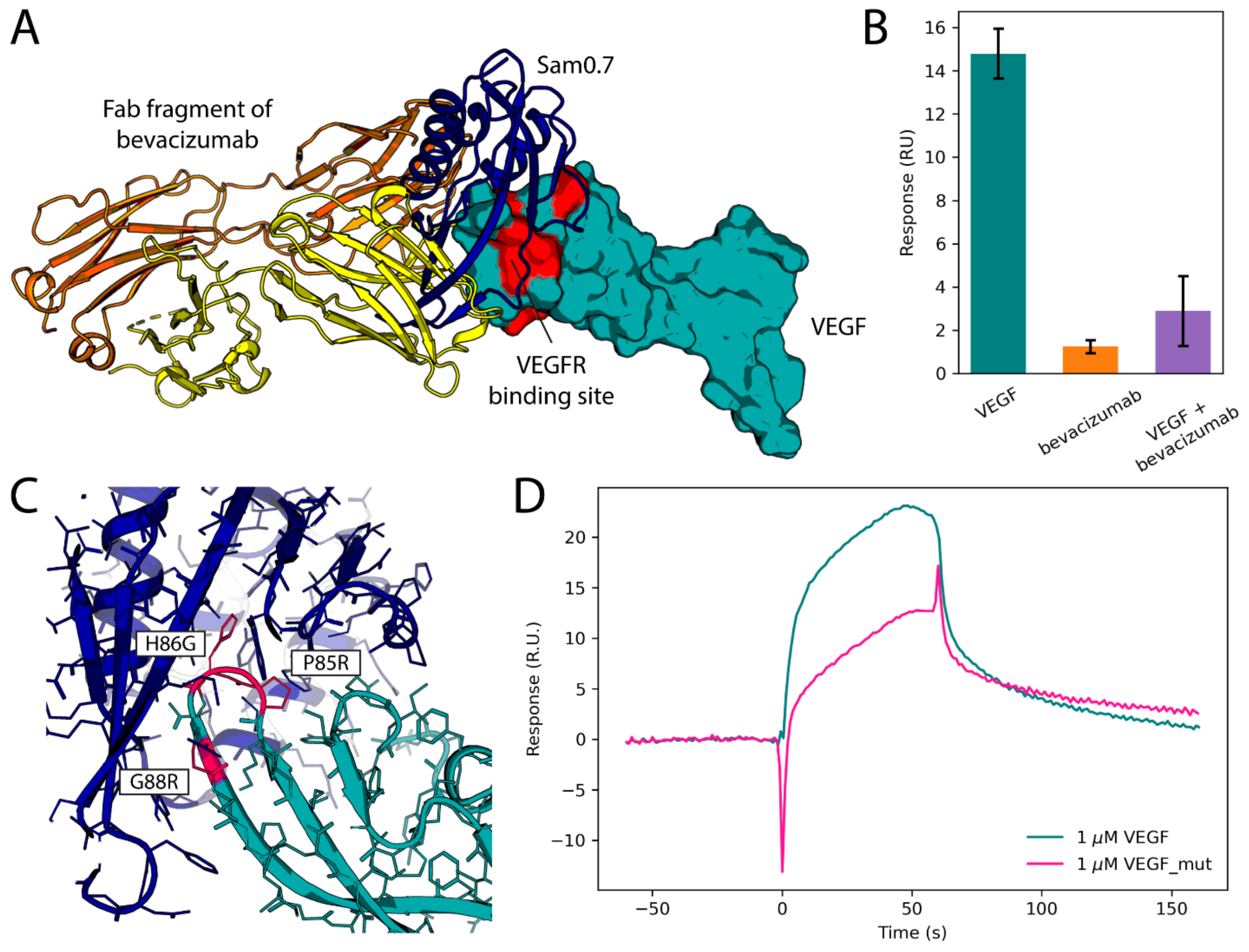

Supplementary figure 11. Characterization of Sam0.7:VEGF binding mode. (A) Model of the VEGF:Sam0.7 complex aligned with the crystal structure of the complex between VEGF and Fab fragment of bevacizumab (PDB: 1BJ1). The binding epitopes of Sam0.7 and bevacizumab on VEGF show partial overlap. **(B)** For the SPR-based competition assay, Sam0.7 was immobilized on a CM5 chip using standard amine coupling chemistry. As analytes, 500 nM VEGF, 100 nM bevacizumab, or a mixture of 500 nM VEGF and 100 nM bevacizumab were used. The bar plot shows the average response at the end of injection, with error bars representing the standard deviations across three replicates for each analyte. The results indicate that bevacizumab competes with immobilized Sam0.7 for VEGF binding. **(C)** Three VEGF residues at the expected interface with Sam0.7 were mutated as follows: P85R, H86G, G88R. To evaluate an effect of these mutations on VEGF:Sam0.7 binding, we perform an SPR experiment with immobilized Sam0.7. **(D)** The mutated VEGF showed weaker binding to Sam0.7 compared to the wild-type, indicating the importance of the mutated residues for the interaction.

Figure S12

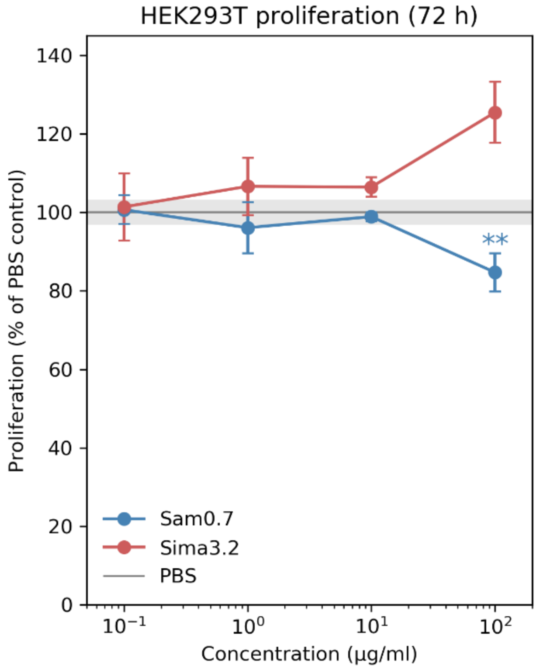

Figure S12. The anti-VEGF designs did not show a strong inhibitory effect on proliferation of the human embryonic kidney cell line. Proliferation of HEK293T cells was mostly not affected by the designed binders. Only treatment with the highest concentration of Sam0.7 (100 µg/ml) could decrease the cell growth. Error bars represent the standard deviations across three replicates from one experiment. Statistical significance was calculated using Fisher's one-sided t-test (**, $p \leq 0.01$ vs. the PBS group).

Figure S13

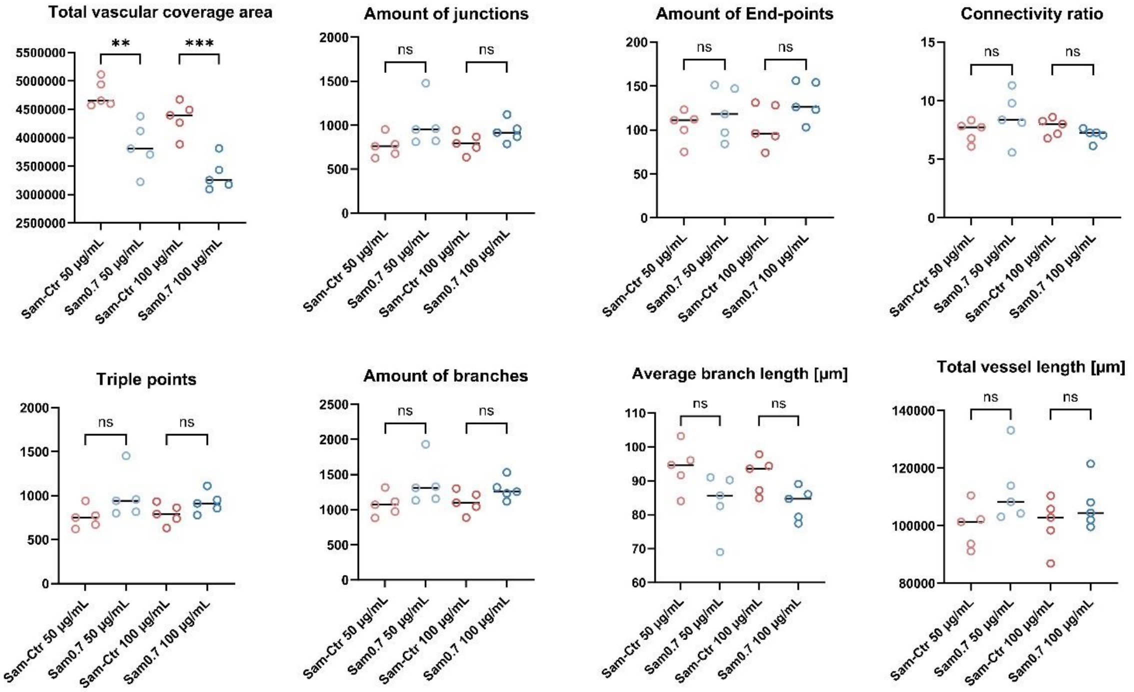

Supplementary figure S13. The effect of the designed binder Sam0.7 and its initial scaffold Sam_cntrl on microvasculature formation *in vitro*. Quantification of microvasculature parameters of capillaries treated with Sam0.7 or Sam_cntrl (as a negative control). The connectivity ratio is defined as the ratio of junctions to end-points. Statistical significance was calculated using the one-way ANOVA test (** $p \leq 0.01$, *** $p \leq 0.001$ treated Sam0.7 vs. Sam_cntrl group).

Figure S14

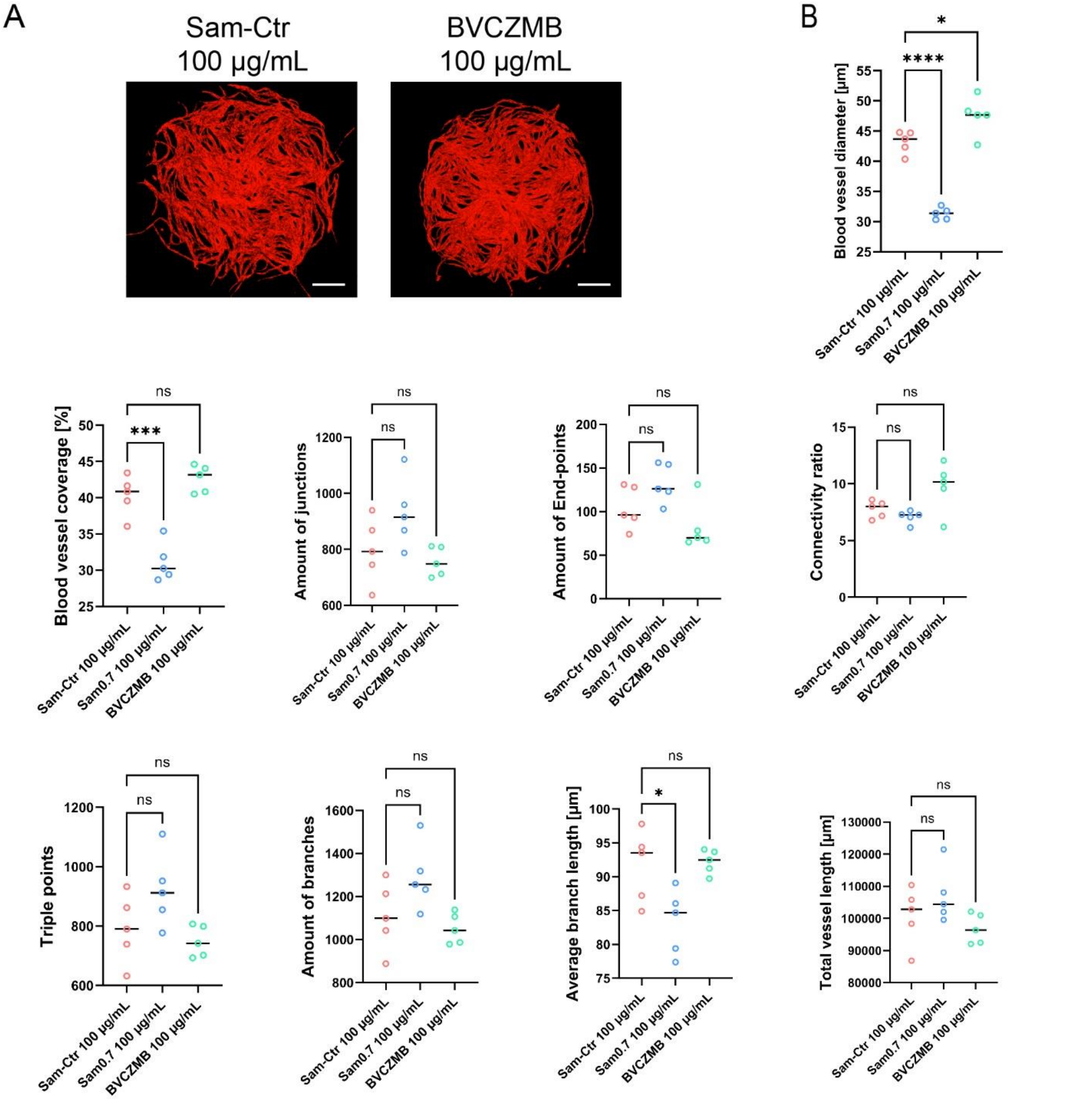

Supplementary figure S14. The effect of bevacizumab on the microvasculature formation *in vitro*. (A) Representative images showing *in vitro* microvasculature formation in the presence of bevacizumab or sam_cntrl as a negative control at the same working concentrations (100 µg/mL). The scale bar is 500 µm. **(B)** Quantitative analysis of microvasculature formation. Statistical significance was calculated using the one-way ANOVA test (* $p \leq 0.05$, *** $p \leq 0.001$, **** $p \leq 0.0001$ treated Sam0.7 or bevacizumab vs. Sam_cntrl group).

Figure S15

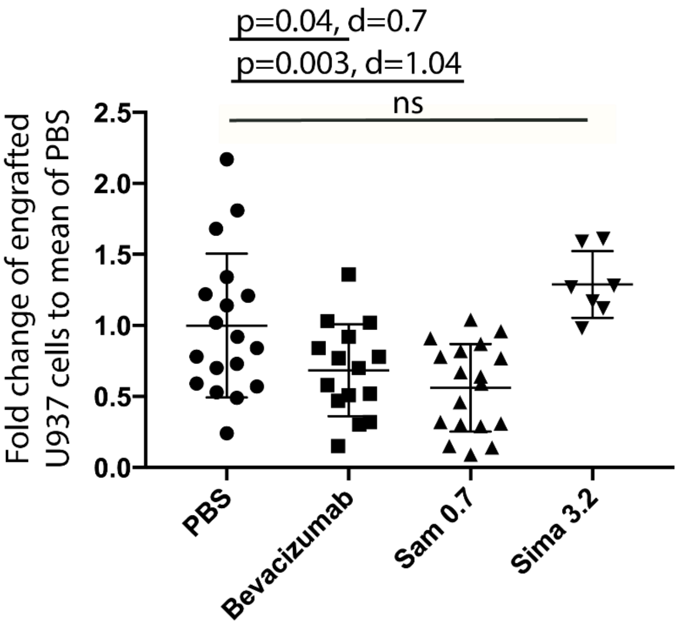

Supplementary figure 15. Evaluation of inhibitory activity of the designs in leukemia zebrafish xenograft. Quantification of the engrafted U937-GFP leukemia cells in zebrafish embryos that were injected with PBS, bevacizumab as positive control, Sam 0.7, or Sima 3.2. Each dot indicates one embryo. p-value was calculated by Mann Whitney two tailed test. d – Cohen's d value.

Figure S16

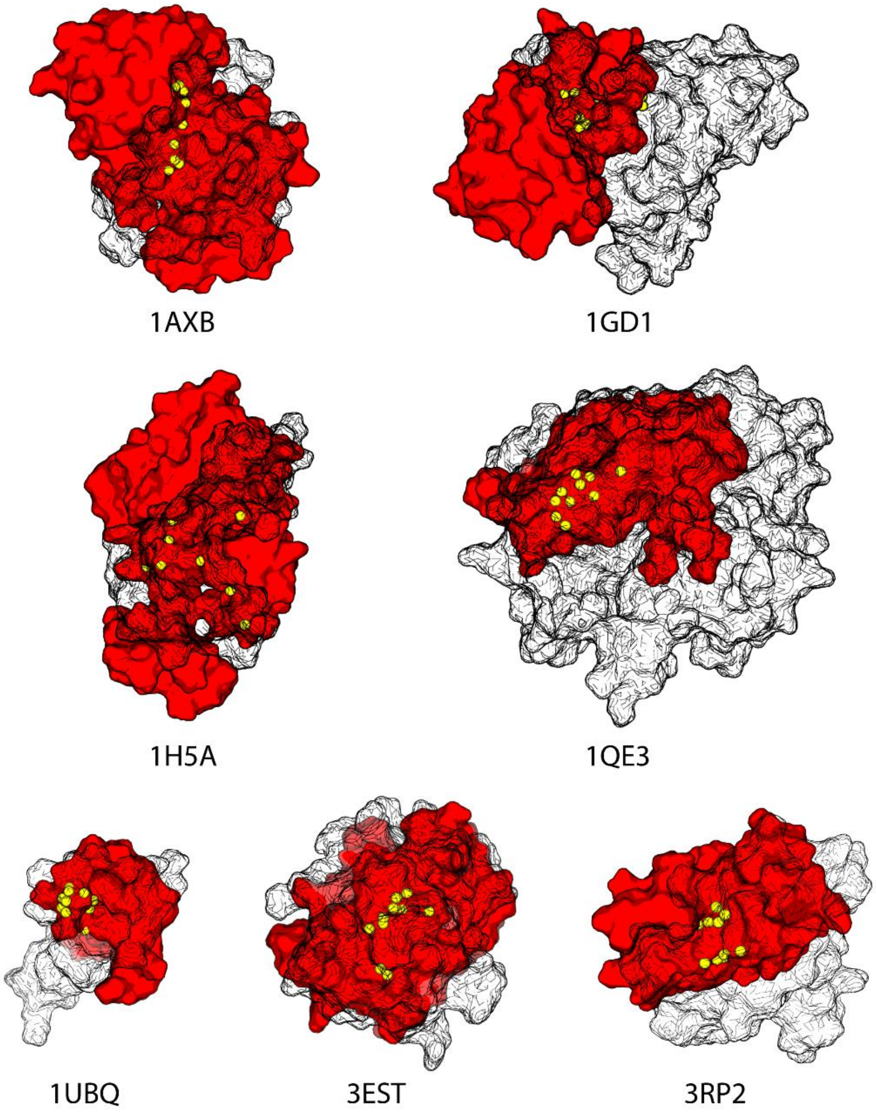

Supplementary figure 16. Dataset of proteins used for HECTOR benchmarking. Surface representation of split proteins consisting of two complementary fragments. The first fragment (red) was used as the query protein, while the second fragment (transparent) was used as the subject protein. Yellow spheres represent ten query dots (i.e., centers of query maps) selected at the interface between the two fragments. For benchmarking, each unique pair of query maps selected from these ten maps was compared against all subject maps (i.e., two-vs-all).

Figure S17

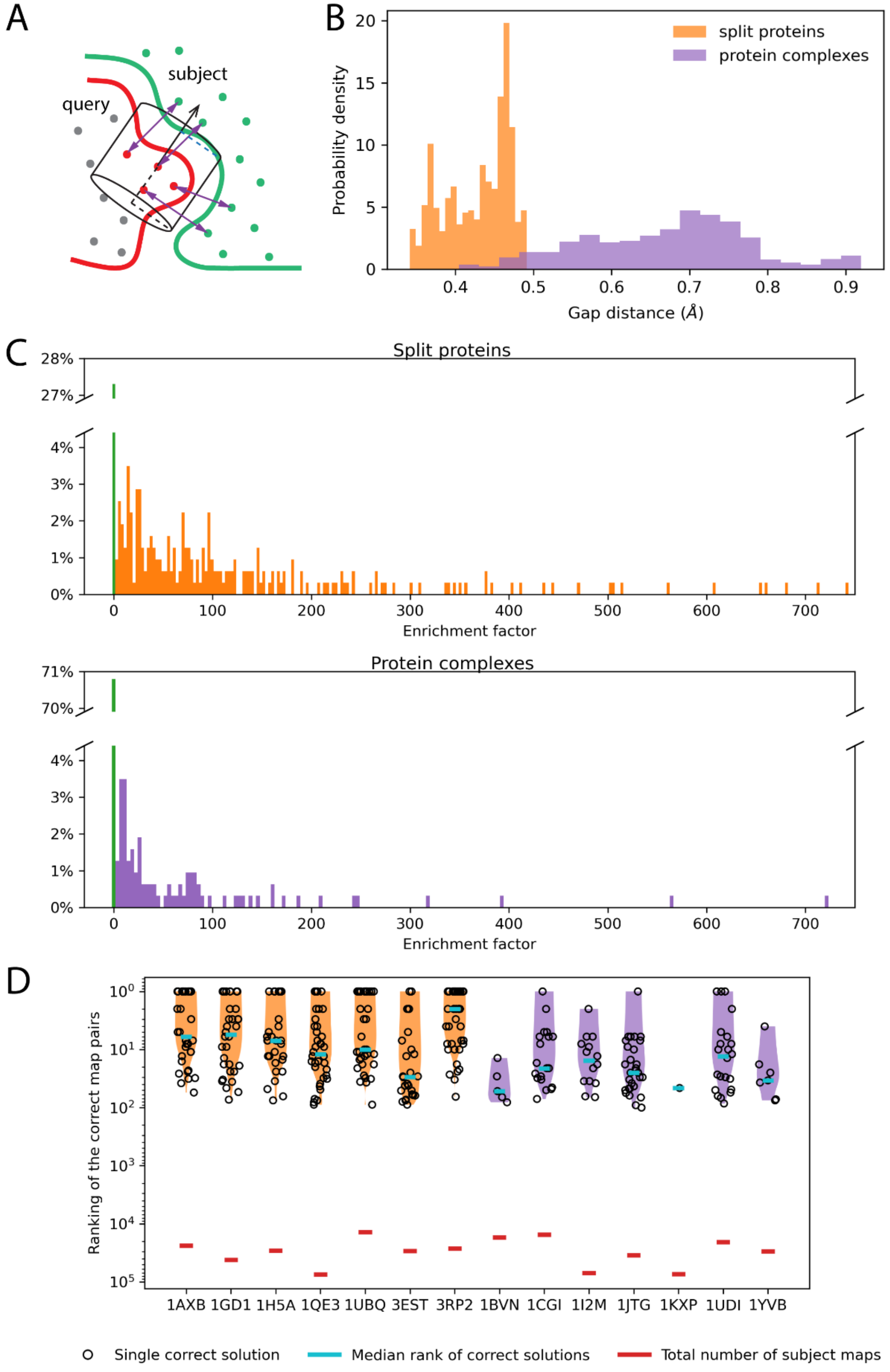
A
query
subject
B
split proteins
protein complexes
Probability density
Gap distance (Å)
C
Split proteins
Enrichment factor
Protein complexes
Enrichment factor
D
Ranking of the correct map pairs
1AXB 1GD1 1H5A 1QE3 1UBQ 3EST 3RP2 1BVN 1CGI 1I2M 1JTG 1KXP 1UDI 1YVB
Single correct solution
Median rank of correct solutions
Total number of subject maps

Supplementary figure 17. Benchmarking of HECTOR method for complementarity evaluation. For benchmarking, a set of highly complementary interfaces is needed. To assess level of complementarity between two proteins (or protein fragments), we used a "gap distance" score. **(A)** Proteins were represented by their dot surfaces. For each interfacial dot on the query protein, neighboring surface dots within a cylindrical cutoff (radius 5 Å, height 20 Å) were grouped into the same patch. The gap distance for each query dot was calculated as the distance to the nearest dot on the subject protein, and the patch's gap distance was the average of all individual dot distances. **(B)** The distribution of gap distances for split proteins (PDB: 1AXB, 1GD1, 1H5A, 1QE3, 1UBQ, 3EST, 3RP2) and natural protein-protein complexes (PDB: 1BVN, 1CG1, 1I2M, 1JTG, 1KXP, 1UDI, 1YVB) shows that split proteins have more complementary interfaces; therefore, they were used as the main dataset for HECTOR benchmarking. Meanwhile, the set of protein-protein complexes was useful for testing HECTOR's sensitivity to interface irregularities. For the shown histogram, 100 interfacial dots with the lowest gap scores were used. **(C)** HECTOR could effectively identify pairs of subject maps that correspond to selected pairs of query maps at the interface of split protein fragments. The graph shows the distribution of the enrichment factor, which was calculated as:

$$\frac{N\ correct\ subject\ pairs\ identified\ in\ top 100}{100} \Big/ \frac{N\ possible\ solutions}{Total\ N\ of\ subject\ maps\ pairs\ with\ inter-patch\ distance\ d}.$$

The green bar represents cases where no correct solutions were found within the top 100, accounting for approximately 27 % of cases in the split protein dataset and 70 % in the dataset of protein complexes. **(D)** The violin plot shows the distribution of the correct maps' rankings within the top 100 HECTOR hits.

Figure S18

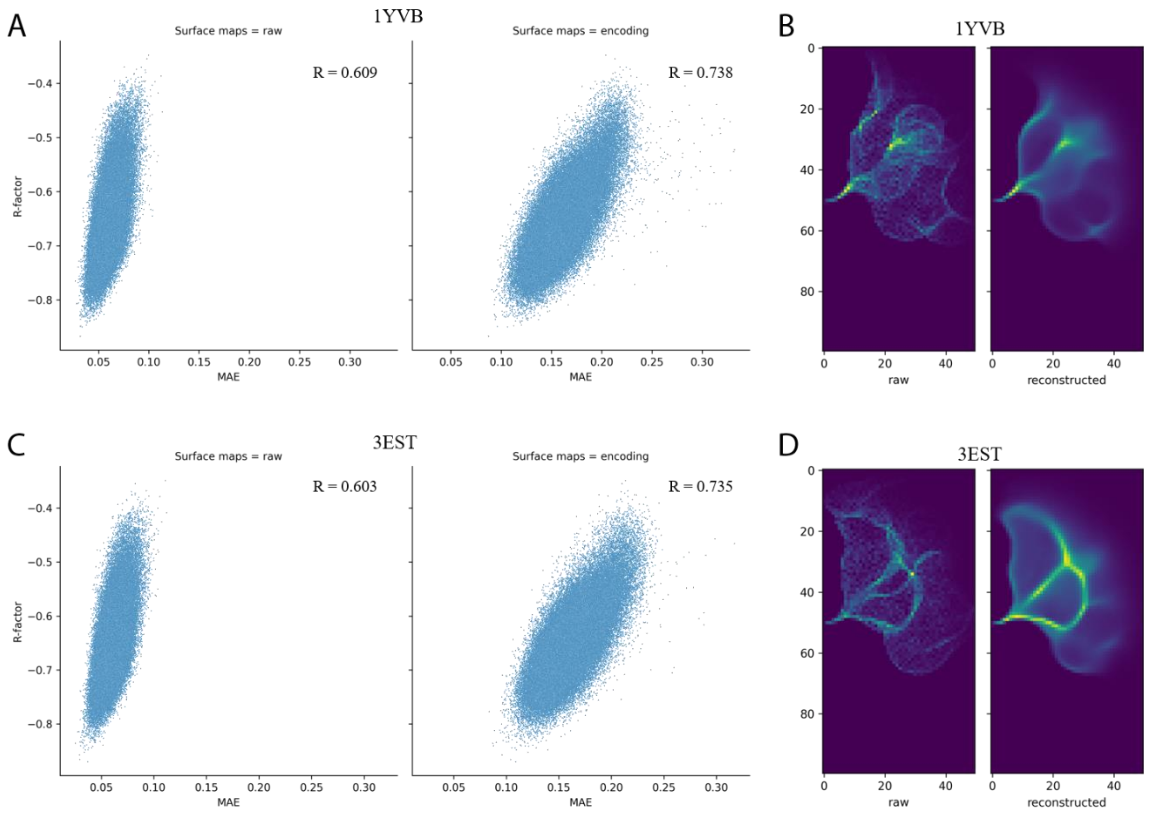

Supplementary figure 18. Compression of HECTOR surface maps into smaller embeddings. (A, C) Correlation between the distribution of pairwise R-factor and embeddings mean absolute error (MAE) of HECTOR maps describing the surface of two different protein structures; 1YVB (A) or 3EST (C). The scatter plots show the comparison between using R-factors derived directly from the raw maps (left) or indirectly from the reconstructed maps (right). Pearson correlation coefficient (R) is indicated on each plot. **(B, D)** Visualization of example raw maps (left) or their reconstructed form using the learnt embeddings (right).

Figure S19

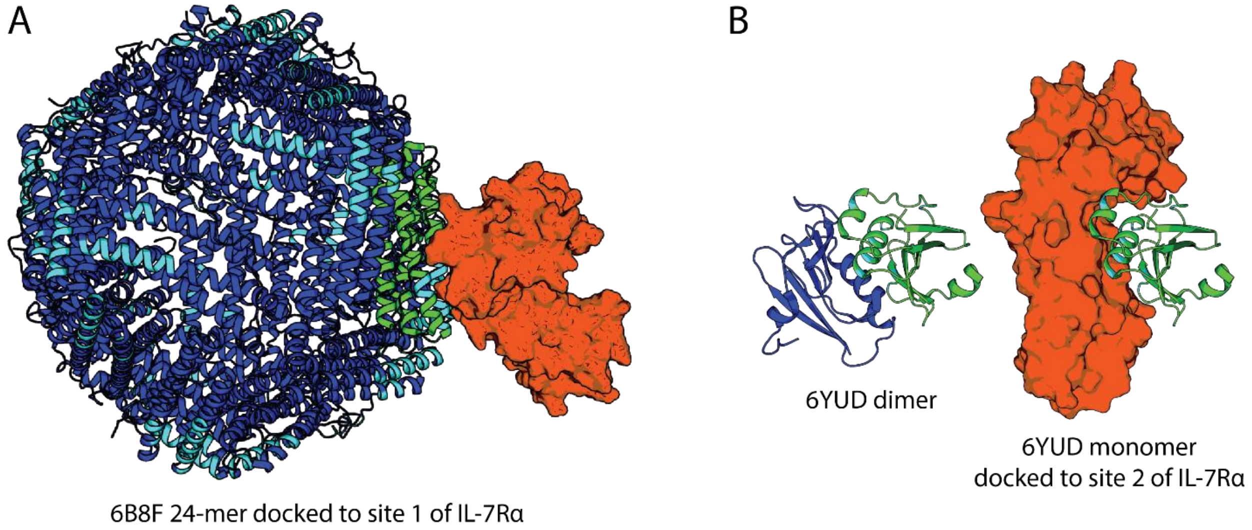

Figure S19. Oligomerization interfaces of 6B8F and 6YUD scaffolds. (A) The 6B8F scaffold assembles into a 24-mer. The interface of the protomer that engages site 1 of IL-7Rα (cyan) does not overlap with the protomer's oligomerization interface. **(B)** The 6YUD scaffold forms a dimer, where the dimerization interface coincides with the interface complementary to site 2 of IL-7Rα (cyan) and was therefore mutated during binder design.

Figure S20

```
5NLC       QIAERLASLRSQLPPSVQLIAVSKNHPAAAIREAYAAGQRHFGENRVQEAIAKQAELTDL
des01      QIAERLRKLREQLPPSVWLIAVSKNHPAAAIREAYKAWQWIFGENRVQEAIRKQAELTDL
des02      QIAERLKKLRRQLPPSVFLIAVSKNHPAAAIREAYRAWQLWFGENRVQEAIRKQAELTDL
           ****** .** ****** ***************** * *  ********** ********

5NLC       PDLTWHLLGKLQSNKARKAVEHFDWIHSVDSWALAERLDRIAGELGRSPKLCLQVKLLPD
des01      PDLWWHLLGKLQSNKARKAVEHFHWIHSVDSWALAERLDRIAGELGRMPHLSLQVKLLPD
des02      PDLMWHLLGKLQSNKARKAVEHFWWIHSVDSWALAERLDRIAGELGRWPHLCLQVKLLPD
           *** ******************* *********************** *:*.********

5NLC       PNKAGWDPADLRAELPQLSQLQQVQIRGLMVIAPLGLTAAETQALFAQARTFAAELQQQA
des01      PNKAGWDPKDLRKELPQLSQLQQVYIWGLMVIAPLGLTAAETQRLFKQARRFARELQQQA
des02      PNKAGWDPKDLRRELPQLSQLEQVRIMGLMVIAPLGLTAAETQRLFRQAREFARELQQQA
           ******** *** ********:** * **************** ** *** ** ******

5NLC       PQLRLTELSMGMSSDWPLAVAEGATWIRVGTQLFGP
des01      PQLRLYVLSMGMSSDWPLAVAEGATWIRVGTQLFGP
des02      PQLRLWFLSMGMSSDWPLAVAEGATWIRVGTQLFGP
           *****  *****************************


6B8F       TSQVRQNYHQDSEAAINRQINLELYASYVYLSMSYYFDRDDVALKNFAKYFLHQSHEERE
des03      TSQVRQNYHQRAERAINMQIVYELYASYVYLSMSYYFDRDDVALKNFAKYFLHQSHEERE
des04      TSQVRQNYHQRAEQAINMQINYELYASYVYLSMSYYFDRDDVALKNFAKYFLHQSHEERE
des06      TSQVRQNYHQKAEKAINMQIMYELYASYVYLSMSYYFDRDDVALKNFAKYFLHQSHEERE
des05      TSQVRQNYHQRAERAINWQIVYELYASYVYLSMSYYFDRDDVALKNFAKYFLHQSHEERE
           ********** :* *** **  **************************************

6B8F       HAEKLMKLQNQRGGRIFLQDIQKPDEDDWESGLNAMEAALHLEKNVNQSLLELHKLATDK
des03      HAEKLMKLQNQRGGRIFLQRIWHPWEDDWESGLNAMEAALHLEKMVNHMLLMLHKLATDK
des04      HAEKLMKLQNQRGGRIFLMKIWHPYEDDWESGLNAMEAALRYEKWVNHMLLWLHKLATDK
des06      HAEKLMKLQNQRGGRIFLYRIWHPYEDDWESGLNAMEAALRMEKWVNHMLLWLHKLATDK
des05      HAEKLMKLQNQRGGRIFLYKIWHPWEDDWESGLNAMEAALRYEKWVNHHLLWLHKLATDK
           ******************  * :* ***************: ** **: ** ********

6B8F       NDPHLADFIETHYLNEQVKAIKELGDHVTNLRKMGAPESGLAEYLFDKHTLG
des03      NDPHLADFIETHYLNEQVKAIKELGDHVTNLRKMGAPESGLAEYLFDKHTLG
des04      NDPHLADFIETHYLNEQVKAIKELGDHVTNLRKMGAPESGLAEYLFDKHTLG
des06      NDPHLADFIETHYLNEQVKAIKELGDHVTNLRKMGAPESGLAEYLFDKHTLG
des05      NDPHLADFIETHYLNEQVKAIKELGDHVTNLRKMGAPESGLAEYLFDKHTLG
           ****************************************************


6YUD       SMKFAVIDRKNFTLIHFEIEKPIKPEILKEIEIPSVDTRKGVVISGRGPIWLHCFLAHKY
des07      SMKFAVIDRKNFTLIHFEIEKPIKWSIMREIEIPSVDTRKGVVISGRGPMWLHCWLVHFY
des08      SMKFAVIDRKNFTLIHFEIEKPIKWTIMREIEIPSVDTRKGVVISGRGPYWLHVWLAHMY
           ************************  *::******************** *** :*.* *

6YUD       AHTPFVAVYDPRLGAVVVQSHSELREGDVIDVVVEEILK
des07      AHTPFVAVYDPRLGAVVVISHSELREGDVIDVVVEEIL-
des08      AHTPFVAVYDPRLGAVVMISHSELREGDVIDVVVEEIL-
           *****************: *******************
```

Figure S20. Amino acid sequences of the designed IL-7Rα binders and their templates. Residues predicted to interact with the target epitope are highlighted in red.

Figure S21

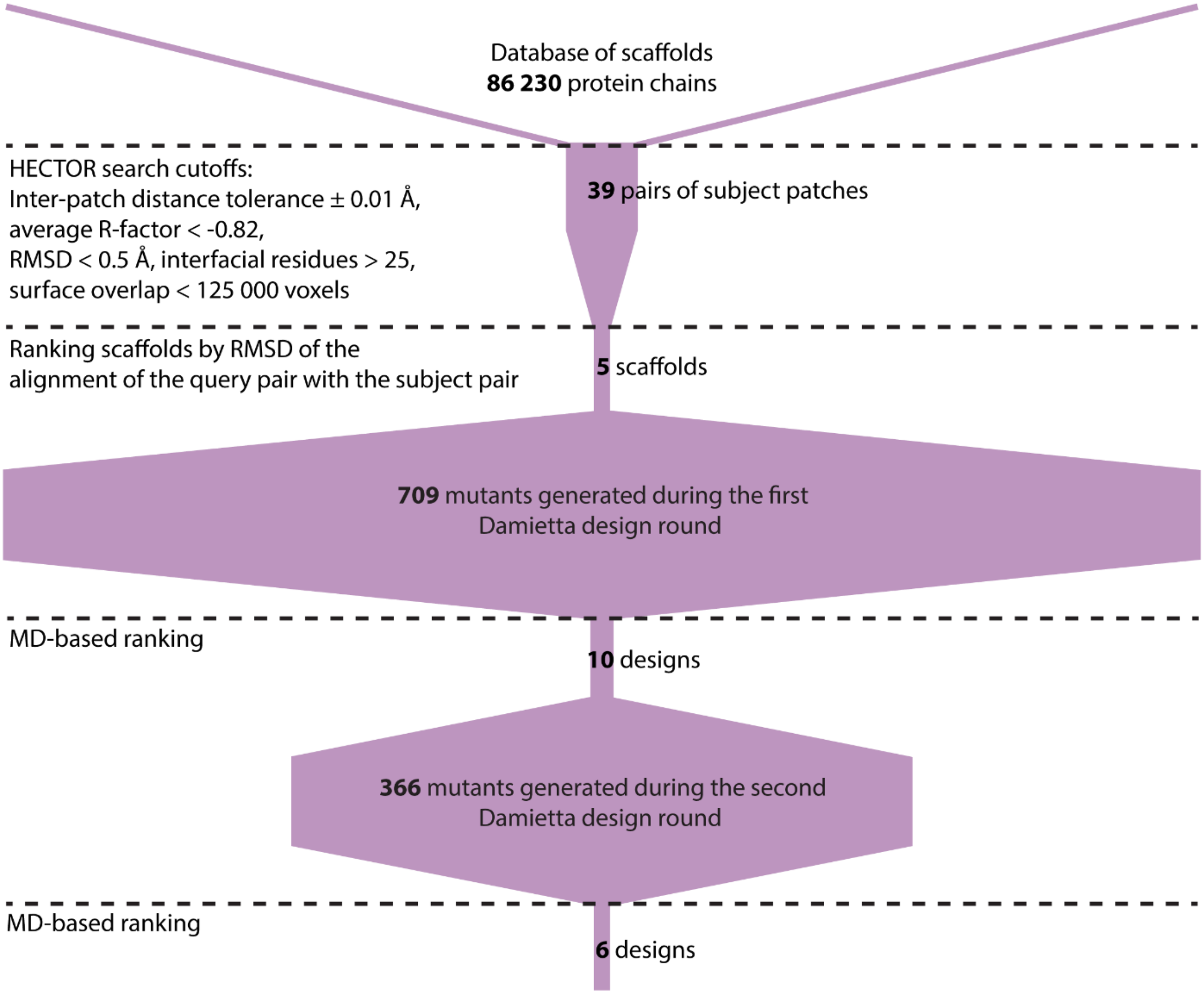

Figure S21. Filtering scheme for the process of *de novo* binder design. As an example, the design of binders against IL-7Rα site 1 is shown.

Figure S22

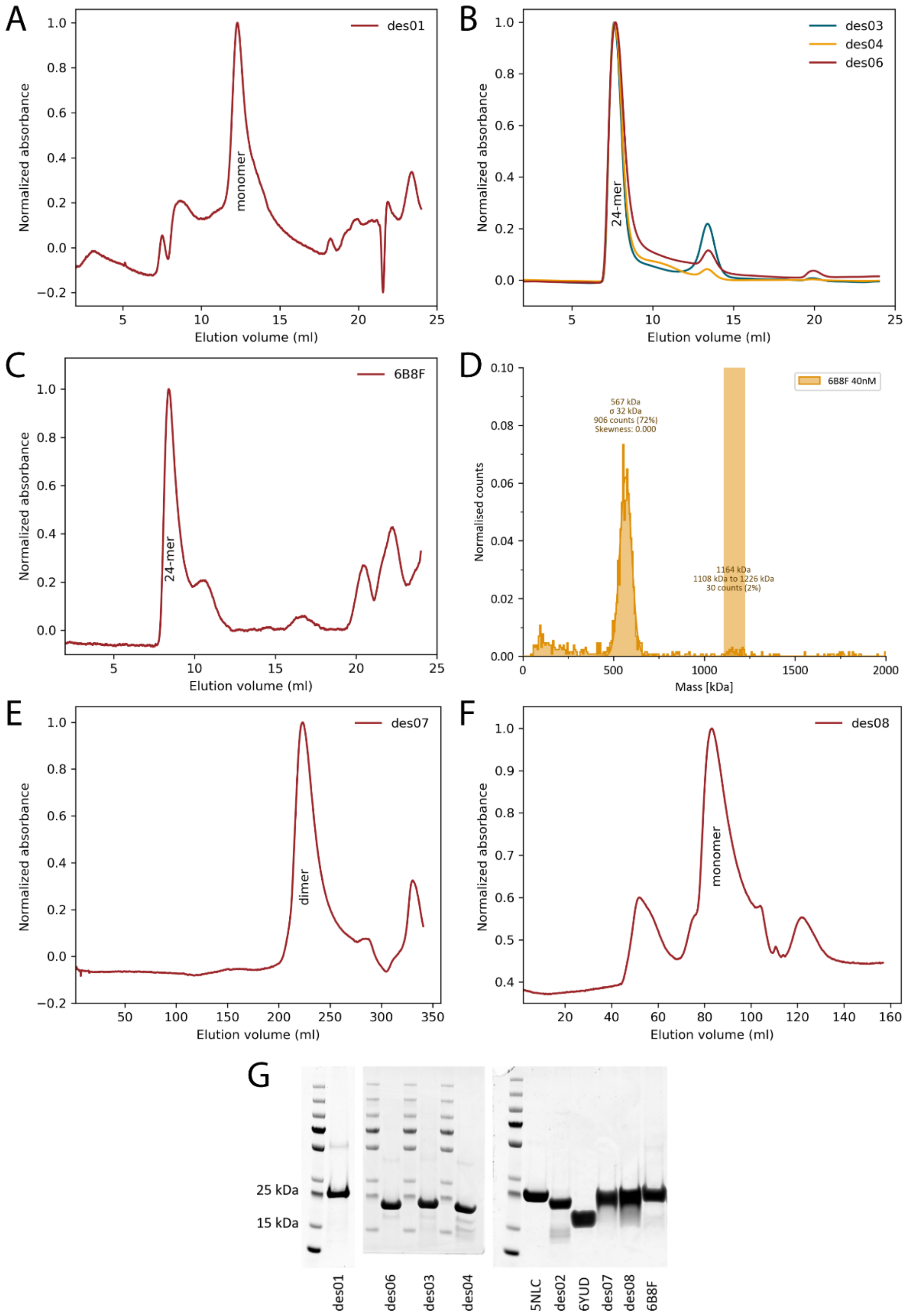

Supplementary figure 22. Purification of anti-IL-7Rα designs. Size exclusion chromatograms show that the final products of des01, and des08 elute as monomers **(A, F)**; the final product of des07 elutes as a dimer **(E)**; and the final products of des03, des04, and des06 elute as 24-mers **(B)**.

Size exclusion chromatogram **(C)** along with the mass photometry results **(D)** show that the ferritin template (6B8F) assembles into 24-mer, under the applied purification conditions. Size exclusion columns used to obtain presented chromatograms were: (A) Superdex 75 Increase 10/300 GL; (B,C) Superdex 200 Increase 10/300 GL; (E) HiLoad Superdex 75 26/600 pg; (F) HiLoad Superdex 75 16/600 pg. **(G)** SDS-PAGE of the final products of anti-IL-7Rα designs used for biophysical characterization.

Figure S23

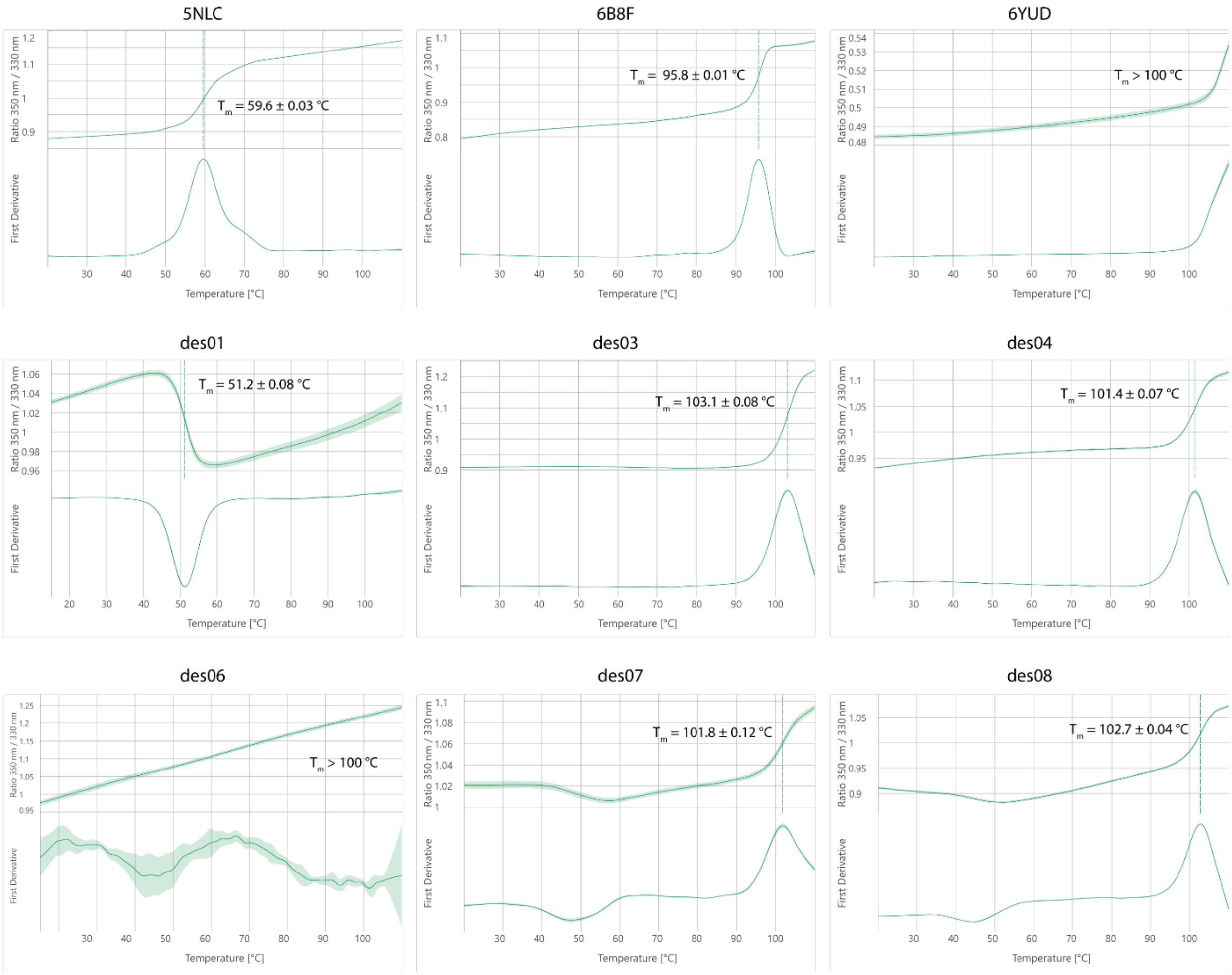

Supplementary figure 23. NanoDSF measurements for IL-7Rα binders and their templates.

Figure S24

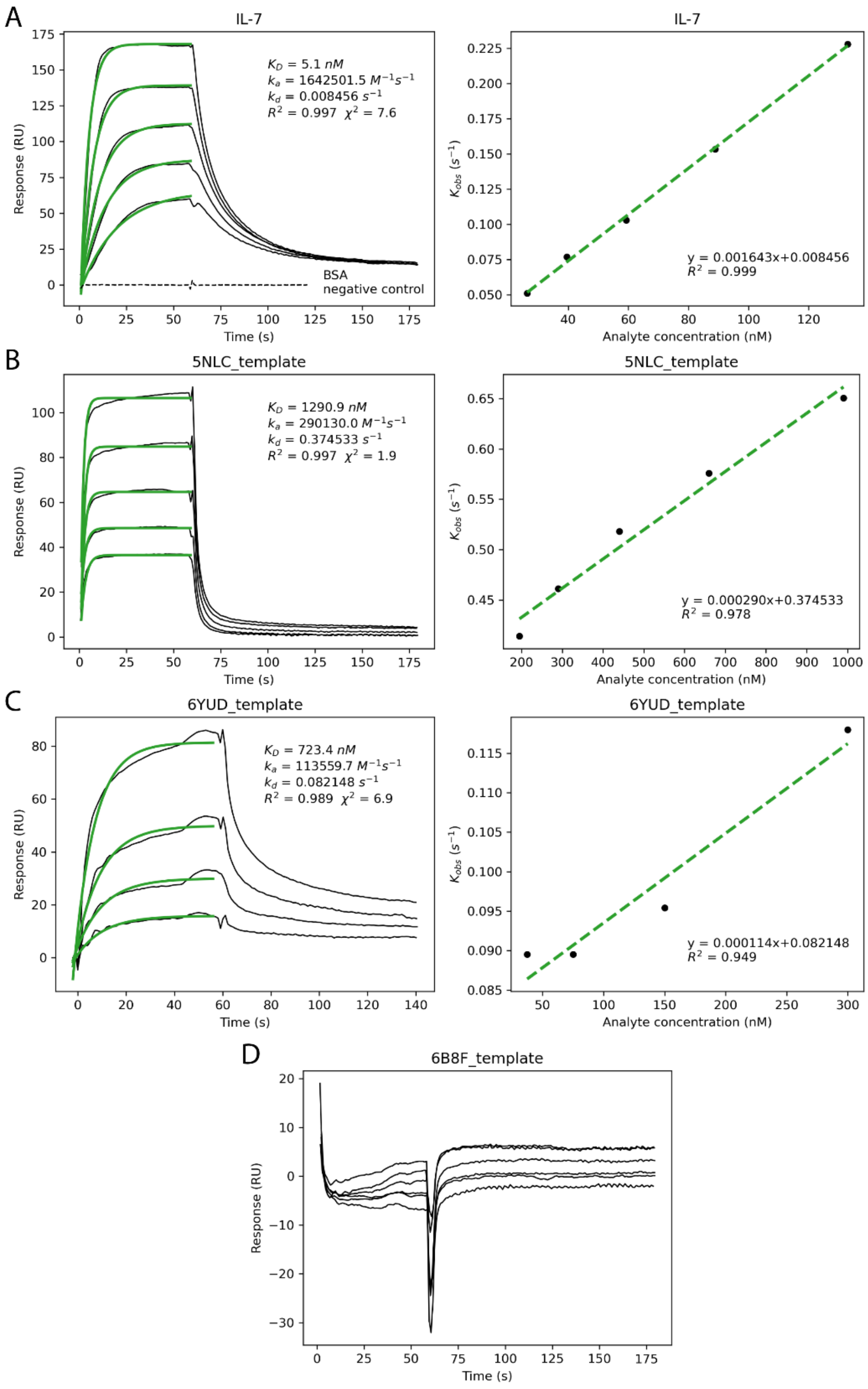

Supplementary figure 24. SPR sensograms of (A) IL-7, (B) 5NLC, (C) 6YUD, (D) 6B8F and their IL-7Rα binding kinetics fits. Sensograms and association phase fits are shown against their respective k_{obs} fits. Injection of 300 nM of BSA was used as negative control; the respective sensogram is show in pane (A).

Figure S25

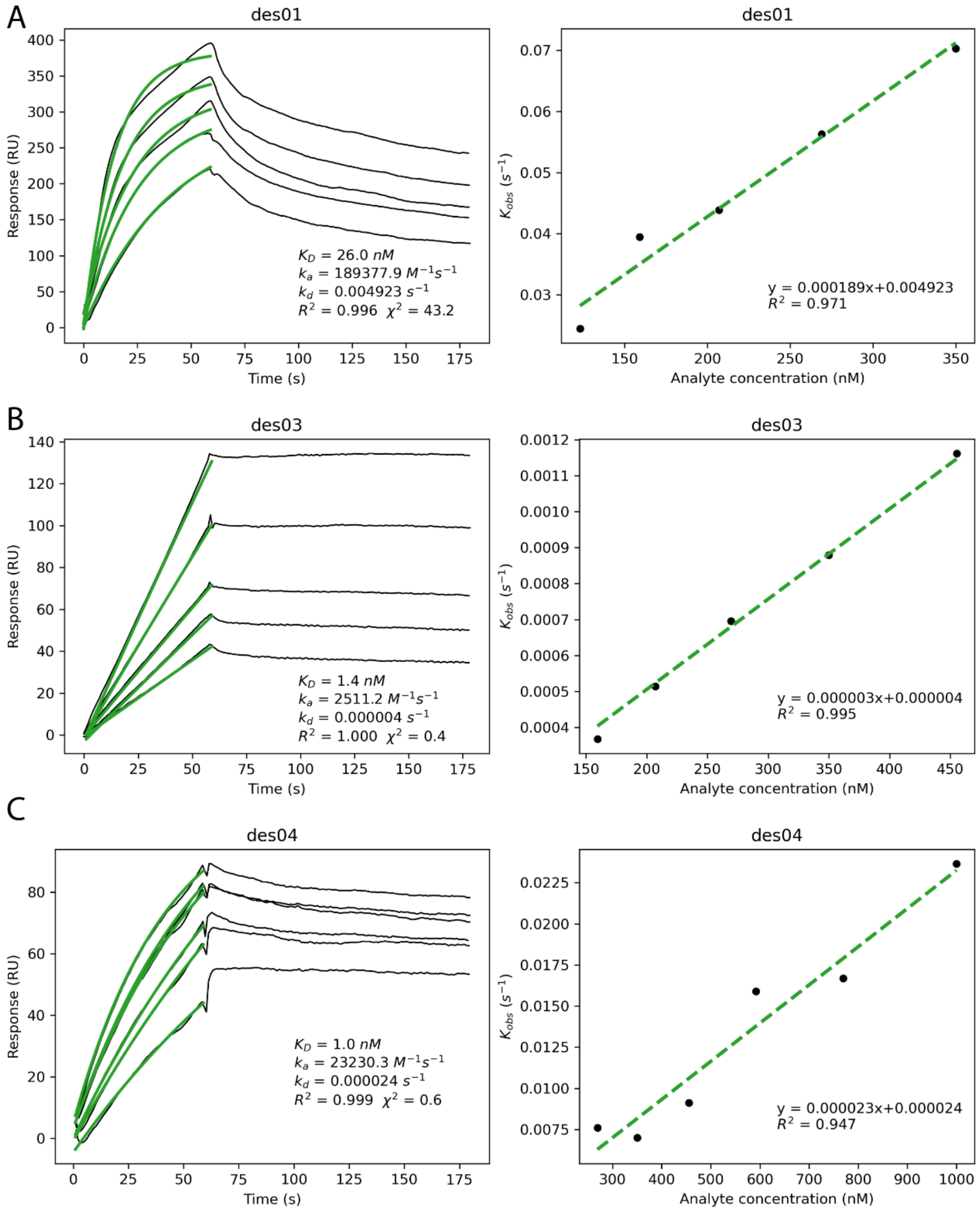

Supplementary figure 25. SPR sensograms of (A) des01, (B) des03, (C) des04 and their IL-7Rα binding kinetics fits. Sensograms (presented also in Fig. 4C) and association phase fits are shown against their respective k_{obs} fits.

Figure S26

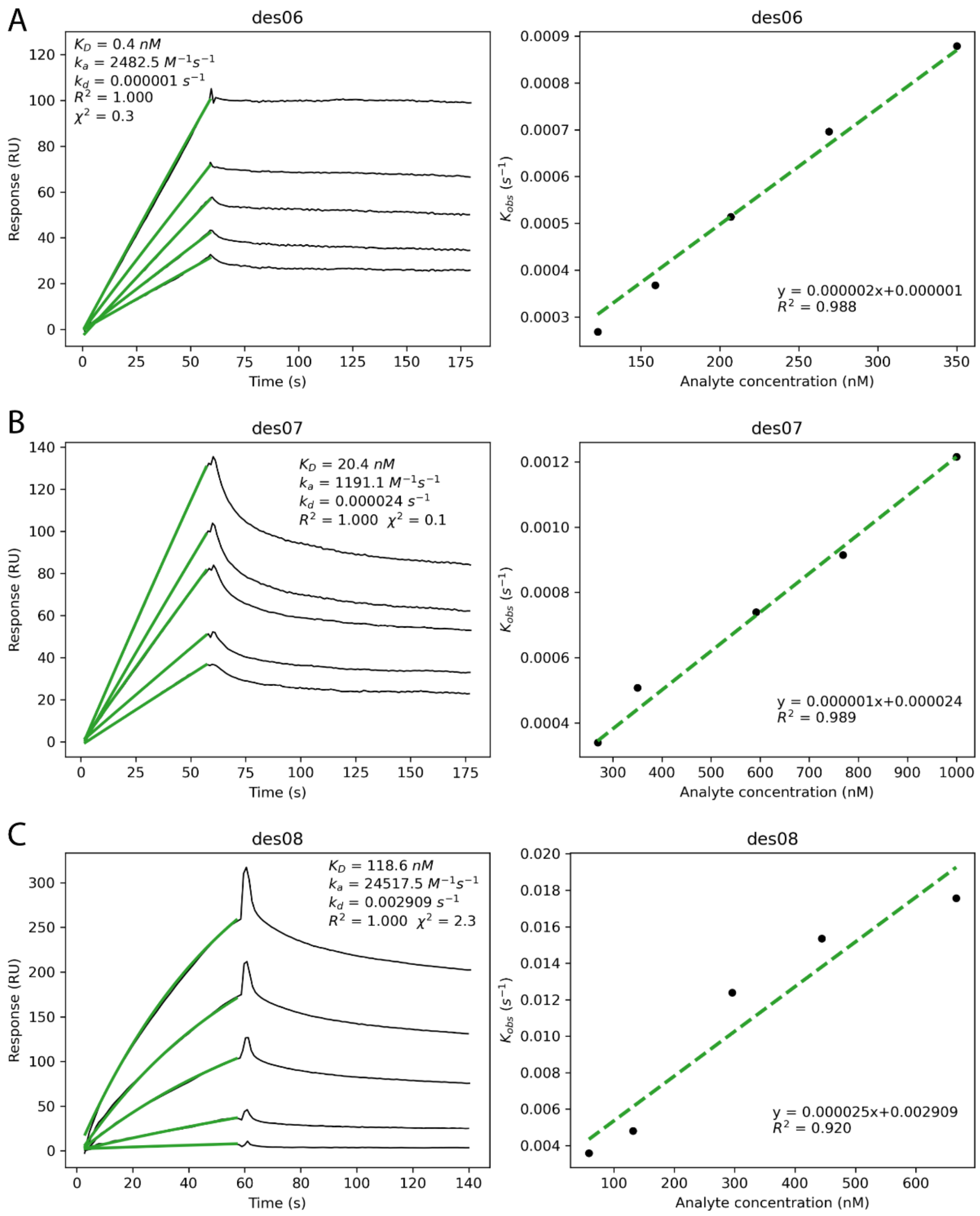

Supplementary figure 26. SPR sensograms of (A) des06, (B) des07, (C) des08 and their IL-7Rα binding kinetics fits. Sensograms (presented also in Fig. 4C) and association phase fits are shown against their respective k_{obs} fits.

Table S1. Final protein yield of anti-VEGF designs after two-step purification

**Name**	**Yield per litre of culture (mg)**
Sam0.1	4.0
Sam0.2	5.1
Sam0.3	5.2
Sam0.4	6.0
Sam0.5	13.2
Sam0.6	protein could not be neither purified from a soluble fraction, nor refolded
Sam0.7	43.8
Sam0.8	2.8
Sima1.1	1.1
Sima1.2	0.7
Sima2.1	0.7
Sima2.2	0.3
Sima3.1	2.6
Sima3.2	1.2
Sima4.1	0.7
Sima4.2	0.4

Table S2. Crystallographic Data Collection and Refinement Statistics

Structure	**Sam0.2**	**Sam0.7**
**Data collection**		
Space group	$P6_322$	$P6_522$
Cell parameters a, b, c (*Å*)	*77.81, 77.81, 78.16*	*40.55, 40.55, 253.12*
Wavelength (*Å*)	1.000	1.000
Resolution limits (*Å*)[a]	39.08-2.65 (2.81-2.65)	35.12-1.80 (1.91-1.80)
Unique reflections	4410 (686)	12500 (1934)
Completeness (%)	100 (100)	100 (99.9)
Redundancy	36.70 (40.09)	32.77 (34.94)
I/σI	31.5 (1.36)	19.0 (1.65)
R_{merge} (%)	8.4 (294.7)	10.3 (162.3)
CC(1/2)	100 (72.7)	99.9 (96.5)
**Refinement**		
Resolution limits (*Å*)	39.08-2.65	35.12-1.80
R_{cryst} (%)	25.7	25.7
R_{free} (%)	29.8	29.6
Protein molecules / asymmetric unit	1	1
Mean B value ($Å^2$)	110.5	50.0
**PDB code**	**8BL5**	**8BL9**

[a] Values in parenthesis refer to the highest-resolution shell.

Table S3. Split proteins used for HECTOR benchmarking

**PDB ID**	**Name**	**Query fragment**	**Subject fragment**	**Resolution (Å)**	**Reference**
1AXB	β-lactamase	26-196	198-290	2.0	[1]
1GD1	Holo-glyceraldehyde-3-phosphate dehydrogenase	1-148	149-333	1.8	[2]
1H5A	Horseradish peroxidase	1-213	214-306	1.6	[3]
1QE3	Esterase	2-94	95-484	1.5	[4]
1UBQ	Ubiquitin	1-35	36-76	1.8	[5]
3EST	Elastase	30-121, 234-245	16-29, 122-233	1.65	[2]
3RPT	Hydrolase	16-27, 123-230	28-122, 231-243	1.3	[2]

Table S4. Final protein yield of anti-IL-7Rα binders after two-step purification

Name	Yield per litre of culture (mg)
des01	0.6
des02	0.5
des03	4
des04	17
des05	0.2
des06	12
des07	2.4
des08	1.2

Table S5. Benchmarking of different combinations of HECTOR mapping parameters*

**Mapping parameters**	**Representative map**	**Enrichment factor in top 10 hits**	**Enrichment factor in top 100 hits**
Radius: 6 Å<br>Height: 6 Å<br>Resolution: 0.4 Å<br>Mapping frequency: 1:40<br>Mapping density: 1:8<br>Surface inflation: 0 Å<br>Bilinear interpolation<br>No fading	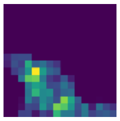	7.82 ± 10.91	0.78 ± 1.09
Radius: 6 Å<br>Height: 6 Å<br>Resolution: 0.2 Å<br>Mapping frequency: 1:5<br>Mapping density: 1:1<br>Surface inflation: 0 Å<br>No interpolation<br>No fading	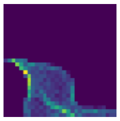	27.75 ± 40.40	13.88 ± 13.83
Radius: 12 Å<br>Height: 12 Å<br>Resolution: 0.4 Å<br>Mapping frequency: 1:40<br>Mapping density: 1:8<br>Surface inflation: 0 Å<br>Bilinear interpolation<br>No fading<br>**(HECTOR v0.1)**	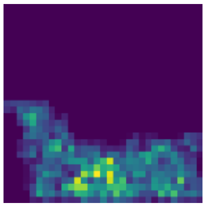	7.67 ± 3.08	0.85 ± 0.33
Radius: 12 Å<br>Height: 12 Å<br>Resolution: 0.2 Å<br>Mapping frequency: 1:5<br>Mapping density: 1:1<br>Surface inflation: 0 Å<br>No interpolation<br>No fading	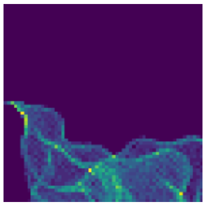	22.07 ± 19.85	18.52 ± 9.67

Radius: 10 Å<br>Height: 20 Å<br>Resolution: 0.2 Å<br>Mapping frequency: 1:5<br>Mapping density: 1:1<br>Surface inflation: 0 Å<br>No interpolation<br>No fading	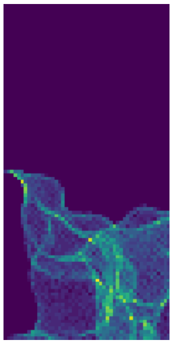	24.08 ± 17.78	18.97 ± 9.79
Radius: 10 Å<br>Height: 20 Å<br>Resolution: 0.2 Å<br>Mapping frequency: 1:5<br>Mapping density: 1:1<br>Surface inflation: 0.5 Å<br>No interpolation<br>Sigmoidal radial fading with a fade radius of 40 bins and a slope of 0.3<br>**(HECTOR v0.2)**	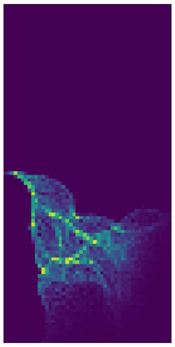	59.22 ± 42.14	24.86 ± 16.09
Radius: 20 Å<br>Height: 20 Å<br>Resolution: 0.2 Å<br>Mapping frequency: 1:5<br>Mapping density: 1:1<br>Surface inflation: 0.5 Å<br>No interpolation<br>No fading	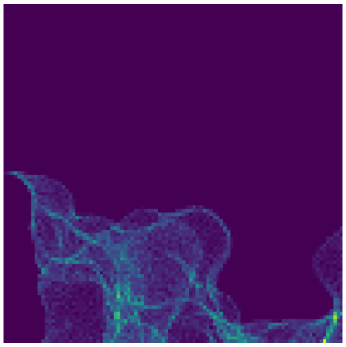	77.99 ± 85.24	36.06 ± 27.31

* Benchmarking was performed on a set of split proteins (1AXB, 1H5A, 1UBQ, 3EST; 45 pairs of query maps per protein) as described in Materials and methods under “HECTOR benchmarking”. Subject pairs were ranked based on the average R-factor calculated for two query maps. RMSD score was not considered in this benchmark.

Supplementary methods

1. PatchDock protocol
2. Rosetta protocol for design of VEGF binders
3. Damietta spec files for design of IL-7Rα binders
4. Example of NAMD input for serial tempering
5. Example of NAMD input for steered MD
6. Generating a lower-dimensional embedding of HECTOR maps

PatchDock protocol

```
import os
import sys

import subprocess as sp

#define software localisations
patchdock_location = 'PatchDock/'

# patchdock routines

def make_patchdock_parameter_file(receptor_name, ligand_name,
cluster_radius, outID, out_dict = out_docked_dict):

    outfile = '{}{}.params'.format(out_dict,outID)

    make_parm =
sp.Popen(["{}buildParamsToOutfile.pl".format(patchdock_location),
target_name, fragment_name, outfile, str(cluster_radius)], stdout=sp.PIPE,
stderr=sp.PIPE, stdin=sp.PIPE)
    stdout, stderr = make_parm.communicate()

    return outfile

def create_active_site_file(molecule_name, chain, active_site):

    active_site_file = '{}_active_site.txt'.format(molecule_name)
    ofile = open(active_site_file, "w")

    for residue in active_site:
        ofile.write("{} {}\n".format(residue, receptor_chain))
    ofile.close()

    return active_site_file

def add_active_site_to_patchdock_param_file(parameter_file,
active_site_file, mode = 'receptor'):

    with open(parameter_file, 'r') as pfile:
        data = pfile.readlines()
    pfile.close()

    for i in range(len(data)):
        if '#{}ActiveSite'.format(mode) in data[i]:
            data[i] = '{}ActiveSite {}\n'.format(mode, active_site_file)

    with open(parameter_file, 'w') as pfile:
        pfile.writelines(data)

    pfile.close()
    return

def generate_empty_patchdock_outfile(receptor_name, ligand_name, outID):
```

```
    # Shall generate a provisional patchdock outfile with the same
parameters as
    # those default from PatchDock and where no transformation is applied
to the ligand

    patchdock_outfile = '{}{}.dock_out'.format(out_docked_dict,outID)

    with open(patchdock_outfile, 'w') as outfile:


outfile.write('********************************************************\n
')
        outfile.write('Program parameters\n\n')
        outfile.write('baseParams    (Str)   4.0 13.0 2\n')
        outfile.write('clusterParams (Str)   0.1 4 2.0 4.0\n')
        outfile.write('desolvationParams    (Str)   500.0 1.0\n')
        outfile.write('ligandGrid    (Str)   0.5 6.0 6.0\n')
        outfile.write('ligandMs      (Str)   10.0 1.8\n')
        outfile.write('ligandPdb     (Str)   {}\n'.format(ligand_name))
        outfile.write('ligandSeg     (Str)   10.0 20.0 1.5 1 0 1 0\n')
        outfile.write('log-file      (Str)   patch_dock.log\n')
        outfile.write('log-level     (Str)   2\n')
        outfile.write('matchAlgorithm       (Str)   1\n')
        outfile.write('matchingParams       (Str)   1.5 1.5 0.4 0.5 0.9\n')
        outfile.write('protLib       (Str)   PatchDock/chem.lib\n')
        outfile.write('receptorGrid  (Str)   0.5 6.0 6.0\n')
        outfile.write('receptorMs    (Str)   10.0 1.8\n')
        outfile.write('receptorPdb   (Str)   {}\n'.format(receptor_name))
        outfile.write('receptorSeg   (Str)   10.0 20.0 1.5 1 0 1 0\n')
        outfile.write('scoreParams   (Str)   0.3 -5.0 0.5 0.0 0.0 1500 -8 -4
0 1 0\n\n')

outfile.write('********************************************************\n
\n')
        outfile.write('   # | score | pen.  | Area    | as1   | as2   |
as12  | ACE     | hydroph | Energy  |cluster| dist. || Ligand
Transformation\n')
        outfile.write('   1 | 00000 | 00000 | 0000000 | 00000 | 00000 |
00000 | 0000000 | 0000000 | 0000000 | 00000 | 00000 || 0.0 0.0 0.0 0.0 0.0
0.0\n')

    return patchdock_outfile


def dock(receptor_name, ligand_name, outID, cluster_radius = 4.0,
add_active_site = 'Yes', receptor_binding_site = None, ligand_binding_site
= None):

    # prepare parameter file
    parameter_file =  make_patchdock_parameter_file(receptor_name,
ligand_name, cluster_radius = cluster_radius, outID = outID, out_dict =
out_docked_dict)

    if add_active_site == 'Yes':
```

```
        receptor_active_site_file =
create_active_site_file(receptor_name.split('.')[0], receptor_chain = 'A',
active_site = receptor_binding_site)
        add_active_site_to_patchdock_param_file(parameter_file,
receptor_active_site_file)

        ligand_active_site_file =
create_active_site_file(ligand_name.split('.')[0], receptor_chain = 'A',
active_site = ligand_binding_site)
        add_active_site_to_patchdock_param_file(parameter_file,
ligand_active_site_file)

    # dock fragment to target
    patchdock_outfile = '{}{}.dock_out'.format(out_docked_dict,outID)
    run_patchdock =
sp.Popen(["{}patch_dock.Linux".format(patchdock_location), parameter_file,
patchdock_outfile], stdout=sp.PIPE, stderr=sp.PIPE, stdin=sp.PIPE)
    stdout, stderr = run_patchdock.communicate()

    # delete parameters file
    delete_params = sp.Popen(["rm", parameter_file], stdout=sp.PIPE,
stderr=sp.PIPE, stdin=sp.PIPE)
    stdout, stderr = delete_params.communicate()

    return patchdock_outfile



# ----------------------
# MAIN

receptor = sys.argv[1]
ligand   = sys.argv[2]
receptor_binding_site = sys.argv[3].split(',')
ligand_binding_site = sys.argv[4].split(',')

patchdock_outfile = dock(receptor, ligand, 'HECTOR', cluster_radius = 4.0,
add_active_site = 'Yes', receptor_binding_site = receptor_binding_site,
ligand_binding_site = ligand_binding_site)
```

Rosetta protocol for design of VEGF binders

```
<ROSETTASCRIPTS>
    <TASKOPERATIONS>
        <ReadResfile name="rrf" filename="%%resfile%%"/>
        <ProteinInterfaceDesign name="pid" repack_chain1="1"
repack_chain2="1" design_chain1="1" design_chain2="0"
interface_distance_cutoff="10"/>
        <IncludeCurrent name="currentTask"/>
        <RestrictToRepacking name="repackonly"/>
        <RestrictToInterfaceVector name="vectorTask" chain1_num="1"
chain2_num="2" CB_dist_cutoff="10.0" nearby_atom_cutoff="6.0"
vector_angle_cutoff="65.0" vector_dist_cutoff="8.0"/>
        <InitializeFromCommandline name="cmdTask"/>
    </TASKOPERATIONS>

    <FILTERS>
        <PackStat name="holes_1" threshold="%%pck_scr1%%" chain="0"
repeats="5"/>
        <PackStat name="holes_2" threshold="%%pck_scr2%%" chain="0"
repeats="5"/>
        <Ddg name="ddG" scorefxn="talaris2013" repack="true" threshold="-
20" repack_bound="true" repeats="2"/>
        <EnergyPerResidue name="nrgy_per_res" scorefxn="talaris2013"
score_type="total_score" whole_interface="1" jump_number="1"
interface_distance_cutoff="8.0" bb_bb="1"/>
        <ScoreType name="ttl_scr" scorefxn="talaris2013"
score_type="total_score" threshold="%%ttl_scr_thrshld%%"/>
    </FILTERS>

    <MOVERS>
        <DockingProtocol name="dock" docking_local_refine="1"
docking_score_high="soft_rep" ignore_default_docking_task="1" dock_min="1"
task_operations="vectorTask,cmdTask,repackonly,currentTask"/>
        <TaskAwareMinMover name="minmover_rpck" scorefxn="soft_rep"
chi="1" bb="1" jump="1"
task_operations="vectorTask,cmdTask,currentTask,repackonly"/>
        <GreedyOptMutationMover name="grdy_opt_mut" filter="ddG"
filter_delta="0.5" scorefxn="soft_rep" relax_mover="minmover_rpck"
sample_type="low" repack_shell="7.5" task_operations="rrf"/>
        <Backrub name="backrub"/>
        <BackrubDD name="backrubdd" partner1="1" partner2="0"
interface_distance_cutoff="8.0" moves="1000" sc_move_probability="0.2"
scorefxn="talaris2013" small_move_probability="0.3"
bbg_move_probability="0.4" task_operations="rrf"/>
        <RepackMinimize name="des1" scorefxn_repack="soft_rep"
scorefxn_minimize="soft_rep" minimize_bb="0" task_operations="rrf"
design_partner1="1" design_partner2="0"/>
        <RepackMinimize name="des2" scorefxn_repack="talaris2013"
scorefxn_minimize="talaris2013" minimize_bb="0" design_partner1="1"
design_partner2="0" task_operations="rrf"/>
        <RepackMinimize name="des3" design_partner1="1"
design_partner2="1" minimize_bb="0" task_operations="rrf"/>
        <FastRelax name="relax" scorefxn="talaris2013" repeats="2"
task_operations="currentTask,repackonly,cmdTask"/>
```

```
        <ParsedProtocol name="design">
            <Add mover_name="des1"/>
            <Add mover_name="backrubdd"/>
            <Add mover_name="des1"/>
            <Add mover_name="des2"/>
            <Add mover_name="des2"/>
            <Add mover_name="backrubdd"/>
            <Add mover_name="dock"/>
            <Add mover_name="des3"/>
            <Add filter="holes_1"/>
            <Add mover_name="backrub"/>
            <Add mover_name="des3"/>
            <Add filter="holes_2"/>
        </ParsedProtocol>
        <GenericMonteCarlo name="iterate" filter_name="ddG"
sample_type="low" scorefxn_name="talaris2013" mover_name="design"
trials="3"/>
        <GenericMonteCarlo name="iterate_h" filter_name="holes_1"
scorefxn_name="talaris2013" mover_name="design" trials="2"/>
    </MOVERS>

    <PROTOCOLS>
        <Add mover="iterate"/>
        <Add filter="holes_1"/>
        <Add mover="iterate_h"/>
        <Add filter="holes_2"/>
        <Add filter="ttl_scr"/>
        <Add filter="ddG"/>
    </PROTOCOLS>
</ROSETTASCRIPTS>


<!— This script was invoked as:

for fn in *.pdb; do for e in `seq 001 100`; do qsub -l h_vmem=2G -l
h_rt=15:0:0 -cwd -N des_fat -o ./ -b y
~/rstta_bin/rosetta_scripts.static.linuxgccrelease -database ~/rstta_db -s
gq01_0001.pdb -docking:dock_pert 1 2 -nstruct 1 -out:prefix $e -
out:file:silent silent_$e -out:file:scorefile score_$e -
parser:protocol ../intrfc_fixbb_10.xml -parser:script_vars
resfile='rf_gq01' pck_scr1='0.35' pck_scr2='0.45' ttl_scr_thrshld='-150.0'
-ignore_zero_occupancy false -mute all -ex1 -restore_talaris_behavior;
done; done -->
```

This protocol was run for 4-6 rounds in series with successively higher thresholds, where the output of each round was filtered by rosettaholes score and ddG and forwarded as input of the following round.

The resfiles for Sam/Sima designs were as follows.

- Sam resfile (input template: 1OH0):

```
NATAA
start
207 A ALLAAxc EX 1 LEVEL 3
210 A PIKAA EQ EX 1 LEVEL 3 EX 2 LEVEL 3 EX 3 LEVEL 1
```

```
211 A POLAR EX 1 LEVEL 3 EX 2 LEVEL 3 EX 3 LEVEL 1
214 A PIKAA RKQ EX 1 LEVEL 3 EX 2 LEVEL 3 EX 3 LEVEL 1
220 A APOLAR EX 1 LEVEL 3 EX 2 LEVEL 3
222 A POLAR EX 1 LEVEL 3 EX 2 LEVEL 3
238 A APOLAR EX 1 LEVEL 3 EX 2 LEVEL 3 EX 3 LEVEL 1
240 A APOLAR EX 1 LEVEL 3 EX 2 LEVEL 3 EX 3 LEVEL 1
242 A PIKAA YR EX 1 LEVEL 3 EX 2 LEVEL 3 EX 3 LEVEL 1
243 A POLAR EX 1 LEVEL 3
244 A ALLAAxc EX 1 LEVEL 3
245 A ALLAAxc EX 1 LEVEL 3
246 A ALLAAxc EX 1 LEVEL 3
252 A ALLAAxc EX 1 LEVEL 3
255 A ALLAAxc EX 1 LEVEL 3
257 A ALLAAxc EX 1 LEVEL 3
259 A ALLAAxc EX 1 LEVEL 3
260 A ALLAAxc EX 1 LEVEL 3
261 A PIKAA LM EX 1 LEVEL 3 EX 2 LEVEL 3 EX 3 LEVEL 1
263 A PIKAA QENDY EX 1 LEVEL 3 EX 2 LEVEL 3 EX 3 LEVEL 1
264 A POLAR EX 1 LEVEL 3 EX 2 LEVEL 3
265 A ALLAAxc EX 1 LEVEL 3
266 A ALLAAxc EX 1 LEVEL 3
268 A ALLAAxc EX 1 LEVEL 3
269 A ALLAAxc EX 1 LEVEL 3
281 A ALLAAxc EX 1 LEVEL 3
285 A POLAR EX 1 LEVEL 3 EX 2 LEVEL 3
288 A APOLAR EX 1 LEVEL 3 EX 2 LEVEL 3
290 A ALLAAxc EX 1 LEVEL 3
291 A ALLAAxc EX 1 LEVEL 3 EX 2 LEVEL 3
292 A ALLAAxc EX 1 LEVEL 3
293 A ALLAAxc EX 1 LEVEL 3
294 A ALLAAxc EX 1 LEVEL 3
297 A ALLAAxc EX 1 LEVEL 3 EX 2 LEVEL 3 EX 3 LEVEL 3
298 A ALLAAxc EX 1 LEVEL 3
301 A APOLAR EX 1 LEVEL 3 EX 2 LEVEL 3
302 A ALLAAxc EX 1 LEVEL 3
303 A APOLAR EX 1 LEVEL 3 EX 2 LEVEL 3
304 A ALLAAxc EX 1 LEVEL 3
318 A ALLAAxc EX 1 LEVEL 3
321 A ALLAAxc EX 1 LEVEL 3
322 A PIKAA EYLIW EX 1 LEVEL 3
325 A ALLAAxc EX 1 LEVEL 3 EX 2 LEVEL 3 EX 3 LEVEL 1
326 A ALLAAxc EX 1 LEVEL 3
```

- Sima resfile (input template: 1PM1):

```
NATAA
start

 24 A ALLAAxc
 27 A ALLAAxc
 29 A ALLAAxc
 31 A ALLAAxc
 32 A ALLAAxc
 33 A ALLAAxc
 34 A ALLAAxc
 35 A ALLAAxc
```

```
 36 A ALLAAxc
 38 A ALLAAxc
 40 A ALLAAxc
 53 A ALLAAxc
 55 A ALLAAxc
 57 A ALLAAxc
 59 A ALLAAxc
 61 A ALLAAxc
 62 A ALLAAxc
 66 A ALLAAxc
 67 A ALLAAxc
 68 A ALLAAxc
 70 A ALLAAxc
 84 A ALLAAxc
 85 A ALLAAxc
 87 A ALLAAxc
 96 A ALLAAxc
 98 A ALLAAxc
100 A ALLAAxc
101 A ALLAAxc
102 A ALLAAxc
104 A ALLAAxc
106 A ALLAAxc
118 A ALLAAxc
119 A ALLAAxc
120 A ALLAAxc
121 A ALLAAxc
122 A ALLAAxc
128 A ALLAAxc
129 A ALLAAxc
130 A ALLAAxc
132 A ALLAAxc
134 A ALLAAxc
136 A ALLAAxc
```

Damietta spec files for design of IL-7Rα binders

Input template 5NLC

```
library                         libv160_100/
input                           min_frm.pdb

# mutational targets
mut_res         238     GLU LYS GLN ARG
mut_res         239     GLU LYS GLN ARG
mut_res         242     GLU LYS GLN ARG
mut_res         237     PHE ILE LEU MET VAL TRP TYR
mut_res         249     PHE HIS ILE LEU MET GLN ARG VAL TRP TYR
mut_res         267     GLU LYS GLN ARG
mut_res         269     ALA ASP GLU PHE HIS ILE LYS LEU MET ASN PRO GLN ARG SER
THR VAL TRP TYR
mut_res         271     PHE ILE LEU MET VAL TRP TYR
mut_res         272     PHE HIS ILE LEU MET GLN ARG VAL TRP TYR
mut_res         283     GLU LYS GLN ARG
mut_res         295     PHE ILE LEU MET VAL TRP TYR ARG
mut_res         315     ASP GLU PHE HIS ILE LYS LEU MET ASN PRO GLN ARG SER THR
VAL TRP TYR
mut_res         339     ASP GLU PHE HIS ILE LYS LEU MET ASN PRO GLN ARG SER THR
VAL TRP TYR
mut_res         341     PHE HIS ILE LEU MET GLN ARG VAL TRP TYR
mut_res         343     SER THR ALA VAL
mut_res         360     GLU LYS GLN ARG
mut_res         364     GLU LYS GLN ARG
mut_res         373     GLU
mut_res         376     TYR GLN ARG
mut_res         378     PHE HIS ILE LEU MET GLN ARG VAL TRP TYR
mut_res         395     GLU LYS GLN ARG
mut_res         398     GLU LYS GLN ARG
mut_res         402     GLU LYS GLN ARG
mut_res         405     GLU LYS GLN ARG
mut_res         415     GLU ARG GLN
mut_res         417     PHE HIS ILE LEU MET GLN ARG VAL TRP TYR
mut_res         418     PHE HIS ILE LEU MET GLN ARG VAL TRP TYR


# repacking targets
rpk_res                 437
rpk_res                 47
rpk_res                 51
rpk_res                 52
rpk_res                 78
rpk_res                 79
rpk_res                 81
rpk_res                 97
rpk_res                 98
rpk_res                 100
rpk_res                 101
rpk_res                 102
rpk_res                 104
rpk_res                 124
rpk_res                 156
rpk_res                 158
```

```
rpk_res                 159
rpk_res                 161
rpk_res                 162
rpk_res                 211
rpk_res                 212
rpk_res                 213

# sampling parameters (optional)
scramble_order                  1
m_mutations                     3
n_paths                         6
n_iters                         5 # default:= 1

# mutagenesis scoring weights (optional)
mut_max_lj                      5.0
mut_w_pp                        1.0
mut_w_k                         1.0
mut_w_lj                        1.0
mut_w_solv                      1.0
mut_w_elec                      0.25

# repacking scoring weights (optional)
rpk_max_lj              5.0
rpk_w_pp                1.0
rpk_w_k                 1.0
rpk_w_lj                1.0
rpk_w_solv              1.0
rpk_w_elec              0.25

# speed up
load_memory     1
```

Input template 6B8F

```
library                         libv160_100/
input                           min_frm.pdb


# mutational targets
mut_res         242     GLU LYS GLN ARG
mut_res         243     ALA VAL
mut_res         245     GLU LYS GLN ARG
mut_res         246     ALA VAL SER THR
mut_res         249     PHE ILE LEU MET VAL TRP TYR
mut_res         252     PHE ILE LEU MET VAL TRP TYR
mut_res         253     PHE ILE LEU MET VAL TRP TYR
mut_res         310     ASP GLU HIS ILE LEU MET ASN GLN ARG SER THR VAL TRP TYR
mut_res         311     LYS GLN ARG
mut_res         313     ASP GLU HIS ILE LEU MET ASN GLN ARG SER THR VAL TRP TYR
mut_res         314     ASP GLU HIS
mut_res         316     ASN HIS TYR TRP
mut_res         332     HIS GLU LYS GLN ARG
mut_res         333     PHE ILE LEU MET VAL TRP TYR
mut_res         336     PHE ILE LEU MET VAL TRP TYR
mut_res         339     HIS GLU LYS GLN ARG TYR
mut_res         340     TYR MET THR HIS
mut_res         343     PHE ILE LEU MET VAL TRP TYR GLN GLU
```

```
# repacking targets
rpk_res                 47
rpk_res                 51
rpk_res                 52
rpk_res                 78
rpk_res                 79
rpk_res                 81
rpk_res                 97
rpk_res                 98
rpk_res                 100
rpk_res                 101
rpk_res                 102
rpk_res                 104
rpk_res                 124
rpk_res                 156
rpk_res                 158
rpk_res                 159
rpk_res                 161
rpk_res                 211
rpk_res                 212
rpk_res                 213

# sampling parameters (optional)
scramble_order          1
m_mutations             3
n_paths                 6
n_iters                 5 # default:= 1

# mutagenesis scoring weights (optional)
mut_max_lj                      5.0
mut_w_pp                        1.0
mut_w_k                         1.0
mut_w_lj                        1.0
mut_w_solv                      1.0
mut_w_elec                      0.25

# repacking scoring weights (optional)
rpk_max_lj              5.0
rpk_w_pp                1.0
rpk_w_k                 1.0
rpk_w_lj                1.0
rpk_w_solv              1.0
rpk_w_elec              0.25

# speed up
load_memory     1
```

Input template 6YUD

```
library                         libv160_100/
#library                        /home/gamacy/damietta/libv160_100
input                           min_frm.pdb
```

```
# mutational targets
mut_res         256     HIS PRO MET ILE PHE TYR TRP
mut_res         257     GLU THR PRO SER
mut_res         259     LEU MET
mut_res         260     ARG LYS
mut_res         281             PHE ILE LEU MET VAL TRP TYR
mut_res         285             PHE ILE LEU MET VAL TRP TYR
mut_res         286             PHE ILE LEU MET VAL TRP TYR
mut_res         288             LEU MET VAL
mut_res         290             LEU MET VAL TYR PHE
mut_res         309             MET ILE VAL
mut_res         310             MET ILE VAL TRP TYR

# repacking targets
rpk_res                 282
rpk_res                 45
rpk_res                 47
rpk_res                 48
rpk_res                 49
rpk_res                 89
rpk_res                 90
rpk_res                 91
rpk_res                 128
rpk_res                 130
rpk_res                 133
rpk_res                 134
rpk_res                 151
rpk_res                 153
rpk_res                 156
rpk_res                 157
rpk_res                 172
rpk_res                 175
rpk_res                 202
rpk_res                 204
rpk_res                 219
rpk_res                 220
rpk_res                 221
rpk_res                 222
rpk_res                 223
rpk_res                 225

# sampling parameters (optional)
scramble_order          1
m_mutations             3
n_paths                 6
n_iters                 5 # default:= 1

# mutagenesis scoring weights (optional)
mut_max_lj                      5.0
mut_w_pp                        1.0
mut_w_k                         1.0
mut_w_lj                        1.0
mut_w_solv                      1.0
mut_w_elec                      0.25

# repacking scoring weights (optional)
rpk_max_lj              5.0
rpk_w_pp                1.0
```

```
rpk_w_k                 1.0
rpk_w_lj                1.0
rpk_w_solv              1.0
rpk_w_elec              0.25

# speed up
load_memory    1
```

Example of NAMD input for serial tempering

```
structure                   complex.psf
coordinates                 complex.pdb
set temperature             290
set tmp_hi                  370
set tmp_lo                  250
set tmp_hi_stps             3000
set tmp_lo_stps             4000
set min_stps                100
set n_cycles                60
set dmp_freq                [expr { $tmp_hi_stps + $tmp_lo_stps + 2 * $min_stps } ]
set outputname              re_samin
firsttimestep               0
paraTypeCharmm              on
parameters               ../par_all36_prot_wtr_ions.prm
temperature              $temperature
gbis                     on
alphaCutoff                 12.0
ionConcentration            0.15
exclude                     scaled1-4
1-4Scaling                  1.0
Cutoff                      14.0
Switching                   on
Switchdist                  13.0
Pairlistdist                16.0
Timestep                    2.0
rigidBonds                  all
nonbondedFreq               1
fullElectFrequency          2
stepspercycle               10
langevin                    on
langevinDamping             1
langevinTemp                $temperature
langevinHydrogen            off
outputName                  $outputname
dcdfreq                     $dmp_freq
outputEnergies              500
outputPressure              500
constraints                 on
consexp                     5
consref                     complex.pdb
conskfile                   complex.pdb
conskcol                    B
constraintScaling           100
reinitvels                  $temperature
for { set cycle 0 } { $cycle < $n_cycles } { incr cycle } {
        minimize $min_stps
    langevinTemp $tmp_hi
        run $tmp_hi_stps
        langevinTemp $tmp_lo
        run $tmp_lo_stps
        minimize $min_stps
}
```

Example of NAMD input for steered MD

```
structure                ./structure.psf
coordinates              ./structure.pdb
extendedSystem           ./input.xsc
outputName               out_tst01
set temperature          310
firsttimestep            0
paraTypeCharmm           on
parameters               ../par_all36_prot_wtr_ions.prm
temperature              $temperature
wrapWater                on
wrapAll                  on
exclude                  scaled1-4
1-4scaling               1.0
cutoff                   12.0
switching                on
switchdist               10.0
pairlistdist             13.5
timestep                 1.0
rigidBonds               all
nonbondedFreq            1
fullElectFrequency       2
stepspercycle            10
PME                      yes
PMEGridSpacing           1.0
langevin                 on
langevinDamping          1
langevinTemp             $temperature
langevinHydrogen         no
useGroupPressure         yes
useFlexibleCell          no
useConstantArea          no
langevinPiston           on
langevinPistonTarget     1.01325
langevinPistonPeriod     100.
langevinPistonDecay      50.
langevinPistonTemp       $temperature
constraints              on
consref                  ./structure.pdb
conskfile                ./structure.pdb
conskcol                 B
constraintScaling        50.0
selectConstraints        on
selectConstrX            off
selectConstrY            off
selectConstrZ            on
SMD                      on
SMDFile                  ./structure.pdb
SMDk                     20
SMDVel                   0.000002
SMDDir                   .0 .0 1.0
SMDOutputFreq            10
minimize                 10000
reinitvels               $temperature
run                      7500000;
```

Generating a lower-dimensional embedding of HECTOR maps

To encode the surface maps from the original dimension of 50×100, a stacked convolutional autoencoder with ~9.7M parameters has been used. The output size of the encoder has been set to 128 and limited to a range of 0 to 1 with a sigmoid activation function. For the optimization, nine proteins (1GD1, 1I2M, 1JTG, 1KXP, 1UDI, 3RP2; 1CGI; 1YVB, 3EST) were split into 6/1/2 for training, validation and testing, respectively. Surface maps were generated for each protein providing a total of ~720K maps. The training was done by a combination of L1 and L2 loss for reconstruction, together with a triplet margin loss to improve the surface map latent representation of the encoder. Triplets were randomly generated, with each map acting as a query once and as a potential positive or negative multiple times. Training on the surface maps of the six proteins took 130 minutes on a NVIDIA Tesla V100.

References

1. Galarneau, A., et al., *Beta-lactamase protein fragment complementation assays as in vivo and in vitro sensors of protein protein interactions.* Nat Biotechnol, 2002. **20**(6): p. 619-22.
2. Ausiello, G., G. Cesareni, and M. Helmer-Citterich, *ESCHER: a new docking procedure applied to the reconstruction of protein tertiary structure.* Proteins, 1997. **28**(4): p. 556-67.
3. Martell, J.D., et al., *A split horseradish peroxidase for the detection of intercellular protein-protein interactions and sensitive visualization of synapses.* Nat Biotechnol, 2016. **34**(7): p. 774-80.
4. Jones, K.A., et al., *Development of a Split Esterase for Protein-Protein Interaction-Dependent Small-Molecule Activation.* ACS Cent Sci, 2019. **5**(11): p. 1768-1776.
5. Johnsson, N. and A. Varshavsky, *Split ubiquitin as a sensor of protein interactions in vivo.* Proc Natl Acad Sci U S A, 1994. **91**(22): p. 10340-4.
